# Observation of quantum information collapse-and-revival in a strongly-interacting Rydberg atom array

**DOI:** 10.1038/s41467-026-73520-3

**Published:** 2026-05-29

**Authors:** De-Sheng Xiang, Yao-Wen Zhang, Hao-Xiang Liu, Peng Zhou, Dong Yuan, Kuan Zhang, Shun-Yao Zhang, Biao Xu, Lu Liu, Yitong Li, Lin Li

**Affiliations:** 1 National Gravitation Laboratory, School of Physics, Huazhong University of Science and Technology, Wuhan, China; 2 Center for Quantum Information, IIIS, Tsinghua University, Beijing, China; 3 MOE Key Laboratory of Fundamental Physical Quantities Measurement, Huazhong University of Science and Technology, Wuhan, China; 4 Hubei Key Laboratory of Gravitation and Quantum Physics, Institute for Quantum Science and Engineering, Huazhong University of Science and Technology, Wuhan, China; 5 Lingang Laboratory, Shanghai, China; 6 Wuhan Institute of Quantum Technology, Wuhan, China

**Keywords:** Quantum information, Quantum simulation

## Abstract

Interactions of isolated quantum many-body systems typically scramble local information into the entire system and make it unrecoverable. Ergodicity-breaking systems possess the potential to exhibit fundamentally different information scrambling dynamics beyond this paradigm. For many-body localized systems with strong ergodicity breaking, local transport vanishes and information scrambles logarithmically slowly. Whereas in Rydberg atom arrays, local qubit flips induce dynamical retardation on surrounding qubits through the blockade effect, potentially leading to unconventional quantum information scrambling behaviours. Here, we present the measurements of out-of-time-ordered correlators and Holevo information in a Rydberg atom array, enabling us to precisely track quantum information scrambling and transport dynamics. By leveraging these tools, we observe a spatio-temporal *collapse-and-revival* behaviour of quantum information, which differs from both typical chaotic and many-body localized systems. Our experiment sheds light on the unique information dynamics in many-body systems with kinetic constraints, and demonstrates an effective digital-analogue approach to coherently reverse time evolution and steer information propagation in near-term quantum devices.

## Abstract (web summary)

The authors study out-of-time-ordered correlators and Holevo information in a Rydberg atom array, revealing the collapse-and-revival of quantum information in a constrained many-body system.

## Introduction

Quantum information scrambling—the spread of initial local information across the interacting many-body systems^1,2^—illuminates fundamental aspects in quantum thermalisation and chaos^3–8^, the black hole information paradox^9,10^ and quantum machine learning^11,12^. Understanding scrambling dynamics is also practically crucial for advancing quantum technologies for quantum simulation, benchmarking and metrology^13–16^. Recent works have revealed multiple information dynamics in quantum many-body systems, from rapid scrambling caused by chaotic non-integrable Hamiltonians^1,2^ to logarithmically slow information propagation in many-body localised systems^17–29^. Yet, the rich landscape of many-body physics, characterised by its diverse interaction forms and emergent phenomena, promises further unexplored regimes of information scrambling dynamics.

An intriguing frontier in this context is the quantum information dynamics within kinetically constrained systems, such as Rydberg atom arrays^30–45^. The strong van der Waals interaction between Rydberg atoms leads to the Rydberg blockade mechanism^46,47^, and constrains the local qubit flips. This essential physics can be approximately described by the PXP Hamiltonian^48,49^:

1 $${\widehat{H}}_{{{{\rm{PXP}}}}}={\sum }_{i}{\widehat{P}}_{i}{\widehat{\sigma }}_{i+1}^{x}{\widehat{P}}_{i+2}.$$

Here, ${\widehat{P}}_{i}=(1-{\widehat{\sigma }}_{i}^{z})/2$ is the projector onto the spin-down $\left|\downarrow \right\rangle$ state at site *i*, and ${\widehat{\sigma }}_{i}^{x,y,z}$ are Pauli matrices for the *i*-th spin. Previous studies of these systems have uncovered quantum many-body scars^49–53^. Moreover, recent theoretical investigations^54^ suggest that kinetically constrained many-body systems, such as those described by the PXP model, may harbour yet undiscovered mechanisms of information scrambling, opening up a new frontier for exploring unique spatio-temporal quantum information dynamics.

**Fig. 1:**
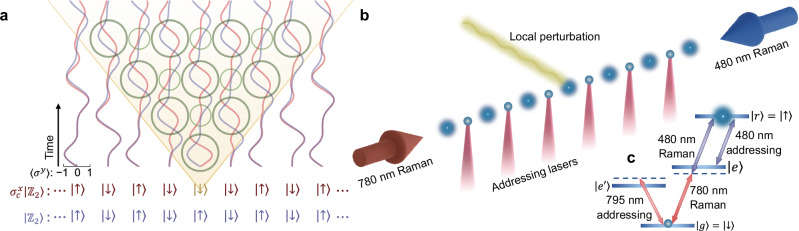
Illustrations of kinetically constrained qubit dynamics in the PXP model and its experimental realisation. **a** The blue and red curves display the evolution dynamics of ${\widehat{\sigma }}^{y}$ (the dynamics of other observables, such as ${\widehat{\sigma }}^{z}$, are presented in Section 5.2 of the Supplementary Information) expectation values for each qubit under the PXP Hamiltonian, starting from the initial states $\left|{{\mathbb{Z}}}_{2}\right\rangle$ and ${\widehat{\sigma }}_{c}^{x}\left|{{\mathbb{Z}}}_{2}\right\rangle$ (with the central spin being flipped), respectively. The initial rotation retardation near the central spin will propagate outwards in later dynamics. The yellow solid lines indicate where these two evolution dynamics begin to diverge, outlining a linear light cone (the yellow-shaded area). Within the light cone, spins resume constrained rotations, yet the distinguishability of spin states periodically vanishes (small dashed circles) and reappears (large dashed circles). See Supplementary Information Section 5.1 for more details on kinetically constrained qubit dynamics. **b** A one-dimensional, defect-free array of ^87^Rb atoms with approximately 7 μm spacing between neighbouring atoms. The spin rotation between the states $\left|\uparrow \right\rangle$ and $\left|\downarrow \right\rangle$ is achieved by driving the ground-Rydberg state transition via an off-resonant two-photon Raman process with 780-nm and 480-nm global excitation laser beams. Single-site qubit operations are realised by 795 and 480-nm addressing laser beams. **c** The inset shows relevant atomic levels: the ground state $\left|\downarrow \right\rangle=\left|g\right\rangle=\left|5{S}_{1/2},F=2,{m}_{F}=2\right\rangle$, the Rydberg state $\left|\uparrow \right\rangle=\left|r\right\rangle=\left|68{D}_{5/2},{m}_{J}=5/2\right\rangle$ and the intermediate states $\left|e\right\rangle=\left|5{P}_{3/2},F=3,{m}_{F}=3\right\rangle$ and $\left|e^{\prime} \right\rangle=\left|5{P}_{1/2}\right\rangle$. See Fig. 5 and Supplementary Information Section 1 for more experimental details.

Figure 1a depicts an intuitive understanding of the information spreading dynamics in this kinetically constrained system. In the PXP model, flipping the central spin in the antiferromagnetic Néel state $\left|{{\mathbb{Z}}}_{2}\right\rangle=\left|\uparrow \downarrow \uparrow \downarrow \uparrow \cdots \right\rangle$ encodes one bit of information. This local perturbation induces a retardation in the rotations of neighbouring spins, which then propagates ballistically, forming a linear light-cone-like wavefront (Supplementary Fig. 23). Within this light cone, spins resume their constrained rotations, yet the distinguishability of spin states periodically vanishes and reappears, as signified by differences in their ${\widehat{\sigma }}^{y}$ expectation values (dashed circles). This behaviour deviates significantly from typical chaotic quantum systems, where spin states inside the light cone quickly become indistinguishable due to scrambling. These kinetically constrained dynamics may lead to persistent information backflow and unusual breakdown of quantum chaos, hinting at new avenues for probing distinct information scrambling dynamics^54^. However, experimental exploration of quantum information dynamics in many-body systems presents significant challenges. Crucial tools such as out-of-time-ordered correlators (OTOCs) and Holevo information demand exceptional precision in state preparation, many-body evolution control and in situ state measurement^1,2^.

Here, we explore quantum information dynamics in a kinetically constrained Rydberg atom array. By developing and integrating experimental techniques, we overcome the above-mentioned challenges and successfully measure OTOCs and Holevo information for probing information scrambling. Notably, we observe the spatio-temporal *collapse-and-revival* of quantum information in a many-body system, revealing unique coherence properties where locally encoded information spreads across distant sites and remains periodically accessible despite strong many-body interactions. This behaviour differs significantly from typical chaotic and many-body localised systems, offering further insights into quantum scrambling and highlighting the potential of Rydberg atom arrays for quantum information processing, with dynamical quantum protection inherently provided by kinetic constraints.

## Results

### Kinetically constrained spin dynamics

As shown in Fig. 1b, c, our quantum simulator employs a linear array of up to 25 individual ^87^Rb atoms trapped in optical tweezers. The qubit states $\left|\downarrow \right\rangle$ and $\left|\uparrow \right\rangle$ are encoded in the atomic ground state $\left|g\right\rangle$ and a Rydberg state $\left|r\right\rangle$, respectively. The Hamiltonian governing the system reads:

2 $${\widehat{H}}_{{{{\rm{R}}}}}=\frac{\Omega }{2}{\sum }_{i}{\widehat{\sigma }}_{i}^{x}-\Delta {\sum }_{i}{\widehat{n}}_{i}+{\sum }_{i < j}{V}_{ij}{\widehat{n}}_{i}{\widehat{n}}_{j},$$

where *Ω* is the Rabi frequency of the coherent driving between $\left|\uparrow \right\rangle$ and $\left|\downarrow \right\rangle$, Δ is the detuning of driving lasers from the ground-Rydberg transition, ${\widehat{n}}_{i}=\left|{\uparrow }_{i}\right\rangle \left\langle {\uparrow }_{i}\right|$ is the projector towards the state $\left|{\uparrow }_{i}\right\rangle$, and ${V}_{ij}\propto 1/{R}_{ij}^{6}$ represents the van der Waals interaction between Rydberg atoms at sites *i* and *j* with the distance *R*_*ij*_. In the regime where *V*_*i*,*i* + 2_ ≪ *Ω* ≪ *V*_*i*,*i* + 1_, the Rydberg blockade prevents configurations with adjacent qubits both in the $\left|\uparrow \right\rangle$ state (e.g.$\left|\cdots {\uparrow }_{i}{\uparrow }_{i+1}\cdots \right\rangle$), making the system well-approximated by the PXP model in Eq. 1 and Supplementary Fig. 2.

**Fig. 2:**
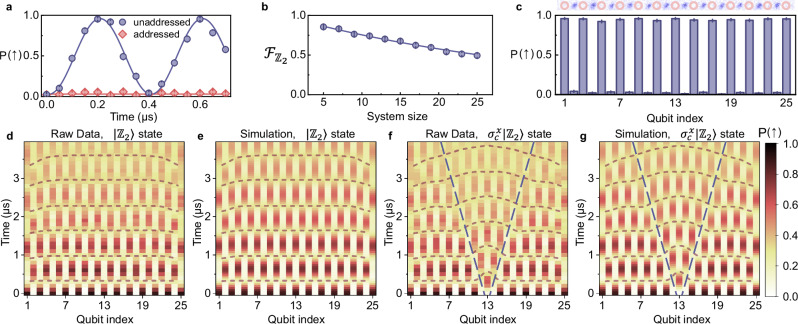
State preparation and kinetically constrained many-body state evolution. **a** Under the global coherent driving, the 795-nm-laser-addressed atoms (red) remain static, while the unaddressed atoms (blue) exhibit ground-Rydberg-state Rabi oscillations. **b** $\left|{{\mathbb{Z}}}_{2}\right\rangle$ state preparation fidelity ${{{{\mathcal{F}}}}}_{{{\mathbb{Z}}}_{2}}$ as a function of the system size. **c** Measured site-resolved Rydberg probability *P*_*i*_(*↑*) for a 25-qubit $\left|{{\mathbb{Z}}}_{2}\right\rangle$ state. Upper panel: atomic fluorescence illustration of the $\left|{{\mathbb{Z}}}_{2}\right\rangle$ state, where red circles denote Rydberg atoms. **d**–**g** Kinetically constrained many-body dynamics. **d**, **f** Measured site-resolved Rydberg state probability *P*_*i*_(*↑*) as a function of evolution time from the initial state $\left|{{\mathbb{Z}}}_{2}\right\rangle$ and ${\widehat{\sigma }}_{c}^{x}\left|{{\mathbb{Z}}}_{2}\right\rangle$, respectively. A linear light cone structure is observed in the ${\widehat{\sigma }}_{c}^{x}\left|{{\mathbb{Z}}}_{2}\right\rangle$ case (**f**), the origin of which is detailed in Section 2.3 of the Supplementary Information. **e**, **g** The corresponding results from numerical simulations. These simulations employ the full Rydberg Hamiltonian and take into account experimental noises based on our characterisation of these noise sources (see 'Methods', Section 4.3 of the Supplementary Information and Supplementary Fig. 17). Red dashed curves in (**d**–**g**) illustrate the propagating wavefronts, while blue dashed lines in (**f**, **g**) show the linear light cones. Error bars indicate the standard deviation given by the binomial distribution.

High-fidelity state preparation is essential for the success of all subsequent experiments. We prepare the $\left|{{\mathbb{Z}}}_{2}\right\rangle$ state by using the global Rydberg excitation lasers (780 and 480 nm) combined with site-selective 795-nm addressing lasers. The latter detune the addressed atoms from the ground-Rydberg transition, creating an alternating pattern of excitable and non-excitable atoms (Fig. 2a). As shown in Fig. 2b, c, our method achieves high $\left|{{\mathbb{Z}}}_{2}\right\rangle$ state preparation fidelities: 70(1)% for the 13 central qubits, and 49(3)% for the entire 25-qubit chain (Supplementary Fig. 3). After correcting for detection errors, the fidelities improve to 78(1)% for 13 qubits and 60(3)% for 25 qubits. Achieving such high preparation fidelity in a large Hilbert space (2^25^ dimensions) is critical for accurately probing kinetically constrained many-body state evolution and quantum information dynamics.

Next, we investigate the kinetically constrained spin dynamics described in the introduction (Supplementary Fig. 4), focusing on the evolution of the spin configurations $\left|{{\mathbb{Z}}}_{2}\right\rangle$ and ${\widehat{\sigma }}_{c}^{x}\left|{{\mathbb{Z}}}_{2}\right\rangle$ under the PXP Hamiltonian. For the $\left|{{\mathbb{Z}}}_{2}\right\rangle$ state (Fig. 2d), all spins rotate coherently under the PXP constraints, forming a uniform wavefront. This synchronised evolution, which is a hallmark of scar dynamics, persists until boundary effects gradually alter the spin rotations from the outer edge inwards.

In contrast, for the ${\widehat{\sigma }}_{c}^{x}\left|{{\mathbb{Z}}}_{2}\right\rangle$ state (Fig. 2f), we observe a clear linear light cone structure^55–58^. Spins outside the light cone remain unaffected by the flipped central spin, exhibiting dynamics similar to the $\left|{{\mathbb{Z}}}_{2}\right\rangle$ state. Within the light cone, the flipped central spin induces retardation in the evolution of neighbouring spins, creating a distinct evolving wavefront. This arc-shaped bent wavefront propagates outwards, driven by the retarded spin rotations, which clearly illustrates the constrained spin dynamics in the PXP model. The excellent agreement between experimental data and numerical results (Fig. 2e, g) confirms the effectiveness of our Rydberg quantum simulator in capturing the behaviour of kinetically constrained systems, paving the way for further explorations of quantum information dynamics.

### Out-of-time-ordered correlator dynamics

The out-of-time-ordered correlator is a powerful tool for probing quantum information scrambling in many-body systems^1,2^. However, due to considerable experimental challenges, OTOC measurements have been demonstrated only in several state-of-the-art physical platforms^13,14,16,59–70^. In the Rydberg atom array, we experimentally employ OTOCs to investigate the information scrambling dynamics under kinetic constraints. The OTOC is defined as follows, which quantifies how the operator growth of a local perturbation $\widehat{W}$ (called the butterfly operator) spreads throughout the lattice sites:

3 $${\widehat{F}}_{ij}(t)=\left\langle \psi \right|{\widehat{W}}_{i}^{{{\dagger}} }(t){\widehat{V}}_{j}^{{{\dagger}} }{\widehat{W}}_{i}(t){\widehat{V}}_{j}\left|\psi \right\rangle .$$

**Fig. 3:**
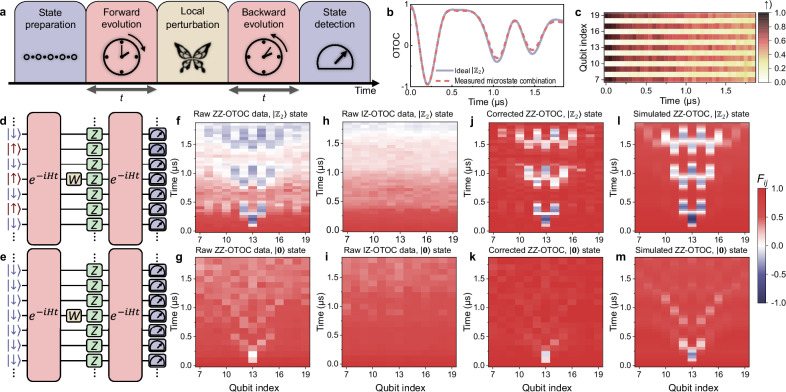
Probing quantum information scrambling dynamics via OTOCs. **a** An illustration of the OTOC measurement protocol. **b** The solid and dashed lines correspond to numerically simulated OTOC dynamics of the central qubit with the perfect $\left|{{\mathbb{Z}}}_{2}\right\rangle$ state and the experimentally measured microstate combination (with ${{{{\mathcal{F}}}}}_{{{\mathbb{Z}}}_{2}}=78(1)\%$) as inputs, respectively. **c** Forward-and-backwards Hamiltonian evolution of the experimentally prepared $\left|{{\mathbb{Z}}}_{2}\right\rangle$ state. The observed decay of the time reversal arises from both imperfections in the inversion of the Rydberg Hamiltonian evolution (see Supplementary Information Section 2.2 for details) and experimental noises (see Supplementary Information Section 4 for detailed analysis and Supplementary Fig. 17 for numerical simulations). **d**, **e** Illustration of the digital-analogue scheme for OTOC measurements with $\left|{{\mathbb{Z}}}_{2}\right\rangle$ and $\left|{{{\bf{0}}}}\right\rangle$ states, using $\widehat{W}={\widehat{\sigma }}_{c}^{z}$ for ZZ-OTOC and $\widehat{W}=\widehat{I}$ for IZ-OTOC. **f**–**m** Spatio-temporal OTOC dynamics. The colour bar corresponds to the values of the OTOC, ${\widehat{F}}_{ij}(t)$. **f**, **g** Measured ZZ-OTOC for $\left|{{\mathbb{Z}}}_{2}\right\rangle$ and $\left|{{{\bf{0}}}}\right\rangle$ states, respectively. **h**, **i** Measured IZ-OTOC for $\left|{{\mathbb{Z}}}_{2}\right\rangle$ and $\left|{{{\bf{0}}}}\right\rangle$ states, respectively. **j**, **k** Corrected ZZ-OTOC for $\left|{{\mathbb{Z}}}_{2}\right\rangle$ and $\left|{{{\bf{0}}}}\right\rangle$ states. **l**, **m** Numerically simulated ZZ-OTOC for $\left|{{\mathbb{Z}}}_{2}\right\rangle$ and $\left|{{{\bf{0}}}}\right\rangle$ states using the ideal Rydberg Hamiltonian (see 'Methods'). The OTOC features in corrected experimental data (**j**, **k**) are slightly less pronounced than in simulations (**l**, **m**). This difference arises because the simulations do not account for experimental noise stemming from imperfections in the local perturbation ${\widehat{\sigma }}_{c}^{z}$. These imperfections degrade the OTOC features but cannot be corrected using IZ-OTOC. For a detailed comparison between the corrected experimental data and simulations that include these imperfections, see Fig. 6 and Supplementary Fig. 19 (Supplementary Information Section 4).

Our study focuses on the ZZ-OTOC, with local butterfly operator ${\widehat{W}}_{i}={\widehat{\sigma }}_{c}^{z}$ (Supplementary Figs. 8 and 16), and measurement operator ${\widehat{V}}_{j}={\widehat{\sigma }}_{j}^{z}$, in order not to violate the kinetically constrained spin rotations. ${\widehat{W}}_{i}(t)={e}^{i\widehat{H}t}{\widehat{W}}_{i}{e}^{-i\widehat{H}t}$ represents the Heisenberg evolution of ${\widehat{W}}_{i}$, and $\left|\psi \right\rangle$ is an initial state. The local perturbation, corresponding to a ${\widehat{\sigma }}_{c}^{z}$ gate (a *π*-phase shift) on the central qubit, is experimentally implemented via the light shift from a 795-nm addressing laser. Figure 3a shows the five-step protocol for measuring the OTOC ${\widehat{F}}_{ij}(t)$: (1) state preparation; (2) forward evolution; (3) local perturbation; (4) backward (time-reversed) evolution; and (5) computational basis measurements (Supplementary Fig. 7).

Here, we employ two distinct initial states to study the OTOC dynamics, including the antiferromagnetic ${{\mathbb{Z}}}_{2}$-ordered Néel state $\left|{{\mathbb{Z}}}_{2}\right\rangle=\left|\uparrow \downarrow \uparrow \downarrow \uparrow \cdots \right\rangle$ (which has large overlap with the scarred eigenstate subspace^49^) and a trivial product state $\left|{{{\bf{0}}}}\right\rangle=\left|\downarrow \downarrow \downarrow \downarrow \downarrow \cdots \right\rangle$ (which lies in the common thermal eigenstate subspace). Our numerical simulations show that in a small-size spin chain (less than 10 qubits), boundary effects observed in Fig. 2 can lead to considerable deviations from the ideal PXP dynamics (Supplementary Information Section 2.3 and Supplementary Fig. 6). To mitigate the boundary and finite-size effects, we particularly prepare a 25-qubit $\left|{{\mathbb{Z}}}_{2}\right\rangle$ ($\left|{{{\bf{0}}}}\right\rangle$) state and perform the OTOC experiments on the central 13 qubits. This configuration shields the region of interest from boundary effects during evolution, better approximating the bulk PXP dynamics. Moreover, our high $\left|{{\mathbb{Z}}}_{2}\right\rangle$ state preparation fidelity is crucial for accurately probing OTOC dynamics. Low preparation fidelity introduces numerous error states into the spin chain, which smear the expected OTOC patterns (Supplementary Information Section 4.1 and Supplementary Figs. 13–15). As a benchmark, the numerically simulated OTOC dynamics using the experimentally measured microstate combination as input (dashed lines in Fig. 3b) show a negligible difference from the results obtained with the perfect $\left|{{\mathbb{Z}}}_{2}\right\rangle$ initial state (solid lines in Fig. 3b), attesting to the accuracy of our experimental approach.

With the initial states well prepared, we address the next crucial and notoriously difficult step in OTOC measurements—implementing the time-reversed evolution under $-\widehat{H}$ in the many-body system. While reversing single-particle Hamiltonians is relatively easy, reversing many-body interaction terms, such as switching between attractive and repulsive interactions, presents significantly more complex theoretical and experimental challenges. For instance, in the quest to achieve time reversal using a digital simulation approach, Google’s Sycamore quantum computer^14^ leverages its cutting-edge gate fidelities and large circuit depths, and employs well-designed quantum circuits to implement the process.

In contrast, our approach exploits the particle-hole symmetry of the PXP model, enabling us to implement the inverse Hamiltonian through a global ${\widehat{\sigma }}^{z}$ gate on all qubits: $({\prod }_{i}{\widehat{\sigma }}_{i}^{z}){\widehat{H}}_{{{{\rm{PXP}}}}}({\prod }_{i}{\widehat{\sigma }}_{i}^{z})=-{\widehat{H}}_{{{{\rm{PXP}}}}}$. This is experimentally realised by using a far-detuned microwave field that induces a *π*-phase shift on the Rydberg states. To bridge the gap between the full Rydberg Hamiltonian ${\widehat{H}}_{{{{\rm{R}}}}}$ and the ideal PXP model ${\widehat{H}}_{{{{\rm{PXP}}}}}$, we conducted detailed benchmark simulations and optimised the experimental parameters (see Section 2.2 of the Supplementary Information for more details). For instance, a small global detuning was introduced to compensate for next-nearest-neighbour Rydberg interactions. These optimisations allow the experimental Hamiltonian to closely approximate the PXP model and enable effective time reversal. Figure 3c shows the experimentally measured forward-and-backwards evolution dynamics of the $\left|{{\mathbb{Z}}}_{2}\right\rangle$ state. This digital-analogue protocol generally serves as a powerful technique in Rydberg atom arrays to coherently reverse many-body evolution and probe information scrambling dynamics.

Having achieved the high-fidelity state preparation and time reversal, we executed the complete OTOC measurement protocol (Fig. 3d, e), demonstrating precise control over complex quantum many-body dynamics in our Rydberg atom array. Figure 3f, g present the experimentally measured spatio-temporal evolution of the ZZ-OTOCs for the initial states $\left|{{\mathbb{Z}}}_{2}\right\rangle$ and $\left|{{{\bf{0}}}}\right\rangle$, respectively. The overall decay of the measured OTOC values primarily stems from atomic state losses and decoherence during the evolution (see Supplementary Information Section 4), even in the absence of quantum information scrambling. In order to distinguish the scrambling dynamics of interest from these extraneous decay mechanisms^63^, we additionally measure the evolution of both $\left|{{\mathbb{Z}}}_{2}\right\rangle$ and $\left|{{{\bf{0}}}}\right\rangle$ states under the action of $\widehat{H}$ and $-\widehat{H}$ but without any butterfly operator, i.e. the IZ-OTOCs, where I stands for the identity operator. Based on the dynamics of the IZ-OTOCs (Fig. 3h, i), we employ error mitigation techniques similar to those developed in previous work^14^ to correct the raw ZZ-OTOC data (see Supplementary Information Section 4). The resulting corrected OTOC dynamics (Fig. 3j, k) align well with the numerical simulations based on ideal Rydberg Hamiltonian evolution (Fig. 3l, m).

The OTOC evolution dynamics reveal strikingly different behaviours for the $\left|{{{\bf{0}}}}\right\rangle$ and $\left|{{\mathbb{Z}}}_{2}\right\rangle$ initial states. For the $\left|{{{\bf{0}}}}\right\rangle$ state, the OTOCs quickly decay inside the linear light cone without discernible revivals. This rapid information scrambling agrees with theoretical expectations for generic high-energy initial states in chaotic quantum systems. In stark contrast, the $\left|{{\mathbb{Z}}}_{2}\right\rangle$ state exhibits a slower butterfly velocity, and remarkably, a spatio-temporal *collapse-and-revival*-like pattern of quantum information inside the linear light cone.

The *collapse-and-revival* of wavefunctions has been previously observed in systems such as the Jaynes-Cummings model^71,72^, reflecting the periodic dispersion and recurrence of quantum coherence. In contrast to conventional expectations for many-body systems, where interactions rapidly scramble quantum information across an exponentially large Hilbert space, our observations reveal a striking preservation and refocusing of quantum coherence inside the linear light cone. These scrambling dynamics suggest that kinetically constrained systems allow for ballistic propagation and periodic recovery of quantum information, which differs significantly from both typical chaotic and many-body localised systems.

### Holevo information dynamics

In addition to OTOCs, we employ Holevo information to investigate quantum information dynamics in our system. While ZZ-OTOCs have successfully revealed the *collapse-and-revival* phenomenon, the ${\widehat{\sigma }}^{z}$ perturbation can become ineffective at certain conditions. This is evident in Fig. 3j, l, where OTOC values for all qubits approach 1 during the intervals 0.6–0.7 μs and 1.2–1.3 μs. At these times, the perturbed qubit is close to a pure $\left|\uparrow \right\rangle$ or $\left|\downarrow \right\rangle$ state, rendering the butterfly operator ineffective (see Supplementary Information Section 3.2 and Supplementary Fig. 9 for details).

To gain a more continuous view of information propagation in kinetically constrained systems, we turn to Holevo information. Originally proposed to upper bound the accessible information between two separate agents^73^, Holevo information can be utilised to quantify the local distinguishability of quantum states evolving from slightly different initial conditions. By treating the reduced Hamiltonian evolution on subsystems as quantum channels, we use Holevo information to complement OTOC measurements, particularly in scenarios where butterfly operator perturbations become less effective. This approach, leveraging quantum state tomography, provides a more comprehensive understanding of quantum information dynamics in our system.

**Fig. 4:**
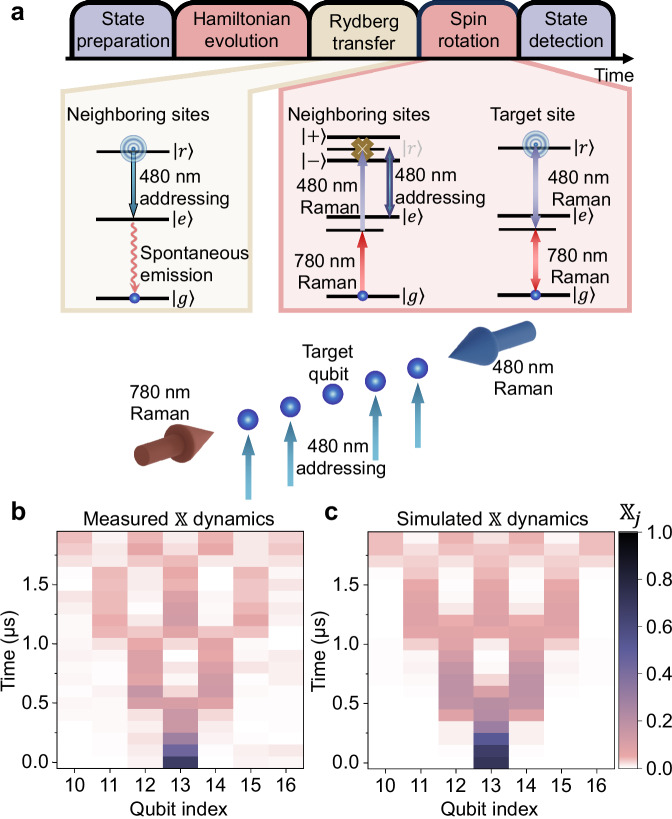
Probing quantum information transport dynamics via Holevo information. **a** Illustration of the Holevo information measurement protocol. In order to measure the off-diagonal elements of the reduced density matrices, the 480-nm addressing lasers first transfer Rydberg populations at the neighbouring sites to the ground state $\left|g \right\rangle$, and then split the $\left\vert r\right\rangle$ state into two dressed states $\left\vert +\right\rangle=(\left\vert r\right\rangle+\left\vert e\right\rangle)/\sqrt{2}$ and $\left\vert -\right\rangle=(\left\vert r\right\rangle-\left\vert e\right\rangle)/\sqrt{2}$, thus shielding these sites from spin rotation under global driving. See Supplementary Information Section 3.2 for further details on quantum state tomography under spin rotation constraints. **b**, **c** Experimentally measured and numerically simulated spatio-temporal dynamics of the Holevo information, respectively. The colour bar corresponds to the values of the Holevo information ${{\mathbb{X}}}_{j}(t)$. The numerical simulation utilises the Rydberg Hamiltonian ${\widehat{H}}_{{{{\rm{R}}}}}$ and considers various experimental imperfections based on the precise characterisation of our system (see 'Methods' and Supplementary Information Section 4.3).

Our experiment explores two sets of Hamiltonian evolution applied to different initial states: $\left|{{\mathbb{Z}}}_{2}\right\rangle$ and ${\widehat{\sigma }}_{c}^{x}\left|{{\mathbb{Z}}}_{2}\right\rangle$. The ${\widehat{\sigma }}_{c}^{x}$ operator acts on the central qubit in the one-dimensional chain, encoding one bit of local information in the initial states, as illustrated in Fig. 1a. During subsequent Hamiltonian evolution, we measure the Holevo information ${{\mathbb{X}}}_{j}(t)$ at different sites *j*, which quantifies how much information encoded on the central qubit can be retrieved through local measurements at distant sites:

4 $${{\mathbb{X}}}_{j}(t)=S\left(\frac{{\widehat{\rho }}_{j}(t)+{\widehat{\rho }}_{j}^{{\prime} }(t)}{2}\right)-\frac{S({\widehat{\rho }}_{j}(t))+S({\widehat{\rho }}_{j}^{{\prime} }(t))}{2},$$

where ${\widehat{\rho }}_{j}(t)$ and ${\widehat{\rho }}_{j}^{{\prime} }(t)$ are reduced density matrices of the *j*-th spin after Hamiltonian evolution for the initial states $\left|{{\mathbb{Z}}}_{2}\right\rangle$ and ${\widehat{\sigma }}_{c}^{x}\left|{{\mathbb{Z}}}_{2}\right\rangle$, respectively, and $S\left(\widehat{\rho }\right)=-{{{\rm{Tr}}}}\left(\widehat{\rho }\log \widehat{\rho }\right)$ is the von Neumann entropy. The experimental measurement procedure is shown in Fig. 4a, which includes: (1) preparation of initial states $\left|{{\mathbb{Z}}}_{2}\right\rangle$ and ${\widehat{\sigma }}_{c}^{x}\left|{{\mathbb{Z}}}_{2}\right\rangle$; (2) Hamiltonian evolution over time *t*; (3) quantum state tomography on the qubit *j* to obtain ${\widehat{\rho }}_{j}(t)$ and ${\widehat{\rho }}_{j}^{{\prime} }(t)$ (Supplementary Figs. 10 and 11).

Performing full quantum state tomography under the PXP kinetic constraints is highly challenging. While the diagonal elements of ${\widehat{\rho }}_{j}(t)$ and ${\widehat{\rho }}_{j}^{{\prime} }(t)$ can be directly extracted from Rydberg population measurements (Fig. 2d, f), obtaining the off-diagonal elements is significantly more difficult. This requires spin rotations on the target qubit, a process complicated by the interactions with neighbouring qubits. In order to rotate the target qubit, its nearest and next-nearest qubits should be neither in the Rydberg state nor resonant with the ground-Rydberg transition. To this end, we developed an approach in which we apply 480-nm Rydberg addressing lasers to the four neighbouring sites, as shown in Fig. 4a. The 480-nm lasers will first transfer the Rydberg populations in these neighbouring sites to the ground state via spontaneous emission from the intermediate state $\left|e\right\rangle$. Then, these addressing lasers create an electromagnetically induced transparency (EIT) condition, preventing the neighbouring sites from being excited by the ground-Rydberg excitation lasers. Finally, we apply the global excitation lasers to implement the spin rotations needed in state tomography onto the target qubit. Figure 4b(c) shows the experimentally measured (numerically simulated) spatio-temporal evolution of the Holevo information, which are in good agreement (Supplementary Fig. 22). We observe a clear linear light cone structure and a distinct *collapse-and-revival* pattern of ${{\mathbb{X}}}_{j}(t)$, differing from the OTOC results in Fig. 3j, l. Notably, during the time intervals when the ZZ-OTOC values of all qubits approach 1—indicating ineffective perturbations—we still observe non-uniform Holevo information values. In contrast to OTOCs, Holevo information allows for continuous tracking of quantum information dynamics.

We also mention that the observed *collapse-and-revival* of Holevo information are related, but not equivalent to the oscillation of the scar state wavefunction under Hamiltonian evolution^49,50^, because the ${\widehat{\sigma }}_{c}^{x}$ operation flips the central spin, and thus involves both the scarred subspace and the thermal eigenstate bath into the ${{\mathbb{X}}}_{j}(t)$ dynamics. We attribute the observed *collapse-and-revival* pattern to the general kinetically constrained spin rotations in the PXP model. As illustrated in Fig. 1a and Supplementary Information Section 5.2, the Rydberg blockade effect leads to retarded rotations of spins near the central flipped spin, which then propagate outwards and form the linear light cone structure. Within the light cone, spins continue their constrained rotations, and the distinguishability of the reduced density matrices ${\widehat{\rho }}_{j}(t)$ and ${\widehat{\rho }}_{j}^{{\prime} }(t)$ periodically vanishes and reappears, manifesting as the *collapse-and-revival* behaviour in the Holevo information.

Furthermore, our results reveal an information transport behaviour: the local information initially encoded at one site can be recovered at both the initial and other distant sites after the evolution under a many-body interacting Hamiltonian. These dynamics demonstrate persistent information backflow from the environment (surrounding qubits) to the system (qubits initially hosting encoded information), a hallmark of non-Markovian open quantum dynamics^74^. This non-Markovianity can be quantified using the Holevo information and the trace distance approach (see Supplementary Information Section 3.3 and Supplementary Fig. 12). The observed non-Markovian quantum dynamics underscore the system’s quantum memory effects for locally encoded information and its ability to transport the information across distant sites.

## Discussion

We observe the *collapse-and-revival* pattern of the quantum information encoded in a strongly-interacting Rydberg atom array, offering a different picture of how quantum information propagates under kinetic constraints. We particularly mention that such information scrambling dynamics observed in our experiment are distinct from those in generic chaotic or many-body localised systems. This work unveils an unexplored realm of quantum information dynamics for further exploration.

The underlying mechanism for the observed phenomena can be attributed to the kinetically constrained spin rotations induced by the Rydberg blockade effect. More specifically, due to the spin-flip in Holevo information dynamics, the periodic reassembly of quantum information emerges from dynamics involving both the scarred subspace and the thermal eigenstate bath. This distinguishes our observed *collapse-and-revival* dynamics from the previously reported oscillations of scar state wavefunctions^49,50^. Moreover, as our analysis in the Supplementary Information Section 5.3 reveals, the presence of scar states does not necessarily coincide with quantum information *collapse-and-revival*, highlighting the unique nature of our observations (Supplementary Fig. 24).

The experimental techniques developed in this work are indispensable for the precise measurement of constrained spin dynamics, OTOCs and Holevo information. Notably, we demonstrated a digital-analogue approach to effectively reverse the many-body evolution in Rydberg atom systems. The multi-transition, site-selective addressability of our platform allows us to achieve high-fidelity many-body state preparation, as well as perform full quantum state tomography, even in the presence of rotation constraints. The tight integration of these techniques enrich the toolbox for Rydberg atom quantum simulations and are poised for application in future experiments.

Our work opens up a range of exciting research directions and potential applications. For instance, one could investigate the propagation and interference dynamics caused by multiple butterfly operator perturbations, which will reveal collective behaviours arising from the interplay between kinetic constraints and quantum correlations in many-body systems^55^. While the magnitude and persistence of the *collapse-and-revival* effect observed here are currently limited by coherence, previous studies have suggested that techniques such as Floquet engineering can significantly extend coherence time and modify the interactions in Rydberg PXP systems^52,75^. Building on this, incorporating more single-site operations to introduce precise spatial modulation could enable controlled steering of quantum information propagation, which is dynamically protected by kinetic constraints. These advances hold promise for practical applications in near-term quantum technologies, including robust quantum memory, quantum state transfer protocols and quantum metrology in many-body interacting systems^76–79^.

## Methods

### Preparation of the defect-free atom array

**Fig. 5:**
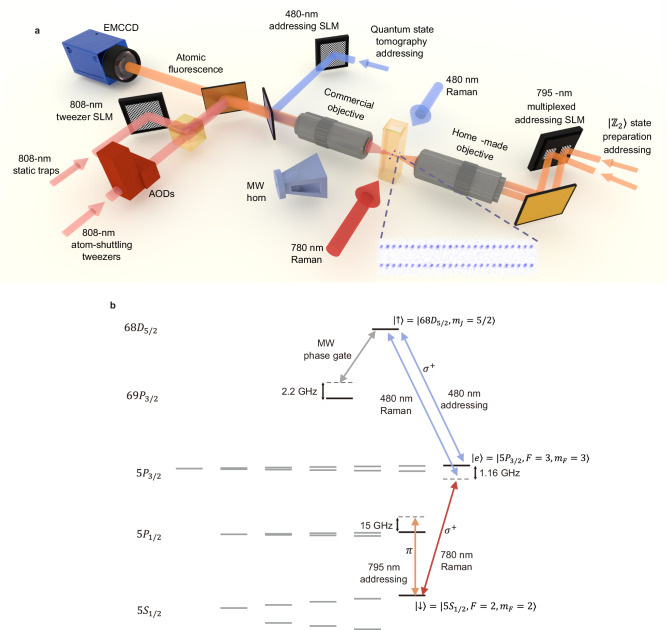
Experimental setup and atomic-level diagram. **a** Schematic of the essential elements in the experimental setup. The 808-nm static tweezer traps are generated by SLM, while the atom-shuttling tweezers are controlled by two acousto-optic deflectors (AODs). The 480-nm addressing laser beams are generated by another SLM. A commercial objective (G Plan Apo 50×, Mitutoyo, NA = 0.5) focuses the 808-nm tweezer beams and the 480-nm addressing beams, while collecting the 795-nm atomic fluorescence for imaging on an electron-multiplying CCD (EMCCD) camera. Counter-propagating 780 and 480-nm Raman beams enable coherent ground-Rydberg manipulation. The 795-nm addressing laser for local perturbation ${\widehat{\sigma }}_{c}^{z}$ and the laser array responsible for producing the alternating light shifts for $\left|{{\mathbb{Z}}}_{2}\right\rangle$ state preparation are targeted at separate areas of the same SLM. These distinct regions show different holograms, enabling varied addressing laser patterns. This spatial multiplexing technique allows for sub-microsecond switching between the $\left|{{\mathbb{Z}}}_{2}\right\rangle$ state preparation and local perturbation laser configurations. The 795-nm addressing laser beams are focused onto the atoms through a home-made objective (NA = 0.4). A microwave horn near the chamber generates MW pulses for Rydberg state manipulation (phase gate) and detection. The inset shows the atomic array. **b** Level diagram showing the atomic levels of ^87^Rb involved in the experiment. Our Rydberg excitation scheme employs a two-photon Raman transition from the ground state $\left|\downarrow \right\rangle=\left|5{S}_{1/2},F=2,{m}_{F}=2\right\rangle$ to the Rydberg state $\left|\uparrow \right\rangle=\left|68{D}_{5/2},{m}_{J}=5/2\right\rangle$. The transition is driven by a ${\widehat{\sigma }}^{+}$-polarised 780-nm laser, which couples the ground state to the intermediate state $\left|e\right\rangle=\left|5{P}_{3/2},F=3,{m}_{F}=3\right\rangle$, and a ${\widehat{\sigma }}^{+}$-polarised 480-nm laser, which connects the intermediate state to the Rydberg state. Both lasers are detuned from the intermediate state by Δ = 2*π* × 1.16 GHz. A microwave field, red-detuned by 2*π* × 2.2 GHz from the $\left|\uparrow \right\rangle$ to $\left|69{P}_{3/2},{m}_{J}=3/2\right\rangle$ transition, generates AC-Stark shifts on the Rydberg states and provides the phase gate for reversing the PXP Hamiltonian. For local single-qubit control, 795-nm addressing beams, blue-detuned by 2*π* × 15 GHz from the $\left|\downarrow \right\rangle$-$\left|5{P}_{1/2}\right\rangle$ transition, and 480-nm addressing beams, resonant with the $\left|\uparrow \right\rangle$-$\left|e\right\rangle$ transition, are employed.

In our experiment, atoms are loaded from a magneto-optical trap and captured by optical tweezers formed by focusing 808-nm laser beams diffracted through a spatial light modulator (SLM) (Supplementary Fig. 1). A measurement and feedback process eliminates defects in the atom chain, enabling the rapid creation of fully filled arrays (Fig. 5a).

### Experimental parameters

Once the defect-free array is prepared, all atoms are optically pumped into the ground state. The interatomic spacing is ~7 μm, resulting in nearest-neighbour and next-nearest-neighbour interactions of *V*_*i*,*i* + 1_ = 2*π* × 7.3 MHz and *V*_*i*,*i* + 2_ = 2*π* × 0.11 MHz, respectively. To closely approximate the PXP model despite the fixed ratio of 64 between the vdW interactions *V*_*i*,*i*+1_/*V*_*i*,*i* + 2_, the ground-Rydberg driving Rabi frequency *Ω* is set to *V*_*i*,*i* + 1_/6 = 2*π* × 1.21(1) MHz during the state evolution, striking a balance between the two interaction strengths. In addition, a small detuning Δ = 2*π* × 0.22(1) MHz is introduced to compensate for the residual next-nearest-neighbour interactions *V*_*i*,*i* + 2_. Under these experimental parameters, the experimental Rydberg Hamiltonian is well approximated by the PXP model, with numerical simulations showing negligible deviations (see Supplementary Information Section 2.2 and Supplementary Fig. 5 for details).

### High-fidelity $\left|{{\mathbb{Z}}}_{2}\right\rangle$ state preparation

The 795 and 480-nm addressing laser beams are generated via SLM diffraction and focused onto the atoms through high numerical aperture objectives, enabling selective addressing by adjusting the SLM phase pattern. The 795-nm addressing beams off-resonantly couple the $\left|5{S}_{1/2}\right\rangle$ ground state to the $\left|5{P}_{1/2}\right\rangle$ state (Fig. 5b), inducing a light shift on the addressed atoms. During $\left|{{\mathbb{Z}}}_{2}\right\rangle$ state preparation, the addressed atoms experience a light shift of 2*π* × 12.2(3) MHz, sufficient to shield the atom from Rydberg excitation. Combine with the high-fidelity global *π*-pulse, we achieved the high-fidelity preparation up to a 25-atom array (Fig. 2b).

### Out-of-time-ordered correlator evolution

A crucial step in measuring the OTOC is the implementation of time reversal for the many-body Hamiltonian. Leveraging the fact that our Rydberg Hamiltonian is well approximated by the PXP model, we developed a time-reversal protocol by exploiting the particle-hole symmetry of the PXP Hamiltonian. We employ a far-detuned 200 ns-long microwave field to implement a global ${\widehat{\sigma }}^{z}$ gate, and realise the forward-and-backward Hamiltonian evolution of the experimentally prepared $\left|{{\mathbb{Z}}}_{2}\right\rangle$ state, as shown in Fig. 3c. Between the forward and backward evolution process, we introduced a local ${\widehat{\sigma }}_{c}^{z}$ gate as the butterfly operator (see Supplementary Information Section 3.1 for details). To implement the local ${\widehat{\sigma }}_{c}^{z}$ gate, a light shift of approximately 2*π* × 5 MHz is applied to the central qubit, allowing a *π*-phase shift to be achieved within about 110 ns.

### Full quantum state tomography

To perform full quantum state tomography under the PXP kinetic constraints, we utilise 480-nm addressing lasers to transfer the Rydberg population of the four neighbouring atoms (as shown in Fig. 4a) surrounding the target qubit back to the ground state. The 480-nm addressing lasers are resonant with the $\left|e\right\rangle$-$\left|r\right\rangle$ transition, with a Rabi frequency of *Ω*_480_ ~ 2*π* × 20 MHz. These lasers enable rapid Rydberg-to-ground state transfer and create an EIT condition that prevents neighbouring qubits from participating in the ground-Rydberg transitions driven by the global excitation lasers.

### Numerical methods

In the numerical simulations presented in the main text and Supplementary Information, we employ exact diagonalization for atom chains with up to 13 atoms. For the chain with 25 atoms, we utilise the matrix product operator (MPO) method. Details of these approaches can be found in Supplementary Information Section 2.1: 'Effective Hamiltonian and numerical simulation methods'.

### Numerical results

**Fig. 6:**
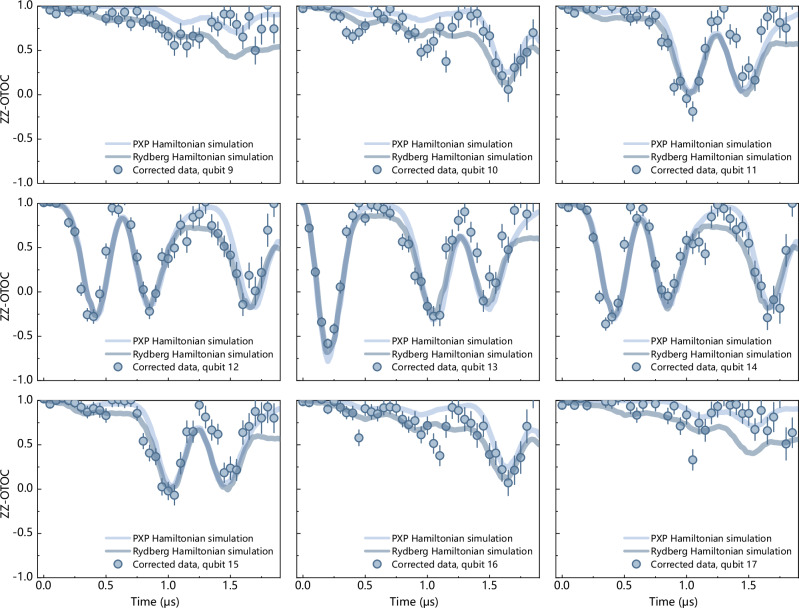
Mitigated ZZ-OTOC results for $\left|{{\mathbb{Z}}}_{2}\right\rangle$ state. Blue points represent mitigated experimental ZZ-OTOC data for the $\left|{{\mathbb{Z}}}_{2}\right\rangle$ state, corrected based on IZ-OTOC results. Light blue curves represent the simulated ZZ-OTOC dynamics under the PXP Hamiltonian [Eq. 1], accounting for experimental noise stemming from imperfections in the local perturbation ${\widehat{\sigma }}_{c}^{z}$. Dark blue curves show the simulated ZZ-OTOC dynamics under the Rydberg Hamiltonian [Eq. 2], also incorporating imperfections in local perturbation ${\widehat{\sigma }}_{c}^{z}$. The imperfections in the local perturbation make the OTOC patterns less distinct, which cannot be mitigated using IZ-OTOCs. Therefore, these imperfections are incorporated in the numerical simulations using Monte Carlo methods (see Supplementary Information Section 4). Error bars indicate the standard deviation given by the binomial distribution.

In all numerical simulations presented in the main text, we use the full Rydberg Hamiltonian ${\widehat{H}}_{{{{\rm{R}}}}}$ [Eq. 2] with parameters used in our experiments, including a Rabi frequency of Ω = 2*π* × 1.21 MHz, Rydberg interaction *V*_*i*,*j*_ = *V*_*i*,*i* + 1_/∣*i* − *j*∣^6^ where *V*_*i*,*i* + 1_ = 2*π* × 7.2 MHz, a small detuning of Δ = 2*π* × 0.22 MHz ≈ 2*V*_*i*,*i* + 2_. In the numerical results shown in Figs. 2e, g and 4c, we incorporate experimental noises and imperfections based on the characterisation of our system, as detailed in Supplementary Information Section 4: Error analysis and mitigation. In Fig. 3l, m, we utilise the ideal Rydberg Hamiltonian without experimental noises or imperfections to demonstrate our effective error mitigation method. In Fig. 6, we simulate both the PXP and Rydberg Hamiltonians, considering only the imperfections arising from local perturbations, which cannot be mitigated (see Supplementary Information Section 4.4 and Supplementary Figs. 18–21).

## Supplementary information

Supplementary Information

Transparent Peer Review File

## Data Availability

The data generated in this study have been deposited in the Zenodo database ^80^.

## Code availability

The code used for the numerical simulations in this study has been deposited in the Zenodo database ^80^.
