# Supplementary material for: Observation of quantum information collapse-and-revival in a strongly-interacting Rydberg atom array
Source: Nat Commun. 2026 May 29;17:6998. doi: 10.1038/s41467-026-73520-3 (PMC13392073; doi:10.1038/s41467-026-73520-3)
Supplement: Supplementary file 1 — Supplementary Information [file 41467_2026_73520_MOESM1_ESM.pdf]

Supplementary Information for: Observation of quantum information collapse-and-revival in a strongly-interacting Rydberg atom array

De-Sheng Xiang[1,*], Yao-Wen Zhang[1,*], Hao-Xiang Liu[1,*], Peng Zhou[1,*], Dong Yuan[2,*],Kuan Zhang[1,3,4,*], Shun-Yao Zhang[3], Biao Xu[1], Lu Liu[1], Yitong Li[1], and Lin Li[1,3,4,6,†]

[1]*National Gravitation Laboratory, School of Physics, Huazhong University of Science and Technology, Wuhan 430074, China*

[2]*Center for Quantum Information, IIIS, Tsinghua University, Beijing 100084, China*

[3]*MOE Key Laboratory of Fundamental Physical Quantities Measurement, Huazhong University of Science and Technology, Wuhan 430074, China*

[4]*Hubei Key Laboratory of Gravitation and Quantum Physics, Institute for Quantum Science and Engineering, School of Physics, Huazhong University of Science and Technology, Wuhan 430074, China*

[5]*Lingang Laboratory, Shanghai 200031, China*

[6]*Wuhan Institute of Quantum Technology, Wuhan 430074, China.*

[*] These authors contributed equally to this work.

[†] E-mail: li_lin@hust.edu.cn

CONTENTS

1. Experimental details 2
   1.1. Experimental setup 2
   1.2. Experiment timing sequence 3
   1.3. $|\mathbb{Z}_2\rangle$ state preparation and characterization 5

2. Theoretical modeling and numerical simulation 6
   2.1. Effective Hamiltonian and numerical simulation methods 6
   2.2. Parameter tuning for optimal quantum dynamics 9
   2.3. Boundary and finite-size effects 9

3. Probing quantum information scrambling and transport dynamics 11
   3.1. OTOC measurements details 11
   3.2. Holevo information measurements details 13
   3.3. Non-Markovian quantum information dynamics in a strongly-interacting Rydberg atom array 17

4. Error Analysis and Mitigation 18
   4.1. Initial state preparation error 18
   4.2. Detection and evolution error 20
   4.3. Error characterization 23
   4.4. Error mitigation for ZZ-OTOC 25
   4.5. Error mitigation for Holevo information 28

5. Kinetically constrained dynamics and quantum information collapse-and-revival 29
   5.1. Investigation of kinetically constrained dynamics 29
   5.2. Illustration of quantum information collapse-and-revival in PXP model 31
   5.3. Distinguishing scar state oscillations and quantum information collapse-and-revival 31

Supplementary References 33

1. EXPERIMENTAL DETAILS

Programmable atomic arrays have emerged as a novel platform for quantum simulation and computation, offering exceptional control and scalability for exploring quantum many-body phenomena. In the past decade, significant progress has been made in this field, including the preparation of atomic array[1–11], efforts towards quantum computation[12–28], quantum optimization[18,26,29,30], quantum simulations[31–40], and quantum metrology[41,42]. This section presents the details of our experiment, including the experimental apparatus, timing sequence, and $|\mathbb{Z}_2\rangle$ state preparation and characterization.

1.1. Experimental setup

Our experimental platform is built around a dual-chamber vacuum system, consisting of a 2D magneto-optical trap (MOT) chamber and a science chamber. In the 2D-MOT chamber, an ^{87}Rb atom source (ampule) produces a diffuse atomic vapor. These atoms are cooled and confined by the magnetic fields and 780-nm lasers, $2\pi \times 30\,\mathrm{MHz}$ red-detuned from the $|5S_{1/2}, F = 2\rangle \to |5P_{3/2}, F = 3\rangle$ cycling transition. The pre-cooled atomic ensemble is then transferred through a differential pumping aperture into the science chamber by a 780-nm pushing beam. The science chamber is a custom-designed rectangular glass cell (Japan Cell) with large optical access. Ultra-high vacuum conditions inside the chamber are maintained by a non-evaporable getter pump (NEXTorr D 200-5, SAES), achieving a pressure well below 10^{-11} mbar. This low background pressure initially allowed for single-atom trapping lifetimes exceeding 10 minutes. However, due to the malfunction of an ion pump (SP-4, JJJvac) in the 2D-MOT chamber, the lifetime has since been reduced to approximately 90 seconds. Atoms are captured and cooled in the science chamber by a three-dimensional magneto-optical trap (3D-MOT) using three pairs of counter-propagating 780-nm laser beams, with a magnetic field gradient of $15\,\mathrm{G\,cm^{-1}}$. Each beam contains a cooling light red-detuned by $2\pi \times 24\,\mathrm{MHz}$ from the $|5S_{1/2}, F = 2\rangle \to |5P_{3/2}, F = 3\rangle$ cycling transition, and a repumping light resonant with the $|5S_{1/2}, F = 1\rangle \to |5P_{3/2}, F = 2\rangle$ transition.

Single atoms are trapped in a static two-dimensional optical tweezer array generated by an 808-nm laser (TA pro, Toptica) operating in free-running mode. The laser beam illuminates a phase-control spatial light modulator (SLM, HED 6010-NIR-080-C, Holoeye) loaded with a phase hologram generated via the weighted Gerchberg-Saxton (WGS) algorithm. Additionally, a system of atom-shuttling tweezers, utilizing the same 808-nm laser source as the static array but with orthogonal polarization, allows for precise atom rearrangement. The atom-shuttling tweezers are controlled by a pair of orthogonally oriented acousto-optic deflectors (AODs, DTSX-400-800.850, AA Opto-Electronic), driven by radio frequencies with independent arbitrary waveforms generated by a dual-channel arbitrary waveform generator (AWG, M4i.6631-x8, Spectrum).

A high numerical aperture objective (G Plan Apo 50×, Mitutoyo, NA = 0.5) focuses both the static and movable tweezers while also collecting atom fluorescence, which is directed to an electron-multiplying CCD (EMCCD, iXon Ultra 888, Andor) camera for detection. Additionally, a 480-nm beam for local Rydberg control, utilizing a similar SLM-based approach, is focused through the same objective (see Fig. 5a). This shared configuration for trapping, addressing, and fluorescence detection enhances the stability of the system. Opposite the Mitutoyo objective is a home-made objective with a numerical aperture of 0.4. A CCD camera images the static tweezer array, consisting of 36 × 2 tweezers with 7 µm spacing and a beam waist of approximately 0.9 µm. Through iterative feedback and adjustments, the intensity variation across the entire array is kept well below 1%. The 795-nm addressing laser beams, essential for $|\mathbb{Z}_2\rangle$ state preparation and local perturbation, are focused through the home-made objective. Two counter-propagating laser beams—one at 780 nm and the other at 480 nm—enable global ground-Rydberg coherent manipulation. A microwave antenna, positioned near the science chamber, generates microwave pulses for Rydberg state manipulation and detection.

The experimental setup incorporates multiple laser systems for state preparation, qubit control, and detection. The 780-nm laser system, a tapered amplifier laser (TA Pro, Toptica), is used for both the MOT cooling beams and Raman light in the Rydberg excitation scheme. Its frequency is stabilized to an ultra-low expansion reference cavity (SLS) with a finesse of 26,000. An AWG drives a single-pass acousto-optic modulator (AOM, SGT200-780-0.5TA-B), optimized for modulation bandwidth, to dynamically adjust pulse frequency and intensity. For coupling the $|e\rangle \to |r\rangle$ transition, a 480-nm laser system (frequency-doubled TA-SHG Pro, Toptica) is employed, with the 960-nm seed laser locked to the same reference cavity as the 780-nm laser. An AOM driven by a direct digital synthesis (DDS, AD9910) controls the 480-nm laser beam, which is intensity-stabilized and remains on throughout the entire Rydberg operation sequence. Fast rising and falling edges of the Rydberg excitation lasers are achieved using electro-optic modulators (EOMs) for precise on-and-off switching.

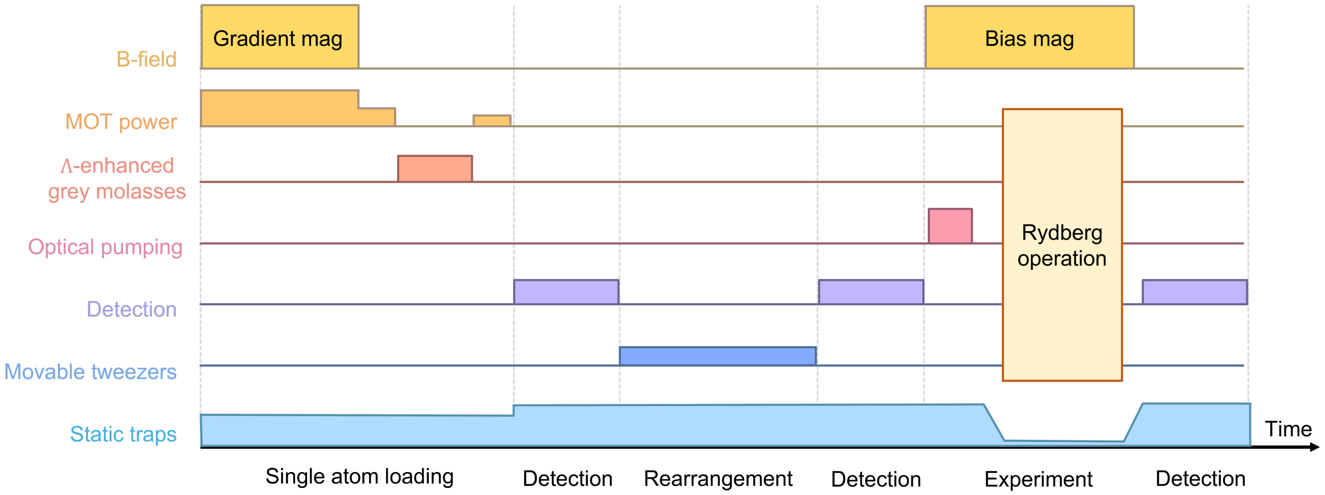

Supplementary Fig. 1. **Experimental timing sequence.** Summary of a typical experimental sequence as described in section 1.2. Detailed Rydberg experimental sequences for OTOC and Holevo information measurements are provided in Figs. 7,10.

1.2. Experiment timing sequence

The experimental sequence begins with the preparation of a cold atomic ensemble in the science chamber. A 3D MOT is loaded for 200 ms, producing an atomic cloud with a diameter of 500 µm and a temperature of 150 µK, measured via time-of-flight (TOF) expansion. To further reduce the temperature, polarization gradient cooling (PGC) is applied. This process involves quickly extinguishing the magnetic field gradient (within 500 µs) while increasing the cooling light detuning to approximately $2\pi \times 90$ MHz and reducing the intensity of both the cooling and repumping lasers. As a result, the atoms are cooled further to approximately 40 µK. The laser-cooled atomic ensemble serves as a reservoir for stochastic loading of the programmable atomic array. For stochastic loading, Λ-enhanced grey molasses (ΛGM) is implemented using two counter-propagating 795-nm laser beams. An additional stage of polarization gradient cooling (PGC), optimized for in-trap cooling, is then applied. This combined cooling approach results in a single-atom loading efficiency of approximately 80%, with an average atom temperature of 15 µK in traps with a depth of 1 mK, as measured using the release-and-recapture (R&R) method. Atom fluorescence detection utilizes the same 795-nm beams used for ΛGM, multiplexed after increasing the optical trap depth to 1.3 mK. By carefully balancing heating and cooling rates, a detection fidelity exceeding 99.9% is achieved with a 30 ms exposure time, while maintaining an average atom loss per detection below 1%. The use of 795-nm fluorescence imaging minimizes crosstalk from the strong 780-nm beams, ensuring accurate atom detection.

An atom rearrangement procedure generates a defect-free, one-dimensional atomic chain. The process begins by linearly ramping up the intensity of the movable tweezers from zero to three times the static trap depth, transferring atoms from the static SLM-generated traps to the movable tweezers. The AODs are then driven with time-dependent waveforms to transport the atoms at an average speed of 100 µm ms^{-1}, following a sinusoidal velocity profile to ensure smooth acceleration and deceleration. The rearrangement sequence is determined using a modified Hungarian algorithm[4], optimizing atom movement column by column. To minimize unnecessary atom loss and heating from moving tweezers sweeping through static traps, the tweezer paths include additional segments to bypass intervening traps. All AOD waveforms for the rearrangement process are pre-computed, allowing for rapid execution of the sequence. Post-rearrangement measurements show that atom temperatures increase to approximately 50 µK in the 1 mK deep traps.

For coherent Rydberg excitation, a two-photon Raman scheme is employed. A red-detuned 780-nm laser with σ^+ polarization couples the ground state to the intermediate state $|5P_{3/2}, F = 3, m_F = 3\rangle$ (see Fig. 5b). The collimated 780-nm laser is directed onto the atoms with a beam waist of approximately 300 µm and a maximum Rabi frequency of $\sim 2\pi \times 100$ MHz. Simultaneously, a blue-detuned 480-nm laser, also with σ^+ polarization, connects the intermediate state to the Rydberg state $|\uparrow\rangle = |68D_{5/2}, m_J = 5/2\rangle$. The 480-nm laser is focused onto the atoms with a beam waist of 13 µm, with a maximum Rabi frequency of $\sim 2\pi \times 70$ MHz. Both lasers are detuned from the intermediate state

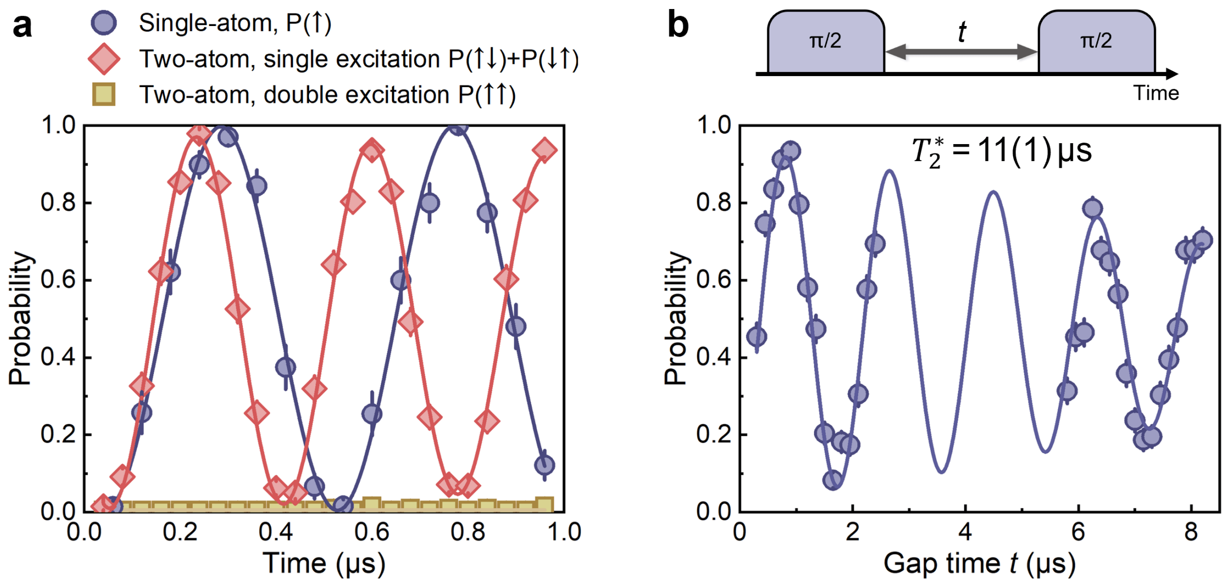

Supplementary Fig. 2. **Ground-Rydberg state Rabi oscillation, Rydberg blockade, and Ramsey coherence. a**, High-contrast ground-Rydberg state Rabi oscillation (blue circles) is observed when a single atom is driven by 480-nm and 780-nm Raman lasers. For a pair of atoms within the blockade radius, double Rydberg excitation (yellow squares) is strongly suppressed, and the Rabi frequency of the single excitation (red diamonds) is enhanced by a factor of $\sqrt{2}$ due to the Rydberg blockade effect. curves are damped sinusoidal fits. **b**, Ramsey oscillation showing Gaussian-type inhomogeneous dephasing with a coherence time of $T_2^* = 11(1)\,\mu\text{s}$. The measured probability represents $\frac{1}{2}(\langle\sigma^y\rangle + 1)$ after the gap time.

by $\Delta = 2\pi \times 1.16\,\text{GHz}$. A bias magnetic field of 30 G is applied throughout the experiment. Figure 2a (blue circles) illustrates the high-contrast ground-Rydberg state Rabi oscillation for a single atom, driven by 780-nm and 480-nm lasers. When two atoms are placed within the Rydberg blockade region, $\Omega \ll V$, double Rydberg excitations are strongly suppressed, as indicated by the yellow squares in Fig. 2a. Red diamonds in Fig. 2a shows that the Rabi oscillation between the Bell state $\frac{1}{\sqrt{2}}(|\downarrow\rangle|\uparrow\rangle + |\uparrow\rangle|\downarrow\rangle)$ and the ground state $|\downarrow\rangle|\downarrow\rangle$ is enhanced by a factor of $\sqrt{2}$. Figure 2b presents the ground-Rydberg coherence for a single atom, where we perform two $\pi/2$ ground-Rydberg rotations, separated by a variable gap, to implement a Ramsey sequence. The decay of the Ramsey oscillation fringe reveals a ground-Rydberg coherence time of $T_2^* = 11(1)\,\mu\text{s}$. The high-contrast Rabi oscillations and long coherence times achieved in our system lay a solid foundation for high-fidelity operations in subsequent experiments.

To perform local operations on the ground and Rydberg states, we employ 795-nm and 480-nm addressing laser beams generated by SLMs. The 795-nm laser beams are blue-detuned by $2\pi \times 15\,\text{GHz}$ from the $|5S_{1/2}, F = 2\rangle \to |5P_{1/2}\rangle$ transition, providing a light shift of $2\pi \times 12.2(3)\,\text{MHz}$. This enables the creation of an alternating pattern of excitable and non-excitable atoms, allowing the system to be prepared in a $\mathbb{Z}_2$-ordered configuration. Simultaneous imaging of the 795-nm atomic fluorescence and the 795-nm addressing laser beams on the EMCCD ensures precise spatial overlap between the lasers and the atoms. Measurements indicate that crosstalk to neighbouring atoms in the chain is suppressed to less than $2\pi \times 1\,\text{kHz}$. The 480-nm laser beams, resonant with the $|r\rangle - |e\rangle$ transition, are used to enable selective Rydberg-to-ground state transfer and create EIT conditions in nearest-neighbour (NN) and next-nearest-neighbour (NNN) sites. The 480-nm laser beam alignment is optimized by maximizing Rydberg-to-ground state transfer efficiency on the target site while minimizing crosstalk on the neighbouring sites. After optimization, more than 96% of the Rydberg state population in the target site is quickly transferred to the ground state, while the Rydberg state population in the neighbouring sites is reduced by less than 1%.

Finally, a state detection scheme is implemented to distinguish between ground and Rydberg states. Within 1 μs after the Rydberg operation, the trap depth is rapidly increased to 1.3 mK to expel Rydberg atoms from the trapping region while recapturing ground-state atoms. Following this, a strong 2.4 GHz microwave pulse is applied to deplete the remaining Rydberg populations using an RF power amplifier (HXPA7081G, HXINWB). This comprehensive detection scheme ensures that the probability of misidentifying a Rydberg atom as being in the ground state is less than 1%.

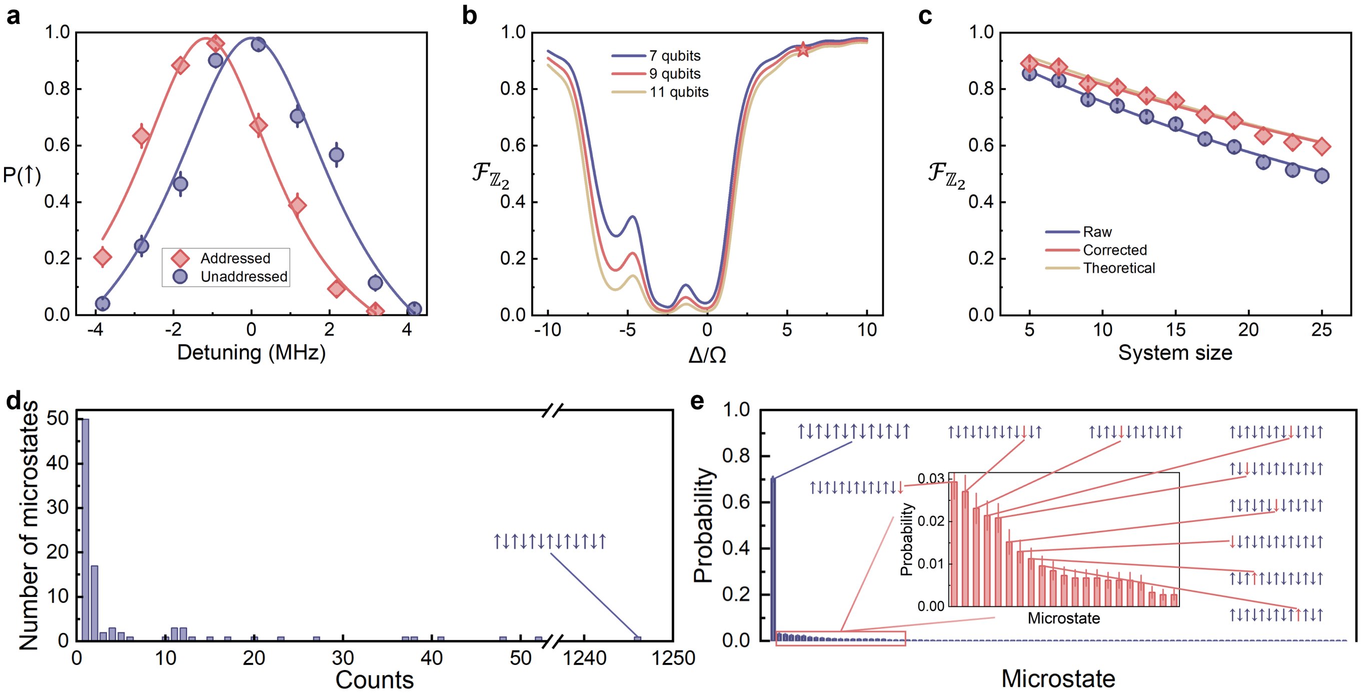

Supplementary Fig. 3. $|\mathbb{Z}_2\rangle$ **state preparation details.** **a**, Rydberg excitation spectra of addressed (red) and unaddressed (blue) atoms. The Rydberg population is shown as a function of Raman excitation laser detuning. **b**, Impact of the anti-blockade effect and addressing laser-induced light shift on state preparation fidelity. Simulated $|\mathbb{Z}_2\rangle$ state preparation fidelity as a function of 795-nm addressing laser light shift (in units of Rydberg Raman excitation Rabi frequency Ω) for various system sizes, with the nearest-neighbour Rydberg interaction strength $V_{i,i+1} = 3\Omega$. The red star marks our experimental condition. **c**, $|\mathbb{Z}_2\rangle$ state preparation fidelity as a function of system size. Red and blue lines: the exponential fit for corrected and uncorrected $|\mathbb{Z}_2\rangle$ state fidelity. Yellow line: theoretical upper limit for current experimental addressing approach. **d**, Number of 13-qubit microstates as a function of occurrences from 1,774 experiments. Successfully prepared $|\mathbb{Z}_2\rangle$ state: 1,246 counts (70% of events). **e**, Measured 13-qubit microstate distribution. Inset: dominant error states.

1.3. $|\mathbb{Z}_2\rangle$ state preparation and characterization

Rapid thermalization is ubiquitous in ergodic quantum many-body systems, leading to extensive research aimed at uncovering systems with fundamentally different dynamics[43–47]. Recently, studies involving Rydberg atom arrays have identified many-body scar states[31,48–53], which show weak breaking of ergodicity and maintain a degree of coherence over extended timescales. Similar phenomena have been observed in other systems such as superconducting processors[54] and Bose-Hubbard quantum simulators[55], and have triggered further theoretical research[56–60]. A previous theoretical work[61] has predicted that when the $\mathbb{Z}_2$-ordered Néel states are used as initial states for studying the spatial-temporal evolution of out-of-time-ordered correlators and Holevo information, one may observe persistent information backflow and an unusual breakdown of quantum chaos. These results suggest novel avenues for investigating unique dynamics of quantum information scrambling in kinetically constrained many-body systems. Here, we investigate quantum information scrambling using two initial states: (1) the $\mathbb{Z}_2$-ordered state $|\mathbb{Z}_2\rangle = |\uparrow\downarrow\uparrow\downarrow\uparrow\ldots\rangle$ and (2) the trivial product state $|\mathbf{0}\rangle = |\downarrow\downarrow\downarrow\downarrow\downarrow\ldots\rangle$. While $|\mathbf{0}\rangle$ can be readily prepared using optical pumping, achieving high-fidelity preparation of $|\mathbb{Z}_2\rangle$ state in large-scale systems remains challenging. Previous studies have successfully prepared $|\mathbb{Z}_2\rangle$ states in one-dimensional and two-dimensional atom arrays through adiabatic state transfer techniques[15,31,51], where the ground state of an engineered Hamiltonian is adiabatically transformed from $|\mathbf{0}\rangle$ to $|\mathbb{Z}_2\rangle$. However, as the system size increases, the exponential growth of the Hilbert space results in diminishing energy gaps, which causes a rapid decline of the state preparation fidelity.

Our experiment employs a scalable state preparation approach that combines global Rydberg excitation with site-selective laser addressing. A SLM is used to generate a customized light shift pattern across the atom array, selectively detuning certain sites from the ground-Rydberg transition (Fig. 3a), thereby creating an alternating arrangement of excitable and non-excitable atoms. The 795-nm addressing beams, $2\pi \times 15\,\mathrm{GHz}$ detuned from the D1 line resonance, induce an energy shift of $2\pi \times 12.2(3)\,\mathrm{MHz}$ on the ground state. The scattering rate caused by the 795-nm addressing beams is approximately $2\pi \times 5\,\mathrm{kHz}$, significantly lower than that caused by the Raman laser beams. Numerical

simulations (Fig. 3b) indicate that to maximize the fidelity of the $|\mathbb{Z}_2\rangle$ state, the sign of the light shift must be opposite to that of the Rydberg interaction, avoiding anti-blockade effects. Under our experimental conditions, numerical simulations yield a preparation infidelity of ~ 0.012 per qubit.

Our method remains highly effective as the system size scales up (Fig. 3c). The preparation infidelity is primarily attributed to uncorrelated single-qubit flip errors: 0.8% per qubit from Rydberg excitation inefficiency, 1.2% per qubit due to finite energy shifts, and 1% ($|\downarrow\rangle \rightarrow |\uparrow\rangle$) and 0.5% ($|\uparrow\rangle \rightarrow |\downarrow\rangle$) from state detection errors. This decomposition into single-qubit state preparations enhances the scalability of our approach compared to adiabatic transfer protocols. In an array of up to 25 atoms, we achieved the target $|\mathbb{Z}_2\rangle$ state with a measured fidelity of 49(3)%, which is corrected to 60(3)% after considering the detection errors. This high-fidelity preparation, in a Hilbert space of dimension 2^{25}, underscores the robustness and scalability of our protocol.

Microstate distribution analysis of the experimentally prepared $|\mathbb{Z}_2\rangle$ state reveals non-Poissonian error occurrences (Fig. 3d). Figure 3e shows the measured microstates distribution, highlighting that the dominant errors are single-qubit flips from $|\uparrow\rangle$ to $|\downarrow\rangle$, further confirming that our approach employs single-qubit operations to prepare a many-body state. This predictable error distribution facilitates error mitigation in the experimentally measured data for quantum information scrambling in $|\mathbb{Z}_2\rangle$ state, as discussed in section 4.

Next, the evolution and lifetime of the $|\mathbb{Z}_2\rangle$ state were investigated. The dynamics of the $|\mathbb{Z}_2\rangle$ state were measured under both forward-and-backward evolution (e^{-iHt} followed by e^{iHt}) and forward-only evolution (e^{-iHt}) (Fig. 4) using the PXP Hamiltonian $H = \sum_i P_i \sigma^x_{i+1} P_{i+2}$[48,62,63]. To characterize the evolution, the Rydberg state population $P(\uparrow)$ (Fig. 4a,b) and the average domain-wall density (Fig. 4c,d) are measured. Exponential fits to the data in Fig. 4a,c show the decay rate of the $|\mathbb{Z}_2\rangle$ state under forward-backward evolution, with a $1/e$ lifetime of approximately 1.6(1) µs for population, and 1.0(3) µs for average domain-wall density. This decay limits the contrast in the raw ZZ-OTOC data presented in Fig. 3f of the main text. Forward-only evolution of the average domain-wall density (Fig. 4d) is fitted to a damped sinusoidal function, yielding a $|\mathbb{Z}_2\rangle$ state lifetime of approximately 1.5(1) µs. The Rydberg population dynamics (Fig. 4b) are fitted using a damped Fourier series, yielding a $1/e$ decay time of approximately 2.8(2) µs. Additionally, in Section 4, the data from Fig. 4b are fitted to the error model to characterize the noise in the driving fields. The finite lifetime of the $|\mathbb{Z}_2\rangle$ state results in an overall loss during transport, as reflected in the global decay of the measured Holevo information in Fig. 4b of the main text. The breakdown of the defect-free $\mathbb{Z}_2$-ordered system into subsystems, indicated by the decay of domain-wall density (Fig. 4c) and the reduction in oscillation contrast (Fig. 4d), smears the dynamics and impedes quantum information propagation. While the state preparation errors can be easily corrected, the mitigation of errors during the evolution is far more complex. The above-detailed characterization of the prepared $|\mathbb{Z}_2\rangle$ state, particularly the decay rates during driven evolution, provides insights into the underlying noise sources (see section 4 for details on the noise model) and informs subsequent error mitigation strategies.

In summary, a scalable approach for preparing $|\mathbb{Z}_2\rangle$ states in large atomic arrays has been demonstrated. By combining global Rydberg excitation with site-selective addressing, high-fidelity state preparation was achieved in systems of up to 25 qubits. Detailed characterization of the state evolution provides valuable understanding of quantum scar dynamics and systematic noise sources. These findings lay the groundwork for further exploration of quantum information scrambling in scarred systems.

2. THEORETICAL MODELING AND NUMERICAL SIMULATION

2.1. Effective Hamiltonian and numerical simulation methods

Our experimental setup consists of a linear array of 25 individual atoms trapped in optical tweezers. The system dynamics are governed by the microscopic Hamiltonian:

$$H = \sum_i \left[\frac{\Omega}{2} \sigma^x_i - \Delta n_i \right] + \sum_{i<j} V_{ij} n_i n_j, \tag{S1}$$

where Ω is the Rabi frequency, Δ is the detuning, and $n_j = (1 + \sigma^z_j)/2$ is the projector onto $|\uparrow\rangle$ at site j, indicating whether the atom is in the Rydberg state. The interaction term $V_{ij} = C_6/R^6_{ij}$ represents the van der Waals interaction between atoms in $|\uparrow\rangle$ state at sites i and j, with C_6 being the van der Waals coefficient and R_{ij} the distance between atoms.

In the regime of strong nearest-neighbour interactions ($\Omega \ll V_{i,i+1}$), neglecting longer-range interactions ($V_{i,j>i+1}$), and setting the detuning $\Delta = 0$, the Hamiltonian simplifies to the effective PXP Hamiltonian via the Schrieffer-Wolff transformation:

$$H_{\mathrm{PXP}} = \sum_i P_i \sigma^x_{i+1} P_{i+2}, \tag{S2}$$

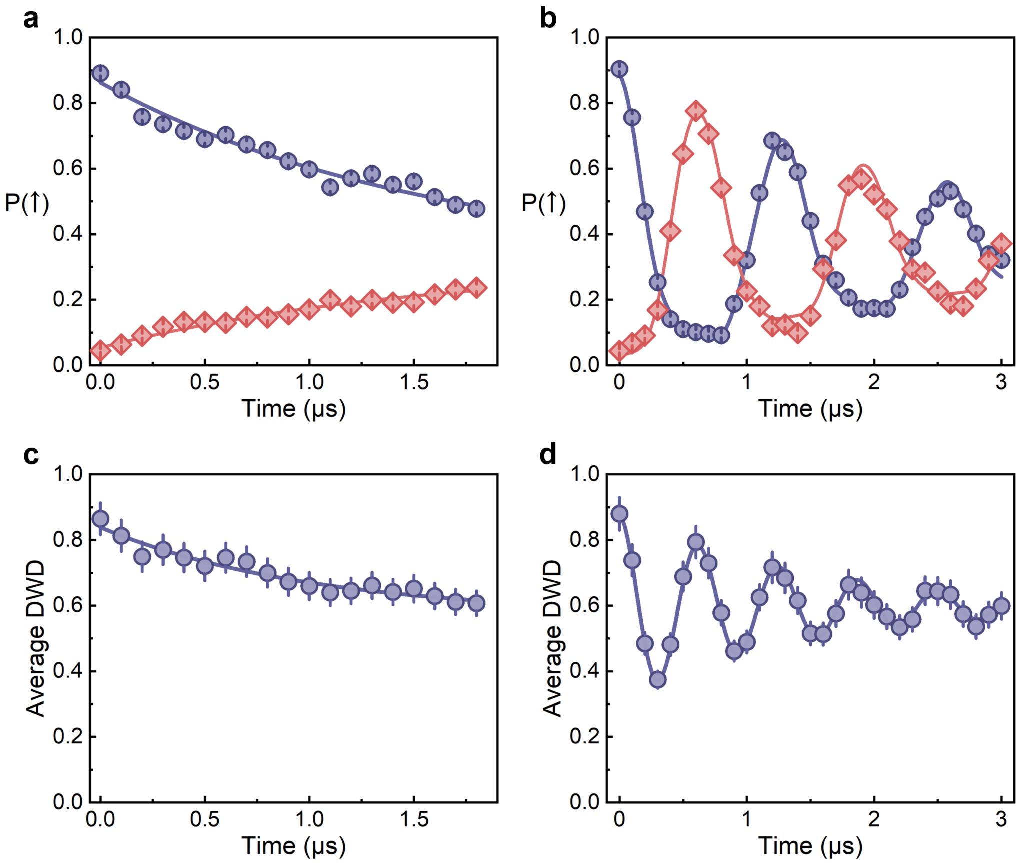

Supplementary Fig. 4. $|\mathbb{Z}_2\rangle$ **state dynamics under PXP Hamiltonian evolution.** **a** and **b**, Population of $|\uparrow\rangle$ (blue) and $|\downarrow\rangle$ (red) initialized qubits during **a**, forward-and-backward evolution (e^{-iHt} followed by e^{iHt}) and **b**, forward-only (e^{-iHt}) evolution. Curves in **a** represent exponential fits, while **b** is fitted with damped Fourier series up to the 5th order. **c** and **d**, Average domain-wall density (DWD) of the central 13 qubits during **c**, forward-and-backward evolution, and **d**, forward-only evolution. The blue curve in **c** is an exponential fit, while **d** is fitted with a damped sinusoidal function.

where $P_i = (1 - \sigma_i^z)/2$ is the projector onto the $|\downarrow\rangle$ state at site i, and $\sigma_i^{x,y,z}$ are Pauli matrices for the i-th qubit. The local three-body terms $P_i \sigma_{i+1}^x P_{i+2}$ impose kinetic constraints, allowing a Rydberg atom state to flip only if both neighbouring atoms are in the spin-down $|\downarrow\rangle$ state. This constraint effectively rules out configurations $|\cdots \uparrow_i \uparrow_{i+1} \cdots\rangle$ from the computational bases, as adjacent Rydberg excitations are forbidden by the blockade effect. The low-energy subspace, spanned by configurations without adjacent excited states, can be described by the projector:

$$\mathcal{P} = \prod_j (1 - n_j n_{j+1}) . \tag{S3}$$

This constrained subspace forms the effective Hilbert space of reduced dimensionality, governing the system's constrained dynamics. Remarkably, the dimension of this effective Hilbert space grows according to the Fibonacci sequence, scaling as ϕ^N, where $\phi = (1+\sqrt{5})/2$ is the golden ratio and N is the system size. This reduced dimensionality reflects the exclusion of certain configurations due to the kinetic constraints.

The PXP model exhibits three notable symmetries: (1) Discrete spatial inversion symmetry $\mathcal{I}$, mapping $j \to N - j + 1$. (2) Translational symmetry, applicable under periodic boundary conditions. (3) Particle-hole symmetry, represented by $\mathcal{C} = \prod_j \sigma_j^z$, resulting in $\mathcal{C} H_{\mathrm{PXP}} \mathcal{C} = -H_{\mathrm{PXP}}$. The particle-hole symmetry plays a crucial role in reversing the Hamiltonian evolution $\exp(-iHt)$ in our experiment. However, this symmetry is only present in the PXP Hamiltonian H_{PXP}, not in the full Rydberg Hamiltonian H governing the experiment, which causes imperfections in time reversal.

When initialized in the $|\mathbb{Z}_2\rangle$ state, the Rydberg atom system exhibits wavefunction oscillations with a slow decay. This decay is partially due to state loss and decoherence during the experimental evolution, but also arises from imperfections in the many-body scars and a small overlap with thermal states in the underlying PXP model. To better understand the contribution of intrinsic dynamics to the observed decay, we performed numerical simulations

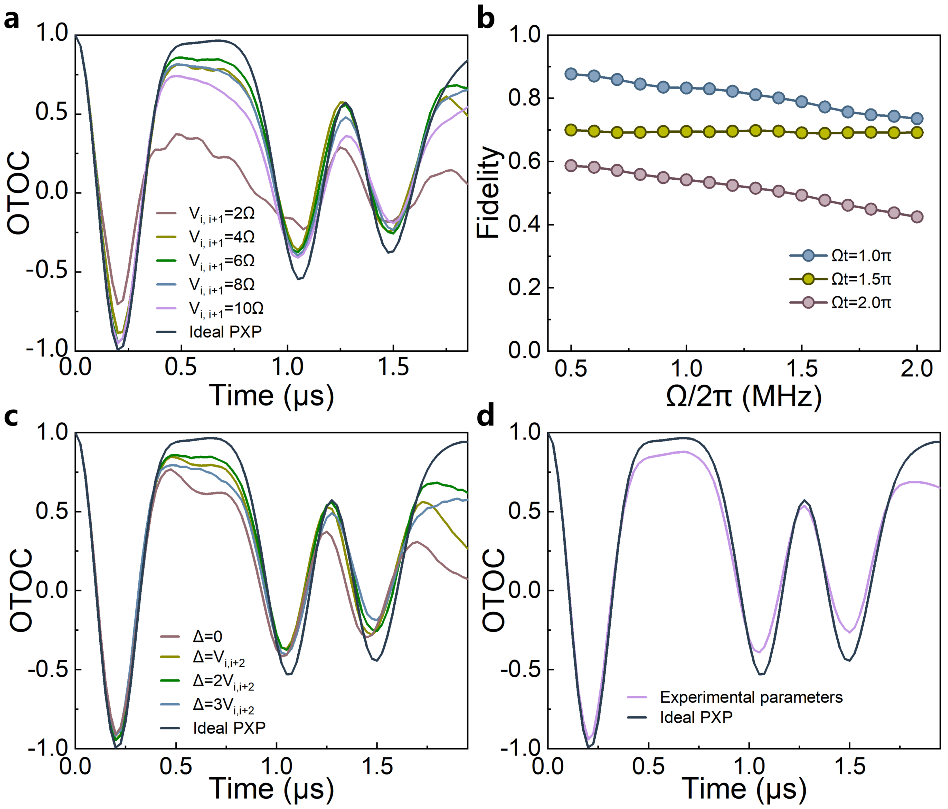

Supplementary Fig. 5. **Optimization of experimental parameters for closely approximating the ideal PXP Hamiltonian dynamics.** Numerical simulations of OTOC and time-reversal fidelity of the central qubit in a 10-qubit chain with periodic boundary conditions. **a**, Simulated OTOC dynamics for distinct nearest-neighbour (NN) Rydberg interactions $V_{i,i+1}$, in comparison to those of the ideal PXP model. The interaction strengths vary from 2Ω to 10Ω in steps of 2Ω. The optimal choice, $V_{i,i+1} = 6\Omega$, best matches the ideal case. **b**, Time-reversal fidelity as a function of Rabi frequency Ω, with $V_{i,i+1}$ fixed at 6Ω. Different time evolutions are shown for $\Omega t = 1.0\pi$ (blue symbols), $\Omega t = 1.5\pi$ (yellow symbols), and $\Omega t = 2.0\pi$ (red symbols). As Ω increases, the fidelity decreases. **c**, OTOC dynamics for different detuning values Δ, ranging from $\Delta = 0$ to $3V_{i,i+2}$, where $V_{i,i+2}$ is the next-nearest-neighbour (NNN) interaction. The black curve represents the ideal PXP model. The optimal detuning, $\Delta = 2V_{i,i+2}$, best matches the ideal dynamics. **d**, A comparison of OTOC dynamics using the optimized experimental parameters (violet curve) and the ideal PXP model (black curve), showing close agreement. The optimized parameters effectively reproduce the key oscillations observed in the OTOC.

of the revival behaviour, initializing the system of size $N = 25$ in the $|\mathbb{Z}_2\rangle$ state. The results, shown in Fig. 24c, provide evidence that a significant portion of the observed decay can be attributed to intrinsic features of the PXP model.

For all numerical simulations, we employ the Rydberg Hamiltonian (S1) to closely approximate the experimental physical conditions. For atomic chains of length ≤ 13, we utilize exact diagonalization to efficiently compute the full time evolution. However, for chains exceeding 13 atoms, exact diagonalization becomes computationally prohibitive due to exponentially increasing memory requirements and computation time, as well as limitations in available computational resources. Consequently, we implement the Matrix Product Operator (MPO) method to accelerate our numerical calculations in these cases. This approach allows us to extend our simulations to longer atomic chains while maintaining computational feasibility and numerical accuracy with TeNPy[64]. Unless otherwise specified, all simulations employ parameters identical to those in the experimental setup (detailed in Section 1). To account for experimental uncertainties, we implement a Monte Carlo method: accounting for fluctuations in Rabi frequency and detuning, laser noise, uncertainties in atomic positions and other relevant experimental parameters, all variables are randomly sampled from their respective probability distributions. Typically, we perform 200 runs and average the results. This comprehensive approach provides a robust representation of the system's behaviour under realistic experimental conditions.

2.2. Parameter tuning for optimal quantum dynamics

The approximation of the effective PXP Hamiltonian relies on two key assumptions: (1) Neglecting longer-range interactions $V_{i,j>i+1}$. (2) Ensuring that the nearest-neighbour interactions dominate over the Rabi frequency ($V_{i,i+1} \gg \Omega$).

However, in practice, the next-nearest-neighbour interactions $V_{i,i+2}$ cannot be ignored. To approximate the PXP model closely, the system must operate in the regime where $V_{i,i+2} \ll \Omega \ll V_{i,i+1}$. This poses a challenge because, in our 1D geometry with equally spaced atoms, the ratio $V_{i,i+1}/V_{i,i+2}$ is fixed at approximately 64. For example, setting $V_{i,i+1} \sim 16\Omega$ to meet the second assumption results in $\Omega \sim 4V_{i,i+2}$, making it difficult to fully suppress the effects of $V_{i,i+2}$. This creates a trade-off between the two key assumptions.

To find an optimal ratio of $V_{i,i+1}/\Omega$, we performed numerical simulations of the ZZ-OTOC with varying parameters. We identified the optimal ratio that minimizes the oscillation contrast decay, as shown in Fig. 5a. Based on these results, we set the experimental ratio of $V_{i,i+1}/\Omega$ to 6, which differs from the theoretical intermediate value of $\Omega = \sqrt{V_{i,i+1}V_{i,i+2}}$.

The selection of the Rabi frequency involves balancing two competing factors: (1) Time-reversal fidelity: Our simulations (Fig. 5b) show that increasing the Rabi frequency reduces time-reversal fidelity when $V_{i,i+1}/\Omega$ is fixed at 6 and the interval between forward and backward evolution is fixed at 200 ns. (2) Single-atom coherence: Higher Rabi frequencies improve single-atom coherence, which is mainly limited by laser phase noise.

After carefully weighing these factors, we chose a Rabi frequency of $\Omega = 2\pi \times 1.21(1)$ MHz. This value strikes a balance between maintaining adequate time-reversal fidelity and ensuring sufficient single-atom coherence for our experimental needs.

In addition to optimizing $V_{i,i+1}$ and Ω, we introduced a small detuning Δ to counteract the residual next-nearest-neighbour interactions $V_{i,i+2}$. Our simulations indicate that setting $\Delta = 2V_{i,i+2}$ best captures the OTOC dynamics (Fig. 5c).

In our OTOC measurement, a gap is inserted between the forward and backward evolutions, for the implementation of local and global single-qubit σ^z operations. To minimize the gap time to ~ 200 ns, we execute the local perturbations and global σ^z rotations in parallel, exploiting their commutative properties. We numerically investigated the impact of this finite gap on the OTOC dynamics observed in the experiment, as shown in Fig. 5d. Our analysis reveals that the 200 ns single-qubit operation time used in the experiment does not significantly affect the observation of the oscillation behaviour.

These parameter optimizations allow us to closely approximate the PXP model in our experimental setup (Fig. 5d), despite the inherent constraints of our system. These results highlight the importance of precise parameter tuning for accurately implementing the target Hamiltonian and maximizing the fidelity of quantum many-body scars' evolution in Rydberg atom systems. The optimized parameters facilitate the experimental realization of coherent spin rotation dynamics under kinetic constraints, closely approximating the ideal behaviour of the PXP model.

2.3. Boundary and finite-size effects

In finite-sized qubit chains, the existance of boundary qubits breaks the translational symmetry of the PXP Hamiltonian for certain initial states such as $|\mathbb{Z}_2\rangle = |... \uparrow\downarrow\uparrow\downarrow\uparrow ...\rangle$ and $|\mathbf{0}\rangle = |... \downarrow\downarrow\downarrow\downarrow\downarrow ...\rangle$. This symmetry breaking results in different interaction strengths between edge and bulk qubits (Fig. 6a), introducing significant boundary effects. Additionally, the finite number of particles leads to variations in the dynamical evolution of bulk atoms across different system sizes, introducing finite-size effects. For the Rydberg Hamiltonian, which includes long-range interactions, these boundary effects are further amplified due to differences in the residual interactions at the edges compared to the bulk.

To quantitatively analyse the boundary effect, we numerically simulate the evolution dynamics of both the central and the edge qubit in a 13-qubit chain, starting from the $|\mathbb{Z}_2\rangle$ state under the ideal Rydberg Hamiltonian. As shown in Fig. 6b,c, boundary effects cause the edge qubits to exhibit accelerated periodic oscillations. This acceleration arises from the reduced constraints on edge qubits, resulting in a stronger effective driving strength.

Moreover, both numerical simulations and experimental results reveal that boundary effects gradually alter the spin rotations from the outer edge inward, causing the initially uniform propagating wavefront to bend during the evolution of the $|\mathbb{Z}_2\rangle$ initial state, underscoring the critical role of boundary effects in shaping the system's dynamics.

Additionally, we investigate the impact of boundary effects on OTOC measurements by comparing the OTOC of the edge qubit and its neighbouring qubit in a 9-qubit chain with the corresponding qubits in a 25-qubit chain. As shown in the inset of Fig. 6d,e, significant differences are observed, highlighting the substantial influence of boundary effects on the OTOC dynamics. To ensure the accuracy of the observed OTOC dynamics over extended evolution

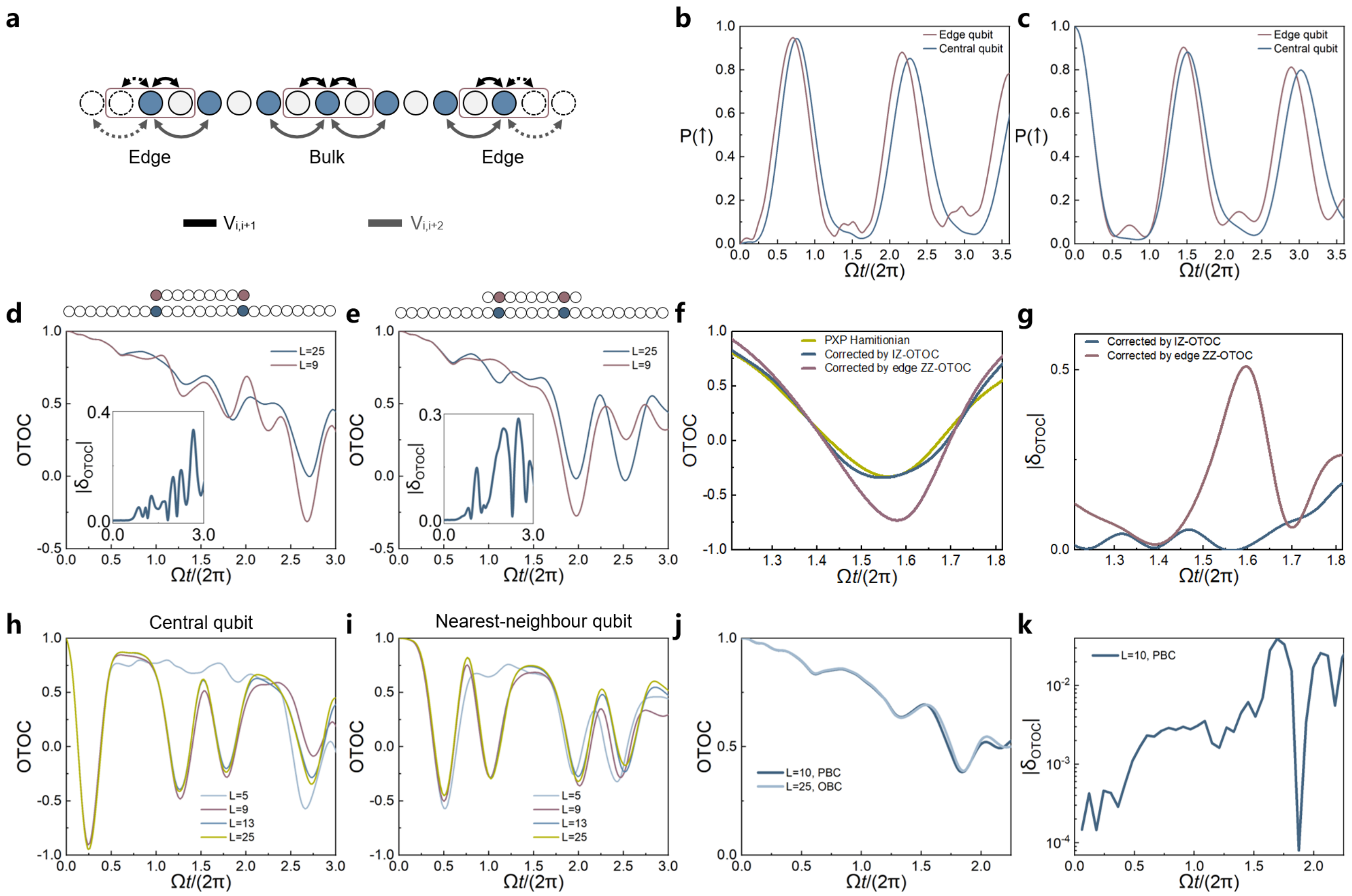

Supplementary Fig. 6. **Boundary and finite-size effects.** Simulated dynamics of OTOCs and time evolution under the ideal Rydberg Hamiltonian for various system sizes and boundary conditions, initialized in the $|\mathbb{Z}_2\rangle$ state. **a**, Schematic illustration of boundary effects in a 13-qubit chain. Boundary qubits lack nearest and next-nearest neighbours, breaking the translational symmetry. **b** and **c**, Numerical results of $\langle n_j(t)\rangle$ for edge (red) and central (blue) qubits in a 13-qubit chain, starting from the $|\downarrow\rangle$ state (**b**) and the $|\uparrow\rangle$ state (**c**). Accelerated oscillations of edge qubits reflect boundary effects. **d** and **e**, OTOC dynamics for the edge qubit (**d**) and its neighbouring qubit (**e**) in 9-qubit chain (red curves), compared to the corresponding qubits in 25-qubit chain (blue curves). The schematics above each plot, marked with coloured circles, illustrate the specific qubit positions within their respective chains. Insets show deviations $|\delta_{\text{OTOC}}|$ between the two dynamics, indicating significant boundary effects that propagate into the interior of the chain. **f**, OTOC evolution under the ideal PXP Hamiltonian (yellow), and under the noisy Rydberg Hamiltonian normalized using IZ-OTOC (blue) and the edge qubit's ZZ-OTOC (red). **g**, Deviations of normalized OTOCs from the ideal PXP case (red: edge ZZ-OTOC, blue: IZ-OTOC). Significant deviations observed in the case normalized using ZZ-OTOC from the edge qubit. **h** and **i**, Simulated OTOC dynamics for the central qubit (**h**) and nearest-neighbour (NN) qubit (**i**) in chains of various lengths (5, 9, 13, and 25 qubits) with open boundary conditions. Distinct variations of OTOC dynamics in smaller systems show the finite-size effects. **j** and **k**, Comparison of OTOC dynamics for qubits furthest from the perturbation in a 10-qubit chain (periodic boundary conditions, PBC, dark blue) and edge qubits in a 25-qubit chain (open boundary conditions, OBC, light blue). The negligible deviation $|\delta_{\text{OTOC}}|$ validates the use of a 10-qubit PBC system to approximate bulk qubit behaviour in a larger 25-qubit OBC system, matching our experimental conditions.

times, a larger system size is required.

Notably, as discussed in section 4.4, a reference OTOC measurement is required to serve as the normalization denominator for mitigating experimental imperfections. Theoretical analysis suggests that the IZ-OTOC is an appropriate reference for this purpose[65,66]. In our experiment, qubits near the boundary in the long chain remain unaffected by the local perturbation for a certain period, making them potentially suitable for approximating the IZ-OTOC dynamics for central qubits. To evaluate the effectiveness of different normalization schemes, we compared the edge qubit's ZZ-OTOC with the central qubits' IZ-OTOC as the normalization denominator in a noisy environment. Our numerical simulations indicate that using the edge qubit's ZZ-OTOC as the normalization denominator introduces over-corrections and temporal misalignment, as shown in Fig. 6f,g. These findings underscore the importance of employing the IZ-OTOC for accurate normalization and reliable mitigation.

We further study finite-size effects by comparing the OTOC dynamics of the central two qubits in chains of varying lengths (5-, 9-, 13-, and 25-qubit chains with open boundary conditions). Numerical simulations reveal that in smaller atomic systems, finite-size effects are very pronounced, as shown in Fig. 6h,i. This suggests that we need to extend the chain length to ensure that our experimental observations accurately reflect the scrambling of quantum information. However, simulating 25 atoms requires substantial computational resources, even when using the MPO method. To address this challenge, we simulate both a 25-qubit chain with open boundary conditions (OBC) and a 10-qubit chain with periodic boundary conditions (PBC), shown in Fig. 6j,k. We compare the OTOC dynamics of the atom furthest from the perturbation. The results reveal negligible differences within the experimental timescale, as the atom remains unaffected by the equivalent local perturbations at both ends during this period.

Based on this analysis, when simulating the dynamics of OTOC, we employ simulations of a 10-qubit chain with PBC. For experimental studies of quantum information scrambling within a constrained Hilbert space, we employ a 25-qubit chain to shield the central 13 atoms, thereby avoiding boundary effects and finite-size effects.

3. PROBING QUANTUM INFORMATION SCRAMBLING AND TRANSPORT DYNAMICS

Quantum information dynamics, the study of how local quantum information propagates in complex many-body systems, plays a crucial role in the understanding of many fundamental questions. It can be employed to study the limits on the speed of information propagation in quantum systems[67–83]. It is also deeply linked to quantum chaos and quantum thermodynamics[47,61,84–88], providing insights into dynamics in thermal and non-ergodic systems [89–101]. Moreover, in black hole physics, quantum information scrambling is related to the information paradox[102–106]. Additionally, quantum information dynamics has broad potential applications. In quantum computing, understanding information spreading is vital for developing noise-resistant systems and enhancing quantum error correction[66,107]; in quantum metrology, it could inspire novel precision measurement protocols[108]. In this section, we present the details of our study on quantum information scrambling and transport using a Rydberg atom array, including the measurements of out-of-time-ordered correlators, and Holevo information.

3.1. OTOC measurements details

Out-of-time-ordered correlators (OTOCs) have become a powerful tool for investigating quantum information scrambling, revealing how local perturbations propagate through quantum systems[47,95,96,98–100,109–114]. Experimental demonstrations of OTOC measurements have been achieved in several quantum platforms, including superconducting circuits[66,115–118], trapped ions[107,119–121], nuclear magnetic resonance (NMR)[122–127], nitrogen-vacancy (NV) centres[128], degenerate Fermi gases[129] and cavity quantum electrodynamics (cavity QED) system[108]. In this section, we provide a detailed description of the OTOC measurements.

Figure 7 illustrates the detailed pulse sequence for measuring the ZZ-OTOC. The measurement protocol begins with the preparation of two distinct initial states, $|\mathbb{Z}_2\rangle$ and $|\mathbf{0}\rangle$ (see section 1.3 for details on state preparations). The system then undergoes forward time evolution under the Hamiltonian H for a duration t. Next, a local perturbation σ_j^z is selectively applied to the central (13th) atom by inducing a π-phase shift with a 795-nm addressing laser. At the same time, a global $\prod_i \sigma_i^z$ rotation is performed on all qubits via a microwave field. This global rotation, combined with the subsequent Hamiltonian evolution, effectively implements the time-reversed Hamiltonian $-H$ for an equal duration t. This carefully designed sequence realizes the desired OTOC measurement, $F_{ij}(t) = \langle\psi| W_i^\dagger(t) V_j^\dagger W_i(t) V_j |\psi\rangle$.

To mitigate the differential AC-Stark shifts induced by the tweezer traps on the ground and Rydberg states, the traps are switched off before Rydberg excitation and turned back on after state evolution. Given the estimated atomic temperature of approximately 10 µK and the release and recapture time of 10 µs, the resulting atomic loss is estimated to be around 1%.

In the regime where $V_{i,i+2} \ll \Omega \ll V_{i,i+1}$, the Rydberg blockade effect introduces kinetic constraints, excluding configurations with adjacent qubits in the Rydberg state, $|\cdots \uparrow_i \uparrow_{i+1} \cdots\rangle$, from the computational basis. This kinetically constrained system is well approximated by the PXP model. However, due to the nature of van der Waals interactions, the ratio $V_{i,i+1}/V_{i,i+2}$ is fixed at approximately 64, making it difficult to fully separate these energy scales. The experimental parameters are carefully optimized, with Ω set to approximately $V_{i,i+1}/6$, as detailed in section 2.2. Additionally, a small non-zero detuning Δ between the Raman excitation lasers and the ground-Rydberg transition is introduced to mitigate the residual next-nearest-neighbour interactions. Numerical simulations (Fig. 8a) indicate that a detuning of $2V_{i,i+2} \sim 2\pi \times 0.2\,\mathrm{MHz}$ better preserves OTOC oscillations, closely approximating the expected PXP dynamics.

The 795-nm addressing laser used for local perturbation σ_j^z and the laser array for generating the alternating light shifts in $|\mathbb{Z}_2\rangle$ state preparation are both directed onto different regions of the same SLM. These two independent

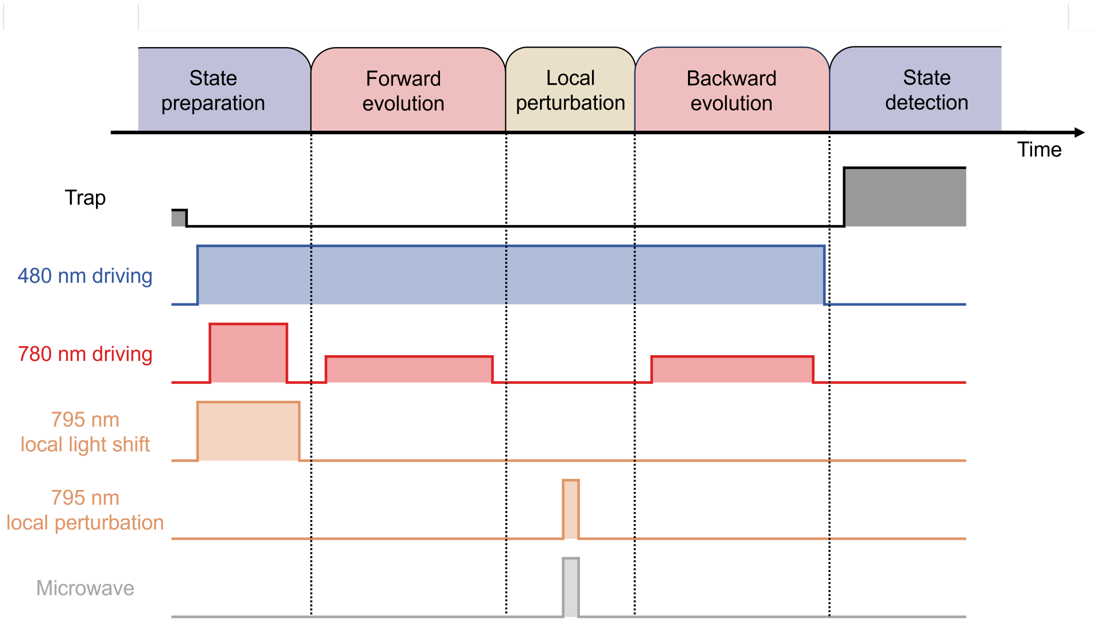

Supplementary Fig. 7. **Pulse sequence for OTOC measurements.**

regions display distinct holograms, allowing for different addressing laser patterns. This spatial multiplexing enables sub-microsecond switching of 795-nm addressing laser patterns between $|\mathbb{Z}_2\rangle$ state preparation and local perturbation, without being limited by the refresh rates of devices such as AODs (microsecond scale) and SLMs (millisecond scale). The duration of the local perturbation pulse is $\sim$110 ns, resulting in a π-phase shift on the ground states. The effectiveness of this local perturbation is experimentally verified by applying it to a single atom between two $\pi/2$ pulses separated by a fixed interval. By varying the phase of the second pulse, Ramsey-type oscillations of both addressed and unaddressed atoms are observed (Fig. 8b). The oscillations of the addressed atom are shifted by $1.01(2)\pi$ relative to those of the unaddressed atoms, confirming a controlled phase shift on individual atoms.

One of the key challenges in measuring OTOCs is implementing the inverse Hamiltonian evolution $\exp(-iHt)$ in a many-body system. In the Rydberg PXP model, we overcome this difficulty by exploiting particle-hole symmetry, represented by $\mathcal{C} = \prod_j \sigma_j^z$, which leads to the relation $\mathcal{C}H_{\text{PXP}}\mathcal{C} = -H_{\text{PXP}}$, effectively reversing the sign of the Hamiltonian. This symmetry allows us to implement the time-reversed Hamiltonian $(\prod_i \sigma_i^z)H_{\text{PXP}}(\prod_i \sigma_i^z) = -H_{\text{PXP}}$[130,131]. Experimentally, this is achieved by applying a global σ^z gate to all qubits using a $\sim$180 ns-long far-detuned microwave (MW) field. The MW field off-resonantly couples the Rydberg states $|\uparrow\rangle = |r\rangle = |68D_{5/2}, m_J = 5/2\rangle$ and $|r'\rangle = |69P_{3/2}\rangle$, and induces a π-phase shift on the state $|\uparrow\rangle$ via the AC Stark effect. This method enables the forward-and-backward evolution of the $|\mathbb{Z}_2\rangle$ state, with the measured results presented in Fig. 3c of the main text. Our digital-analogue approach offers an efficient and elegant way to implement time reversal in programmable Rydberg atom arrays, enabling precise measurements of quantum information scrambling via OTOCs. For the ZZ-OTOC measurement, we apply a local butterfly operator $W_i = \sigma_c^z$ to perturb the central qubit (the 13th qubit), while the measurement operator $V_j = \sigma_j^z$ acts on the j-th qubit. The ZZ-OTOC, denoted as $F_{ij}(t)$, where $i = c$ for the central qubit, can be expressed as:

$$F_{ij}(t) = F_{cj}(t) = \langle\Psi_0|\sigma_c^z(t)\sigma_j^z\sigma_c^z(t)\sigma_j^z|\Psi_0\rangle = \langle\Psi_0|\sigma_j^z|\Psi_0\rangle\langle\Psi_c(t)|\sigma_j^z|\Psi_c(t)\rangle, \tag{S4}$$

where $|\Psi_0\rangle$ is the initial state, and $|\Psi_c(t)\rangle = e^{iHt}\sigma_c^z e^{-iHt}|\Psi_0\rangle$ represents the time-evolved state after forward-and-backward Hamiltonian evolution over time t. For consistency, we fix $\langle\Psi_0|\sigma_j^z|\Psi_0\rangle = 1$. The expectation value $\langle\Psi_c(t)|\sigma_j^z|\Psi_c(t)\rangle$, which represents the correlation between the central qubit and the j-th qubit, can be directly obtained from site-resolved measurements of the Rydberg state population. Specifically, it is given by:

$$\langle\Psi_c(t)|\sigma_j^z|\Psi_c(t)\rangle = 2P_j(\uparrow) - 1, \tag{S5}$$

where $P_j(\uparrow)$ is the measured population of the $|\uparrow\rangle$ state for the j-th qubit in the array. Consequently, the ZZ-OTOC can be written as:

$$F_{cj}(t) = 2P_j(\uparrow) - 1. \tag{S6}$$

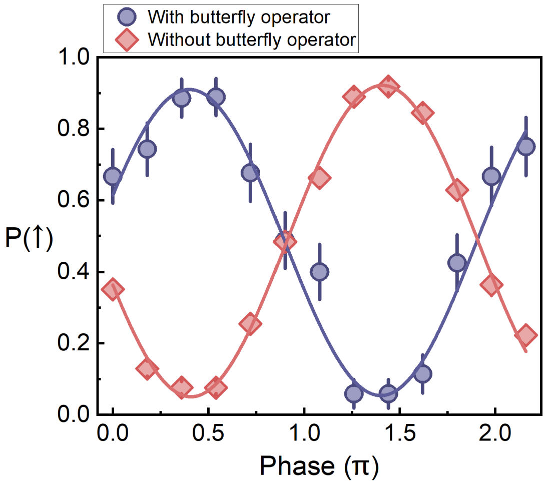

Supplementary Fig. 8. **Local perturbation.** Phase-dependent Ramsey oscillation of the central qubit, with (blue) and without (red) the local butterfly operator σ_c^z.

In the experiment, the Rydberg state population $P(\uparrow)$ is measured for each qubit after time evolution, allowing us to extract the full spatio-temporal dynamics of the OTOC across the atomic array.

To ensure consistency when measuring qubits in the $|\mathbb{Z}_2\rangle$ state, the measured j-th qubit is always initialized in the $|\uparrow\rangle$ state. Consequently, the indexing of the initial $|\mathbb{Z}_2\rangle$ state must be adjusted based on whether the measured qubit index j is odd or even. For the central 13 qubits, the initial $|\mathbb{Z}_2\rangle$ state is defined as $|\mathbb{Z}_2\rangle = |... \downarrow_{j-1}\uparrow_j\downarrow_{j+1} ...\rangle$, where the measured qubit is always initialized as $|\uparrow\rangle$, with the central qubit labeled as qubit 13. The data acquisition and processing procedure depends on the odd or even nature of the measured qubit index. For odd indices, $|\uparrow\rangle$-initialized qubit 13 is perturbed, and the OTOC data are collected from qubits 7, 9, ..., 19. For even indices, $|\downarrow\rangle$-initialized qubit 12 is perturbed, OTOC data is collected from qubits 7, 9, ..., 17, and aligned with qubit indices 8, 10, ..., 18 for consistency in indexing. Throughout the measurement process, the initial 25-qubit $|\mathbb{Z}_2\rangle$ state remains unchanged to maintain consistent initial state preparation fidelity.

To mitigate boundary effects inherent in finite-size systems (Fig. 6), the OTOC measurements focus on the central 13 qubits of the prepared 25-qubit $|\mathbb{Z}_2\rangle$ state. Boundary qubits experience fewer neighbouring interactions, which can lead to deviations in their dynamics. By concentrating on the central region, we limit the influence of these boundary effects and ensure that the measured dynamics more accurately represent the bulk behaviour of the PXP model. This strategy allows us to observe the intrinsic quantum information scrambling with higher fidelity, as the central qubits are less affected by edge-induced artifacts. Thus, the measured OTOCs from these qubits provide a better approximation of the expected PXP behaviour.

3.2. Holevo information measurements details

Holevo information, introduced by Alexander Holevo in 1973[132], is a fundamental concept in quantum information theory that sets an upper bound on the amount of information that can be reliably transmitted through a quantum channel[133,134]. It is formally defined as the difference between the von Neumann entropy of the average output state and the average of the von Neumann entropies of the individual output states. Mathematically, if $\rho_{\mathrm{X}} = \sum_i p_i \rho_i$ is the average output state corresponding to the quantum channel's output ensemble $\{p_i, \rho_i\}$, the Holevo information $\mathbb{X}$ is given by:

$$\mathbb{X} = S(\rho_{\mathrm{X}}) - \sum_i p_i S(\rho_i),$$

where $S(\rho)$ denotes the von Neumann entropy, defined as $S(\rho) = -\mathrm{Tr}(\rho \log_2 \rho)$. Importantly, Holevo information represents the upper bound on the accessible information that can be shared between two parties using a quantum channel, reflecting the best-case scenario for the amount of information that can be transmitted, regardless of the specific measurement strategy employed by the receiver.

To illustrate this, we consider an ensemble with two equally probable quantum states ρ and ρ'. Holevo information in this case can be interpreted as a measure of the distinguishability between the two states. Suppose Alice selects either

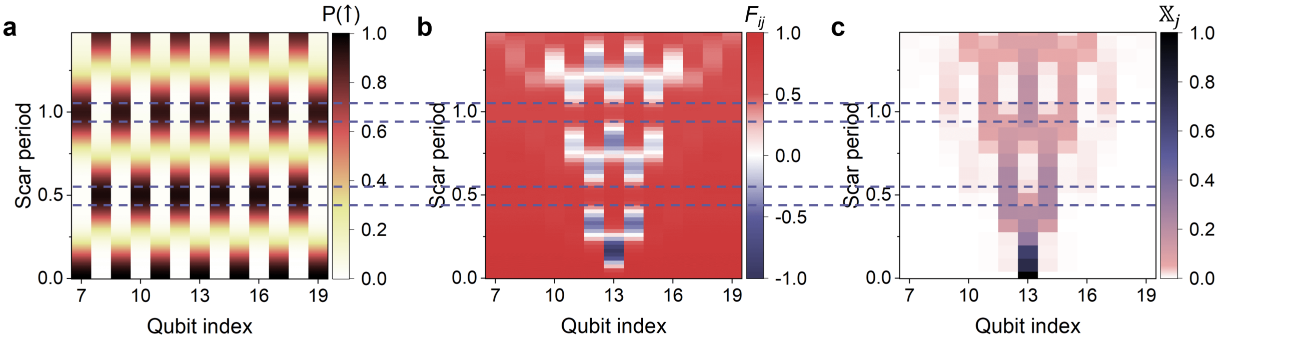

Supplementary Fig. 9. **Illustration of ineffective local perturbations.** Numerical simulations of **a**, $|\mathbb{Z}_2\rangle$ state evolution, **b**, ZZ-OTOC dynamics and **c**, Holevo information dynamics with the same time scale, mirroring Figs. 2e, 3l and 4c in the main text. Time intervals when all the qubits approach the $|\uparrow\rangle$ or $|\downarrow\rangle$ pure states are marked with two pairs of dashed lines (0.6 µs–0.7 µs and 1.2 µs–1.3 µs). During these intervals, the ZZ-OTOCs (F_{ij}) for all qubits approach 1 since the σ^z butterfly operator has no effect on the perturbed qubit. In contrast, Holevo information remains effective throughout, enabling uninterrupted tracking of quantum information dynamics.

ρ or ρ' with a probability of 1/2 and sends it to Bob through a quantum channel. Bob then performs a measurement to gain as much information as possible about which state Alice has sent. The information Bob retrieves from his measurement is upper bounded by the Holevo information $\mathbb{X}$. For instance, if ρ and ρ' are completely indistinguishable (i.e., $\rho = \rho'$), Bob cannot obtain any information about Alice's choice, and the Holevo information is zero ($\mathbb{X} = 0$). In this case, regardless of Bob's measurement, the outcome gives no clue about whether ρ or ρ' was sent. On the other hand, if ρ and ρ' are orthogonal, e.g. $\rho = |\uparrow\rangle\langle\uparrow|$ and $\rho' = |\downarrow\rangle\langle\downarrow|$, Bob can fully determine Alice's choice using an appropriate measurement, such as a σ^z measurement. In this case, the Holevo information reaches its maximum value of $\mathbb{X} = 1$, meaning Bob retrieves all the information about Alice's selection.

Holevo information has also been proposed as a powerful tool for studying scrambling and transport dynamics in many-body quantum systems[61,135,136]. Compared to OTOCs, which measure the scrambling of local perturbations in a system, Holevo information provides a different perspective on quantum information dynamics, as it does not rely on specific perturbations that may be ineffective under certain conditions.

In systems exhibiting quantum many-body scars, ZZ-OTOC measurements may occasionally become less informative due to the periodic oscillation of the scar state wavefunction. For example, during certain time intervals (such as 0.6 µs–0.7 µs and 1.2 µs–1.3 µs in Fig. 3j,l and Fig. 9), the ZZ-OTOC values for all qubits approach 1. The reason is that during these intervals, the wavefunction of the scar state $|\mathbb{Z}_2\rangle$ partially revives, causing the perturbed qubit to be mostly in either the $|\uparrow\rangle$ or $|\downarrow\rangle$ pure state, where the butterfly operator σ^z becomes ineffective. Consequently, no effective perturbation occurs, and no measurable scrambling is observed. As a result, ZZ-OTOC values of all qubits approach 1, due to the lack of an effective perturbation. In contrast, Holevo information can continuously capture quantum information dynamics, even during periods when the butterfly operator in ZZ-OTOCs become less effective (Fig. 9c).

This distinction is further emphasized when comparing Holevo information to classical Shannon information. While Shannon information only accounts for classical probability distributions, ignoring quantum phases, Holevo information captures both classical and quantum aspects of information, including coherence and entanglement. For example, between $|\leftarrow\rangle$ and $|\rightarrow\rangle$ states, Shannon information may be minimized to zero, while Holevo information can still be maximized, reflecting the quantum coherence between these states. In the specific context of the PXP model with the initial state $|\mathbb{Z}_2\rangle$, Holevo information reveals the retarded spin dynamics within the light-cone structure (see Fig. 23). This analysis highlights how kinetic constraints lead to a persistent phase delay in the propagation of spin rotations, influencing the overall information dynamics. Within this light cone, spins resume their constrained rotations; however, the distinguishability of spin states periodically collapses and revives, as captured by Holevo information. This behaviour reflects the constrained dynamics of the PXP model, where the Rydberg blockade effect induces delayed spin rotations near the central flipped spin, with this delay propagating outwards in a light-cone-like wavefront.

The light-cone behaviour has been theoretically studied in systems with power-law long-range interactions $(1/r^{\alpha})$[137–139]. The shape of the light cone in such systems depends on the power-law decaying exponent α and the spatial dimension d. When $\alpha > 2d + 1$, as in the case of the ideal PXP model and our experimental Rydberg Hamiltonian, Theoretical works[138,139] rigorously proved that the light cones are upper bounded by strictly linear light cones through the generalized Lieb-Robinson bound. In our work, the Rydberg atom array exhibits van der Waals interactions with $\alpha = 6$ in one dimension ($d = 1$), and the PXP model corresponds to the limit $\alpha \to \infty$ with $d = 1$, both falling into the regime where $\alpha > 2d + 1$, which ensures that quantum information propagates no faster than

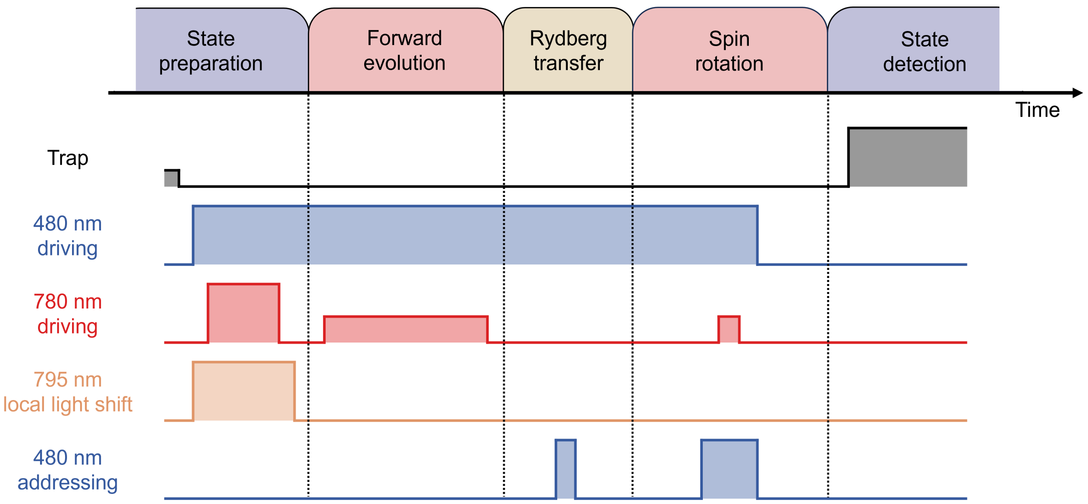

Supplementary Fig. 10. **Pulse sequence for Holevo information measurements.**

linear light cones. This theoretical upper bound aligns with the behaviour we observe experimentally in the Rydberg system, where a well-defined linear light cone is detected (see main text Figs. 3 and 4). Moreover, the propagation speed of information measured by Holevo information matches that of ZZ-OTOC. This consistency arises because both quantities characterize one-bit information scrambling dynamics in the same kinetically constrained system.

To understand how Holevo information applies to our experimental system, we consider the scenario where Alice and Bob transmit information through the $|\mathbb{Z}_2\rangle$ state. Based on the $|\mathbb{Z}_2\rangle$ state, Alice at the central site chooses whether to flip her qubit at $t = 0$, and Bob at site j measures his qubit at time t to infer Alice's choice. Outside the light cone, Bob retrieves no information ($\mathbb{X}_j(t) = 0$) due to the finite speed of information propagation. Inside the light cone, Bob periodically gains and loses information, as the distinguishability of spin states collapses and revives, leading to corresponding oscillations in Holevo information. This behaviour reflects the constrained dynamics of the PXP model, where the Rydberg blockade effect induces delayed spin rotations near the central flipped spin, with this delay propagating outwards. Notably, even when Bob measures the same qubit perturbed by Alice, the collapse-and-revival behaviour of quantum information can still occur due to the constrained dynamics in the system. Additionally, information can be retrieved from other sites, as the quantum information propagates across the system, even when Bob's qubit does not provide it.

Furthermore, Holevo information provides a robust measure of non-Markovianity, capturing the information backflow from surrounding spins to the central spin. Each spin exhibits periodic increases in Holevo information, signaling the backflow of quantum information, which is a clear signature of positive non-Markovianity[140–142]. This unique *collapse-and-revival* behaviour of Holevo information is distinct from the quantum scar state oscillations observed in similar systems, as it incorporates thermal eigenstates, thereby providing a broader picture of quantum dynamics beyond the scarred subspace.

To the best of our knowledge, this work presents the first experimental investigation of many-body dynamics in Rydberg atom array using Holevo information. In the rest of this section, we provide details regarding the experimental sequence and parameters utilized in our measurements of Holevo information.

Figure 10 illustrates the pulse sequence used to measure Holevo information dynamics, corresponding to Fig. 4a in the main text. The protocol begins with the preparation of two distinct initial states: $|\mathbb{Z}_2\rangle$ and $\sigma_c^x |\mathbb{Z}_2\rangle$, where σ_c^x acts on the central spin in the chain. These initial states evolve under the Rydberg Hamiltonian, after which quantum state tomography is performed to reconstruct the density matrix ρ_j for each qubit, enabling a detailed investigation of quantum information transport dynamics under kinetic constraints.

The Holevo information is extracted from the reconstructed density matrix, which includes both diagonal and off-diagonal elements. The diagonal elements of $\rho_j(t)$ and $\rho'_j(t)$ are obtained through projective measurement of σ_j^z on the j-th qubit, which can be accessed via Rydberg population measurement. However, measuring the off-diagonal elements requires a single-qubit $\pi/2$-rotation with a variable phase. This process is particularly challenging in

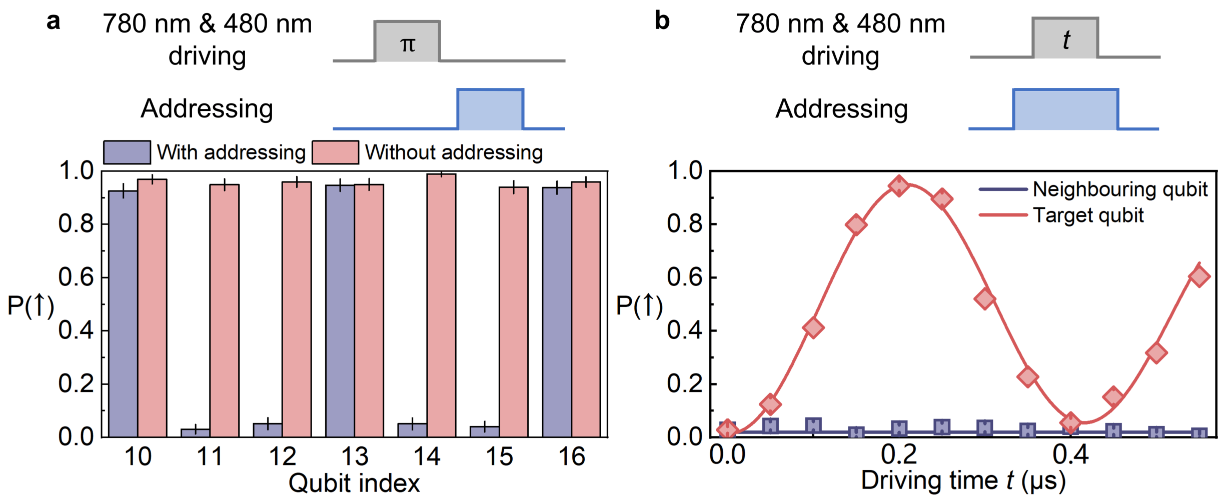

Supplementary Fig. 11. **Holevo information measurement details.** **a**, Rydberg population of the central 7 qubits, with (blue) and without (red) the Rydberg population transfer process. The data is obtained via Rydberg population measurement of even atoms and odd atoms, respectively. **b**, Under the driving of global 480-nm and 780-nm lasers, the target qubit (red diamonds) exhibits Rabi oscillation between the ground and Rydberg states, while the neighbouring qubits (blue squares), due to the EIT condition created by the 480-nm addressing lasers, do not participate in the spin rotation process.

strongly interacting Rydberg atom systems due to the constraints imposed by the PXP model, which requires nearest-neighbouring Rydberg atoms to be in the excitation blockade regime. The strong interactions between neighbouring Rydberg atoms create significant obstacles for performing spin rotations on any given qubit. If a nearest-neighbouring atom of the target qubit is in the Rydberg state, the blockade effect will prevent the target qubit from undergoing rotation. Even if only a next-nearest-neighbouring atom is in the Rydberg state, though not causing a full blockade, the residual Rydberg interaction will still affect the phase during quantum state tomography. Moreover, even when both nearest-neighbouring and next-nearest-neighbouring atoms are in the ground state, performing a spin rotation on the target qubit still remains difficult, as surrounding atoms can become entangled with the target qubit due to their involvement in the Rydberg excitation, resulting in a complex many-body quantum state. Therefore, to carry out the spin rotations required for quantum state tomography on the target qubit, it is necessary for the nearest-neighbouring and next-nearest-neighbouring atoms to neither be in the Rydberg state nor resonant with the ground-Rydberg transition.

Experimentally, we implement a novel approach for density matrix reconstruction using global rotations combined with 480-nm addressing lasers. The addressing beams, resonant with the transition from the excited state $|e\rangle$ to the Rydberg state $|r\rangle$, are selectively applied to the four neighbouring qubits, transferring their Rydberg populations to the ground state via spontaneous emission from the intermediate state $|e\rangle$. The decay rate of the Rydberg population during the transfer process, Γ_r, can be estimated by:

$$\Gamma_r = \Gamma_e \frac{\Omega_{480}^2/4}{\delta^2 + \Gamma^2/4 + \Omega_{480}^2/2}. \tag{S7}$$

Here, $\Gamma_e = 2\pi \times 6.06\,\text{MHz}$ is the natural linewidth of the excited state $|5P_{3/2}\rangle$. The terms Ω_{480} and δ denote the Rabi frequency and detuning of the addressing beams, respectively. This process lasts for approximately 200 ns, which is sufficient to deplete the Rydberg population at neighbouring sites (Fig. 11a), while being short enough to avoid the unwanted effects on the target qubit due to crosstalk from the 480-nm addressing lasers. After the state transfer process, the Rydberg population in the neighbouring qubits is reduced by 96(1)%, with negligible crosstalk-induced reduction in the Rydberg population of the target qubit.

Next, the 480-nm addressing lasers split the bare Rydberg state $|r\rangle$ into two dressed states: $|+\rangle = \frac{1}{\sqrt{2}}(|r\rangle + |e\rangle)$ and $|-\rangle = \frac{1}{\sqrt{2}}(|r\rangle - |e\rangle)$, separated by $\hbar\Omega_{480}$, where $\Omega_{480} \sim 2\pi \times 20\,\text{MHz}$ is the Rabi frequency of the 480-nm addressing laser. The dressed states are significantly detuned from the ground-Rydberg transition. The off-resonant excitation from the ground state $|g\rangle$ to the dressed states $|+\rangle$ and $|-\rangle$ causes destructive interference, preventing population transfer to the Rydberg state. This creates an electromagnetically induced transparency (EIT) condition, ensuring neighbouring qubits do not participate in the ground-Rydberg coherent driving implemented by the global excitation lasers.

Finally, single-qubit rotation on the target qubit is implemented using global excitation lasers. Figure 11b demonstrates that the target qubit can undergo single-qubit rotation (Rabi oscillation between the ground and Rydberg states) in the presence of neighbouring qubits addressed by the 480-nm laser. The addressed neighbouring qubits remain ineligible for the ground-Rydberg excitation process due to the EIT condition induced by the 480-nm laser.

In an ideal scenario with periodic boundary conditions, the PXP model would exhibit a stationary expectation value of the Pauli-X operator $\langle\sigma^x\rangle$ throughout the evolution. For initial states like $|\mathbb{Z}_2\rangle$ and $\sigma_c^x|\mathbb{Z}_2\rangle$, this implies that $\langle\sigma^x\rangle = 0$. However, during the gap time between the evolution and the projection measurement, the residual van der Waals interaction between the atoms could lead to the accumulation of phases, causing $\langle\sigma^x\rangle$ to become non-zero. This introduces challenges in accurately measuring the desired off-diagonal elements of the system's density matrix. Due to the difference in the initial states ($|\mathbb{Z}_2\rangle$ and $\sigma_c^x|\mathbb{Z}_2\rangle$), even after the same PXP evolution, the two output states may accumulate different residual phases. This phase difference introduces additional distinguishability when directly measuring the off-diagonal elements, increasing the difference between the two output states $\rho_j(t)$ and $\rho'_j(t)$. As a result, this extra distinguishability reduces the accuracy of the Holevo information. To address this, we measure the full parity oscillation curve by scanning the phase shift between two $\pi/2$ pulses. From this curve, we extract the oscillation amplitude A by fitting it to a sinusoidal function.

With the measured values of $P(\uparrow)$ and A for each qubit, we reconstruct the density matrix $\rho(t)$ at time t using the following expression:

$$\rho(t) = \frac{1}{2}(\mathbb{I} + (2P(\uparrow) - 1)\sigma^z) + \epsilon(t)A\sigma^y, \tag{S8}$$

where $\epsilon(t)$ represents the sign of $\langle\sigma^y\rangle$, with $\epsilon(t) = 1$ when $P(\uparrow)$ is expected to be increasing, and $\epsilon(t) = -1$ otherwise. Here, $\mathbb{I}$ is the identity matrix, and σ^y and σ^z are the Pauli-Y and Pauli-Z matrices, respectively.

The Holevo information for qubit j at time t can be obtained:

$$\mathbb{X}_j(t) = S\left(\frac{\rho_j(t) + \rho'_j(t)}{2}\right) - \frac{S(\rho_j(t)) + S(\rho'_j(t))}{2}, \tag{S9}$$

where $\rho_j(t)$ and $\rho'_j(t)$ are the density matrices of the j-th qubit evolved from distinct initial states $|\mathbb{Z}_2\rangle$ and $\sigma_c^x|\mathbb{Z}_2\rangle$. The von Neumann entropy $S(\rho) = -\mathrm{Tr}(\rho\log_2\rho)$ is used to quantify the information content of the density matrices.

The measurement of off-diagonal elements is what distinguishes quantum information from classical Shannon information. Shannon information, which only considers the diagonal elements of the density matrix, is a simple measure of classical probability distributions. In contrast, quantum information involves off-diagonal elements, which capture quantum effects like coherence and entanglement—features not present in classical systems. We emphasize that von Neumann entropy goes beyond Shannon entropy in quantum information science. While Shannon entropy only reflects the uncertainty in classical probability distributions, von Neumann entropy plays a central role in quantum systems. It is essential for quantifying information in quantum states and determining the capacities of quantum channels. More importantly, it captures quantum phenomena, such as entanglement, which are critical for understanding quantum systems. This is why we have made significant efforts to measure the off-diagonal elements, as they provide deeper insights into the unique aspects of quantum information.

3.3. Non-Markovian quantum information dynamics in a strongly-interacting Rydberg atom array

Non-Markovian quantum dynamics are often characterized by memory effects, where the system evolution depends on its past interactions with the environment. Unlike Markovian dynamics, where information is irreversibly lost to the environment, non-Markovian systems can experience information backflow, allowing for the recovery of previously lost information[143–146]. In many-body quantum systems, strong interactions could lead to non-Markovian dynamics, significantly influencing the spread and preservation of quantum information

In this work, we observe non-Markovian dynamics in a strongly interacting Rydberg atom array, focusing on the unusual behaviour of information backflow. We designate the central spin in an atomic array as the "system" and the surrounding spins as the "environment". The strong spin interactions allow information to transfer between the system and environment in complex ways, making it possible to observe non-Markovian effects.

Our experimental setup allows for precise quantum state tomography of each spin, enabling real-time tracking of information flow across the system. This capability provides detailed insight into non-Markovian behaviour by directly measuring how information, initially lost to the environment, returns to the spins that originally held it.

To quantify non-Markovianity, we assess the degree of information backflow using metrics like trace distance[147,148] and Holevo information[140–142], which track the evolution of quantum state distinguishability over time. The constrained spin rotations within the system drive this backflow, causing periodic collapses and revivals in information

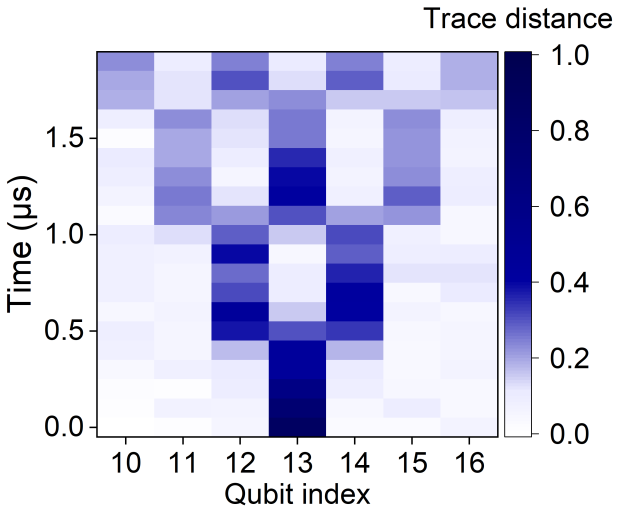

Supplementary Fig. 12. **Quantify non-Markovian dynamics via trace distance measurements.** Spatio-temporal dynamics of trace distance between $|\mathbb{Z}_2\rangle$ and $\sigma_c^x|\mathbb{Z}_2\rangle$ states under Rydberg Hamiltonian. Density matrices reconstructed from Holevo information measurements. The measurements reveal a linear light cone and *collapse-and-revival* pattern, demonstrating periodic increases in state distinguishability. This non-monotonic behaviour provides evidence of quantum information backflow, indicating non-Markovian dynamics in the Rydberg atom array.

distinguishability. This allows us to capture key signatures of non-Markovian dynamics as information spreads, collapses, and recovers between the system and environment. These findings offer valuable insights into quantum information dynamics and hold promise for advancing quantum memory technologies.

The trace distance is defined as

$$D(\rho_1, \rho_2) = \frac{1}{2}\mathrm{Tr}|\rho_1 - \rho_2|, \tag{S10}$$

where ρ_1 and ρ_2 are density matrices of $|\mathbb{Z}_2\rangle$ and $\sigma_c^x|\mathbb{Z}_2\rangle$, respectively. Figure 12 shows the experimentally measured spatio-temporal dynamics of the trace distance between the $|\mathbb{Z}_2\rangle$ state and $\sigma_c^x|\mathbb{Z}_2\rangle$ under the Rydberg Hamiltonian. Density matrices are reconstructed from Holevo information measurements. The plot reveals a clear linear light cone structure and a spatial-temporal *collapse-and-revival* pattern, mirroring observations in the Holevo information data presented in the main text. Trace distance, a widely used metric for quantifying non-Markovianity in quantum systems, tracks the distinguishability between two quantum states over time. While Markovian processes exhibit monotonic decay of trace distance, indicating irreversible information loss to the environment, our data shows periodic increases in trace distance. This observed pattern provides compelling evidence for quantum information backflow and the non-Markovian nature of the system's dynamics. These dynamics, marked by changes in state distinguishability, align well with theoretical expectations from the PXP model and display features of information backflow, where quantum information is periodically exchanged between the system and its environment rather than being permanently lost. Our findings are consistent with previous studies on non-Markovian behaviour in quantum systems[147].

4. ERROR ANALYSIS AND MITIGATION

In our Rydberg atom quantum simulator, imperfect Hamiltonian evolution and finite qubit coherence lead to the accumulation of both coherent and incoherent errors in the dynamics of OTOCs and Holevo information. This section analyses the major error sources, develops an error model to identify and characterize primary error mechanisms, and implements error mitigation techniques for ZZ-OTOC, enhancing the performance of the quantum simulator.

4.1. Initial state preparation error

As the number of atomic qubits increases, the exponential growth in the dimension of the density matrix amplifies the complexity of quantum state dynamics and the impact of initial state preparation errors. In studying ZZ-OTOC or Holevo information dynamics for $|\mathbb{Z}_2\rangle$ initial states within the constrained Hilbert space, rapid thermalization of

the error states could affect the behaviour of quantum information scrambling. Therefore, the fidelity of initial $|\mathbb{Z}_2\rangle$ state preparation is crucial for accurately probing quantum information dynamics in our system.

1. Influence on OTOCs

As mentioned in section 1.3, the $|\mathbb{Z}_2\rangle$ state preparation under global coherent Rabi excitation with site-selective addressing technique introduces errors primarily from infidelity in single-qubit operations. Our measurements of the microstate distribution revealed that errors in $|\mathbb{Z}_2\rangle$ state preparation are predominantly attributable to single qubit $|\uparrow\rangle \to |\downarrow\rangle$ flips. These error states account for 86(2)% of all occurrences in conjunction with the $|\mathbb{Z}_2\rangle$ state (Fig. 3d,e). The remaining error contribution primarily stems from detection errors (approximately 1% per qubit). Consequently, the prepared density matrix can be expressed as a weighted sum of the target $|\mathbb{Z}_2\rangle$ state and the major error states. Given the $|\mathbb{Z}_2\rangle$ state preparation fidelity of $\mathcal{F}_{\mathbb{Z}_2}$, the measurement results of ZZ-OTOC $F_{ij}^{exp}(t)$ can be therefore expressed as:

$$F_{ij}^{exp}(t) = \mathcal{F}_{\mathbb{Z}_2} \cdot F_{ij}^{\mathbb{Z}_2}(t) + \sum_{\alpha} \rho_{\alpha\alpha}(0) F_{ij}^{\alpha}(t) \tag{S11}$$

Here, $F_{ij}^{\mathbb{Z}_2}(t)$ represents the ZZ-OTOC dynamics with the ideal initial $|\mathbb{Z}_2\rangle$ state, and $F_{ij}^{\alpha}(t)$ denotes the dynamics for the α-th error state in the prepared density matrix $\rho(0) = \mathcal{F}_{\mathbb{Z}_2} |\mathbb{Z}_2\rangle \langle\mathbb{Z}_2| + \rho_{\alpha\alpha}(0) |\alpha\rangle \langle\alpha|$. To investigate the impact of $|\mathbb{Z}_2\rangle$ state preparation fidelity on ZZ-OTOCs, we simulated the ZZ-OTOC dynamics with various $|\mathbb{Z}_2\rangle$ state preparation fidelity (ranging from 0.2 to 1.0), as shown in Fig. 13. The atomic array in our experiment exhibits symmetry around the central qubit, which means that the dynamics of qubits at symmetrically equivalent positions are exactly the same. Taking advantage of this symmetry, we can fully characterize the system's behaviour by examining a subset of qubits. We present simulation results for four qubits that exhibit non-trivial dynamics and represent the distinct behaviours observed in the array. For these error states, we considered uniformly distributed $|\uparrow\rangle \to |\downarrow\rangle$ errors. This error state distribution aligns with the typical experimental conditions as characterized in section 1.3. The simulations reveal that the $|\mathbb{Z}_2\rangle$ state preparation fidelity significantly affects the contrast of the *collapse-and-revival* pattern within the light cone. Moreover, given our high $|\mathbb{Z}_2\rangle$ state preparation fidelity (78(1)% for 13-qubit chain, after detection error correction.) and the measured microstate distribution, numerical simulation results (Fig. 3b of the main text for qubit 13, and Fig. 14 for other qubits) demonstrate that the characteristic *collapse-and-revival* pattern remains clearly observable.

2. Influence on Holevo information

For Holevo Information, we consider the evolution of both the $|\mathbb{Z}_2\rangle$ and the $\sigma_c^x |\mathbb{Z}_2\rangle$ states, accounting for imperfections in state preparation. With the prepared density matrices $\rho(0) = \mathcal{F}_{\mathbb{Z}_2} \cdot |\mathbb{Z}_2\rangle \langle\mathbb{Z}_2| + \sum_\alpha \rho_{\alpha\alpha}(0) |\alpha\rangle \langle\alpha|$ and $\rho'(0) = \mathcal{F}_{\mathbb{Z}_2^x} \sigma_c^x |\mathbb{Z}_2\rangle \langle\mathbb{Z}_2| \sigma_c^x + \sum_\beta \rho'_{\beta\beta}(0) |\beta\rangle \langle\beta|$ for $|\mathbb{Z}_2\rangle$ and $\sigma_c^x |\mathbb{Z}_2\rangle$ respectively, the final density matrices after evolution are given by:

$$\rho(t) = \mathcal{F}_{\mathbb{Z}_2} \cdot |\mathbb{Z}_2(t)\rangle \langle\mathbb{Z}_2(t)| + \sum_{\alpha} \rho_{\alpha\alpha}(0) |\alpha(t)\rangle \langle\alpha(t)| \tag{S12}$$

for imperfect $|\mathbb{Z}_2\rangle$ and:

$$\rho'(t) = \mathcal{F}_{\mathbb{Z}_2^x} \cdot |\mathbb{Z}_2^x(t)\rangle \langle\mathbb{Z}_2^x(t)| + \sum_{\beta} \rho_{\beta\beta}(0) |\beta(t)\rangle \langle\beta(t)| \tag{S13}$$

for imperfect $\sigma_c^x |\mathbb{Z}_2\rangle$. Here, $|\mathbb{Z}_2(t)\rangle = e^{-iHt} |\mathbb{Z}_2\rangle$ and $|\mathbb{Z}_2^x(t)\rangle = e^{-iHt} \sigma_c^x |\mathbb{Z}_2\rangle$ represent the final states after evolution for the ideal $|\mathbb{Z}_2\rangle$ and $\sigma_c^x |\mathbb{Z}_2\rangle$ initial states under the Rydberg Hamiltonian H, respectively. $\mathcal{F}_{\mathbb{Z}_2}$ and $\mathcal{F}_{\mathbb{Z}_2^x}$ denote the preparation fidelity for $|\mathbb{Z}_2\rangle$ and $\sigma_c^x |\mathbb{Z}_2\rangle$, respectively. The summation terms account for contributions from the evolution of the various initial error states $|\alpha\rangle$ and $|\beta\rangle$: $|\alpha(t)\rangle = e^{-iHt} |\alpha\rangle$ and $|\beta(t)\rangle = e^{-iHt} |\beta\rangle$, with weights $\rho_{\alpha\alpha}(0)$ and $\rho_{\beta\beta}(0)$ representing their respective probabilities in the initial density matrices.

Using these final states, we calculate the reduced density matrices for each qubit:

$$\rho_j(t) = \mathrm{Tr}_{i\neq j} \rho(t) \tag{S14}$$

$$\rho'_j(t) = \mathrm{Tr}_{i\neq j} \rho'(t) \tag{S15}$$

where $\mathrm{Tr}_{i\neq j}$ denotes the partial trace over all qubits except the j-th qubit. And the Holevo information is then calculated following the equation (S9).

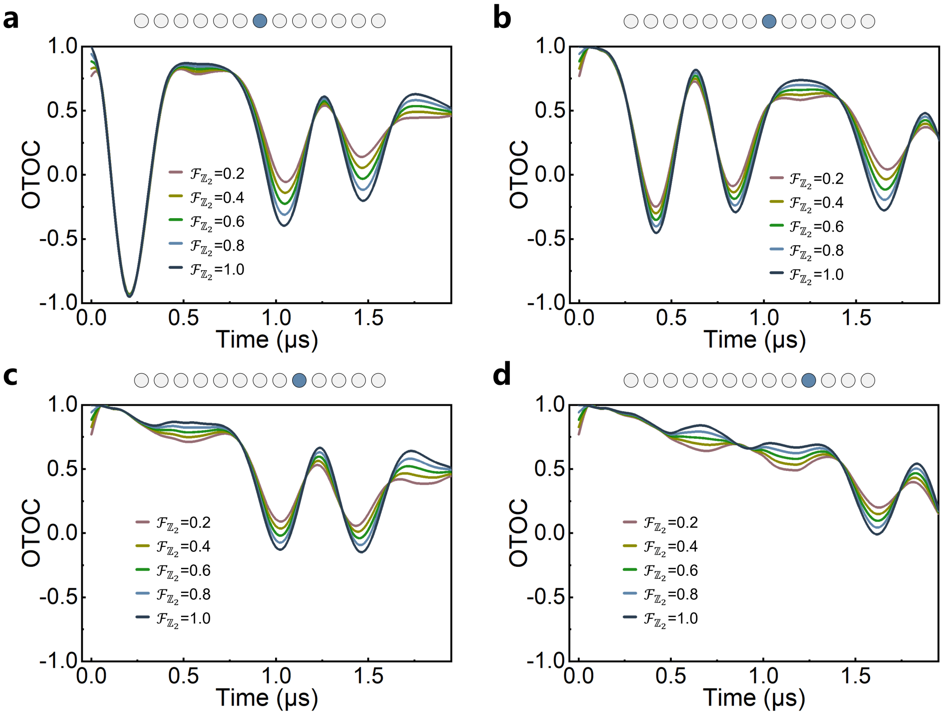

Supplementary Fig. 13. **Impact of $|\mathbb{Z}_2\rangle$ state preparation fidelity on ZZ-OTOC dynamics. a–d** show ZZ-OTOC evolution for four qubits exhibiting non-trivial dynamics. The coloured curves represents increasing fidelity values (0.2–1.0). The blue circle in the qubit chain diagram (top) indicates the qubit in each plot.

To assess the impact of imperfect initial state preparation on the Holevo information dynamics, we performed numerical simulations with different initial state preparation fidelities. Figure 15a–d demonstrates the Holevo information dynamics for initial state preparation fidelity ranging from 0.2 to 1.0. The results suggest that for low preparation fidelities, the dynamics after 1 μs (driving Rabi frequency $\sim 2\pi \times 1.2\,\mathrm{MHz}$) is degraded, particularly for qubits far away from center. These results further emphasize the necessity of high $|\mathbb{Z}_2\rangle$ state preparation fidelity for observing the *collapse-and-revival* of quantum information. Based on the measured microstates combinations of the experimental prepared initial states, equal preparation fidelities for both $|\mathbb{Z}_2\rangle$ and $\sigma_c^x |\mathbb{Z}_2\rangle$ states were assumed in the numerical simulations, with error states uniformly distributed across all $|\uparrow\rangle$-initialized qubits. To this end, Fig. 15e,f compare the Holevo information dynamics from the experimentally prepared $|\mathbb{Z}_2\rangle$ and $\sigma_c^x |\mathbb{Z}_2\rangle$ states (dashed lines) with perfectly initial states (solid lines). The results suggest that, given the experimentally achieved high initial state preparation fidelity, the distinctive features of Holevo information dynamics persist and remain readily discernible.

4.2. Detection and evolution error

Imperfections in quantum state evolution and detection introduce errors into experimental results, which degrade the performance of the Rydberg quantum simulator. This section identifies and analyzes two main categories of error: detection errors and evolution errors.

1. Detection error

The gap time (as described in section 3.1) between the end of evolution and the start of detection results in Rydberg-state atoms decaying to the ground state due to their finite lifetime, contributing to detection errors. Additionally, measurements of both Rydberg and ground states are subject to inherent detection errors (quantum state discrimination error).

To quantify these errors, we introduce two parameters: ε to represent the detection error for the Rydberg state and η to account for atomic loss in the ground state, both of which arise from the factors mentioned above. The experimentally measured ground state population, $P(\downarrow)$, can then be expressed as:

$$P(\downarrow) = \varepsilon(1-\eta)P'(\uparrow) + (1-\eta)P'(\downarrow) \tag{S16}$$

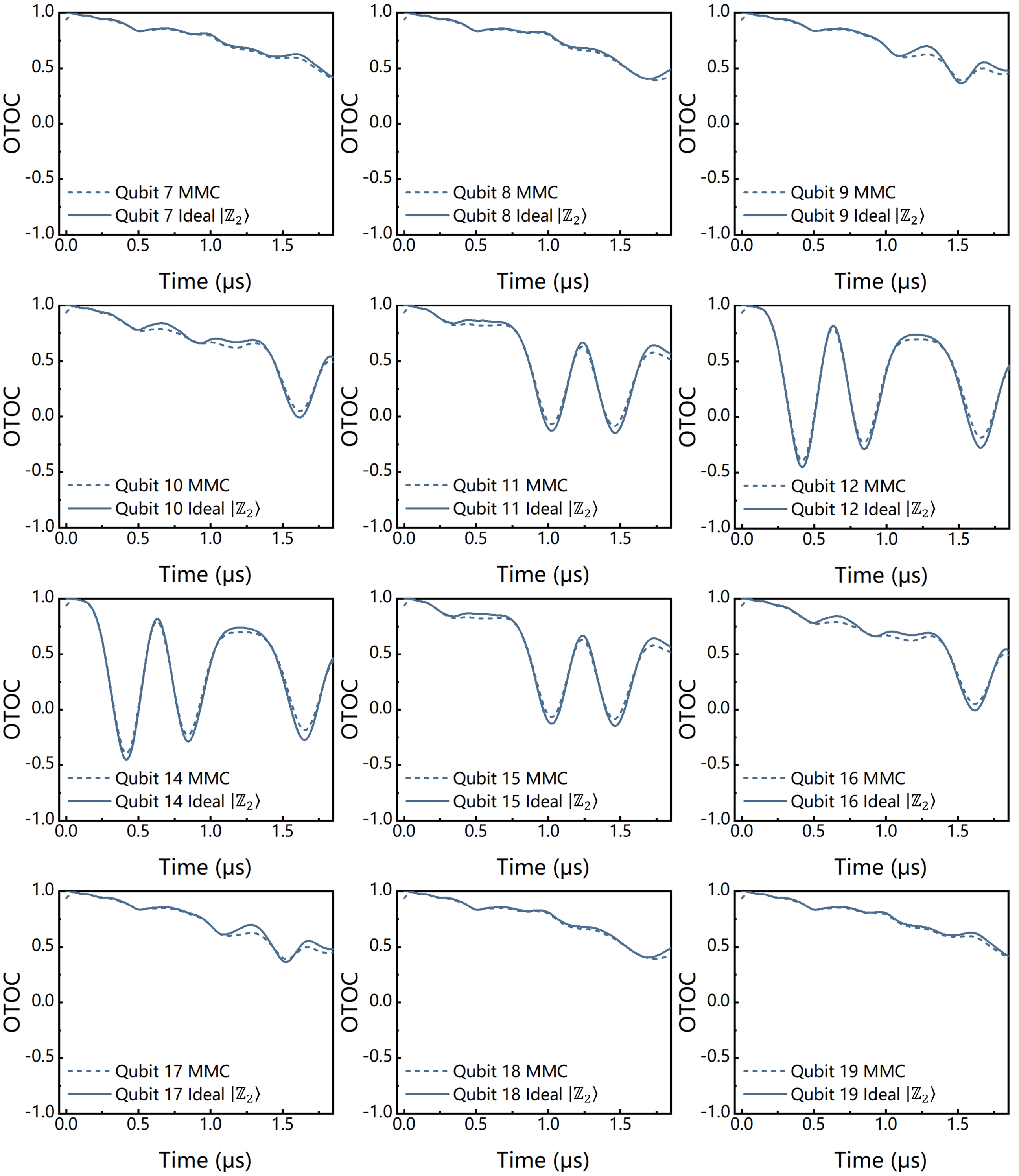

Supplementary Fig. 14. **Impact of experimental $|\mathbb{Z}_2\rangle$ state preparation errors on ZZ-OTOC dynamics.** Solid and dashed lines represent numerical simulations of OTOC evolution with the experimentally measured microstate combination (MMC) and perfect $|\mathbb{Z}_2\rangle$ state as initial states, respectively, showing a negligible difference for each qubit in the 13-atom array. The results for the central (13th) qubit are shown in Figure 3b of the main text.

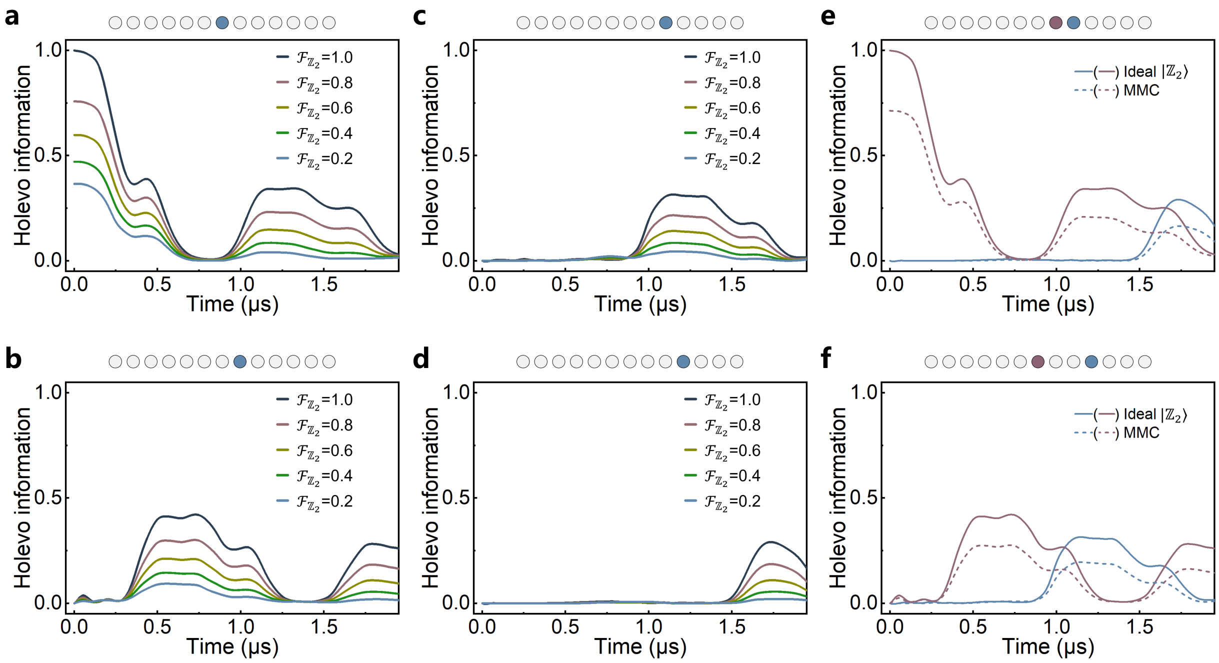

Supplementary Fig. 15. **Impact of initial state preparation fidelity on Holevo information dynamics.** **a**–**d**, Simulated Holevo information dynamics with initial state preparation fidelities varying from 0.2 to 1. **e** and **f**, Solid lines represent dynamics with perfect initial states, while dashed lines show dynamics with experimentally prepared initial state fidelities. The qubit chain above each plot indicates the positions of the qubits under consideration (coloured circles).

Here, $P'(\uparrow)$ and $P'(\downarrow)$ represent the actual Rydberg and ground state population after experimental evolution, respectively.

2. Evolution error

While we consider only two states (ground state $|\downarrow\rangle$ and Rydberg state $|\uparrow\rangle$) in numerical simulations, a third state (intermediate state $|e\rangle = |5P_{3/2}\rangle$, with a linewidth of $\Gamma_e \approx 2\pi \times 6.06\,\mathrm{MHz}$) is involved in the evolution driven by the Raman lasers, and introduces incoherent errors in the dynamics of OTOC and Holevo information. The 480-nm (780-nm) Raman laser couples the $|\uparrow\rangle$ ($|\downarrow\rangle$) state to $|e\rangle$, leading to unwanted scattering and depolarization between $|\downarrow\rangle$ and $|\uparrow\rangle$. Furthermore, the radiative lifetime of the Rydberg state also contributes to the depolarization during the evolution process. These effects can be summed up and characterized by one parameter, the depolarization time T_1, accounting for the amplitude damping of both OTOC and Holevo information oscillations.

Another major error source is coherent evolution error, also referred to as evolution noises. During the evolution, two dominant noise sources emerge: fluctuations in the relative phase between $|\downarrow\rangle$ and $|\uparrow\rangle$, and variations in the Rabi frequency. These sources contribute to non-unitarity in the forward-and-backward Hamiltonian evolution and decoherence in Rabi oscillations. The time-dependent noisy Rydberg Hamiltonian is modeled as:

$$H(t) = \sum_i \left[\frac{\Omega(t) e^{-\mathrm{i}\phi(t)}}{2} \sigma_i^x - \Delta(t) n_i \right] + \sum_{i<j} V_{ij}(t) n_i n_j \tag{S17}$$

Here, $\phi(t)$ and $\Omega(t)$ represent the time-dependent phase and Rabi frequency, respectively. $\Delta(t)$ accounts for time-dependent laser frequency detuning. These time-dependent fluctuations contribute to the single-atom decoherence time T_2^*, arising from various sources including laser noises and Doppler effects. Additionally, $V_{ij}(t)$ denotes the time-dependent Rydberg-Rydberg interaction strength between atoms i and j in the many-body system, whose uncertainty is introduced by atomic motion and the disorder in the initial atomic distance.

In our experiment, as described in section 1.1, we employ the Pound-Drever-Hall (PDH) technique to frequency-stabilize the Rydberg excitation laser to a ULE cavity. This method effectively suppresses laser frequency noise below the cavity linewidth. We treat the high-frequency noises above the linewidth in two components: one attributed to servo bumps[149,150], and the remaining noise, which can be modeled as spectrally uniform (white) phase noise[151]. As a result, $\Delta(t)$ in equation (S17) can be approximated by a Gaussian distribution with a root-mean-square (RMS)

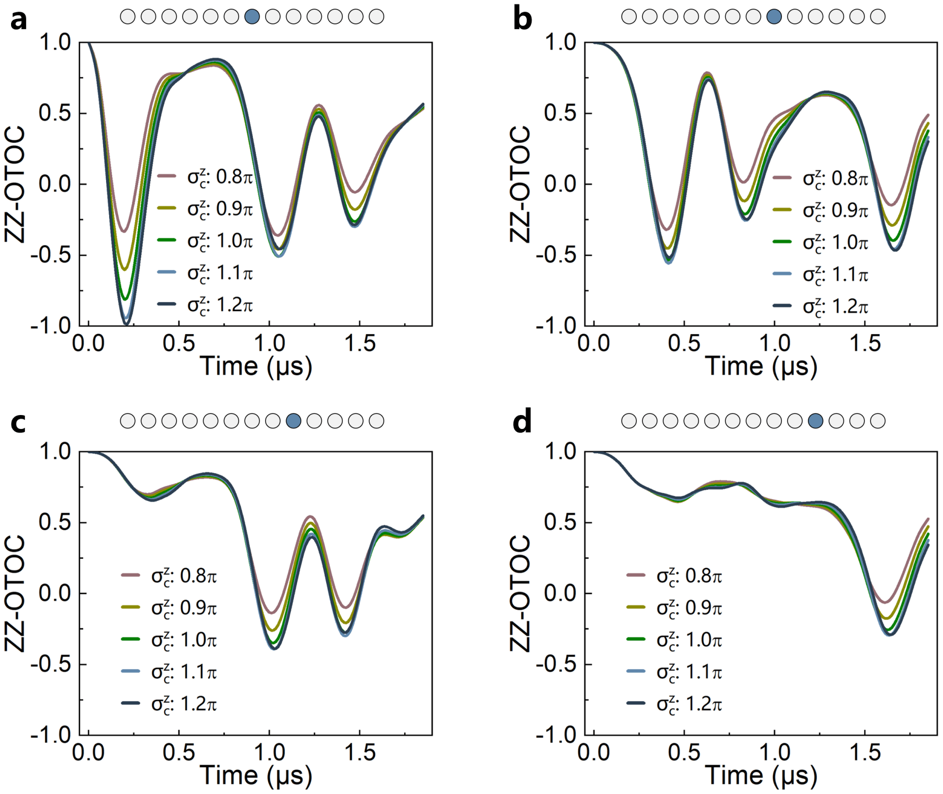

Supplementary Fig. 16. **Effect of local perturbation fidelity on ZZ-OTOC measurements.** Simulated ZZ-OTOC dynamics for four representative qubits in a 13-qubit chain, demonstrating the impact of σ_i^z fidelity on OTOC oscillations. The σ_c^z fidelity is represented by the accumulated phase, ranging from 0.8π to 1.2π. **a-d**, ZZ-OTOC evolution for different qubits. The qubit configuration is shown above each plot, with the orange circle indicating the qubit under consideration.

amplitude of $\delta\Delta$. Moreover, variations in laser power and spatial inhomogeneities induce Gaussian-type perturbations in the Rabi frequency, characterized by an RMS amplitude of $\delta\Omega$. Additionally, the parameter $\delta\phi$ is introduced to represent the uncertainty in $\phi(t)$, to account for the fluctuations in the relative phase between the Rydberg state and the ground state during the evolution process, typically arising from the servo bump of the excitation lasers.

The fidelity of the local perturbation σ_c^z also significantly impacts the experimentally measured ZZ-OTOC values. Numerical simulations show that the fidelity of σ_c^z operations directly affects the contrast of OTOC oscillations within the light cone. This relationship is illustrated in Fig. 16, presenting simulation results for a 13-qubit array. Since the σ_c^z gate is accomplished with a relative π phase shift between the Rydberg state and ground state induced by far-detuned 795-nm addressing laser beams, the infidelity mainly stems from uncertainty in the accumulated phase.

This comprehensive error model captures the primary sources of noise in our system. By identifying and formalizing these errors, we establish a framework for accurately interpreting experimental results. This approach provides a solid foundation for error benchmarking and mitigation protocols, which are crucial for enhancing the accuracy of OTOC and Holevo information measurements in probing quantum information collapse and revival.

4.3. Error characterization

To quantify and mitigate errors in our quantum simulator, we conducted a series of calibration experiments to characterize the error sources identified in our model.

1. Detection error

We systematically characterize detection errors in our system. For atoms in the ground state, the raw detection error η is approximately 1%. For Rydberg states, the detection error is more complex, consisting of a raw error $\varepsilon' \approx 1\%$ along with an additional time-dependent component arising from the finite Rydberg state lifetime.

To quantify this time dependence, we measure the lifetime T_R of the Rydberg state used in our experiment. Atoms

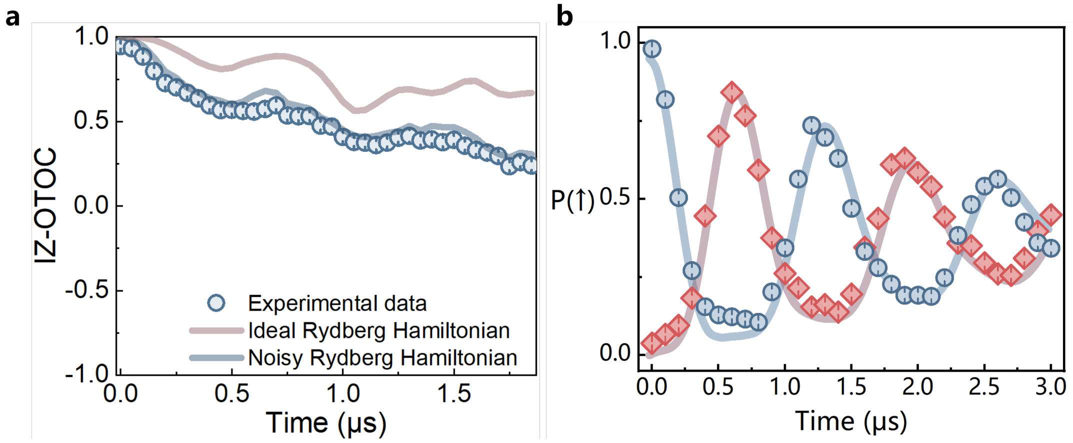

Supplementary Fig. 17. **Investigating the decay mechanisms in OTOC and Holevo information dynamics.** **a**, Comparison of experimental data (blue points, corrected for detection errors and the incoherent errors introduced by the depolarization effect) with numerical simulations using different Hamiltonians (ideal Rydberg Hamiltonian not considering any experimental noise – for red curve and noisy Rydberg Hamiltonian– including experimental noise– for blue curve) for IZ-OTOC with an initial $|\mathbb{Z}_2\rangle$ state. The imperfect inversion of the Hamiltonian during the evolution causes the IZ-OTOC to be less than 1 in the ideal case (red curve). Experimental noise further induces the decay of the IZ-OTOC. **b**, Rydberg Hamiltonian evolution dynamics of initial $|\mathbb{Z}_2\rangle$ state. Blue circles and red diamonds represent corrected experimental results for $|\uparrow\rangle$- and $|\downarrow\rangle$-initialized state, respectively, while solid lines show corresponding numerical simulations. The excellent agreement confirms that our understanding of the decay mechanisms in OTOC and Holevo information dynamics is accurate.

are first prepared in the Rydberg state using a global Raman π-pulse, after which we vary the time interval between the π-pulse and the population measurement. The population measurement is performed by turning on the optical tweezers to recapture ground state atoms while repelling the Rydberg atoms. An exponential fit to the data yields a $1/e$ time constant of $T_R = 140(15)\,\mu\text{s}$. Given the interval time t_i, which depends on the evolution time t in our sequence, the Rydberg state detection error accumulates over time. This error is expressed as $\varepsilon'(t) = 1 - e^{-t_i/T_R}$, representing the probability of Rydberg atoms decaying to the ground state during the interval.

2. Evolution error

Evolution errors in our system arise from three main sources: the depolarization caused by spontaneous emission, the finite temperature of the atoms, and laser noise.

Depolarization effects. We accounted for depolarization time T_1 due to spontaneous emission via the intermediate state and Rydberg state radiative decay. The error probability of a Rydberg state decaying to the ground state during evolution time t is approximated as $\eta = \gamma t$ with $\gamma t \ll 1$. Here, $\gamma = 1/T_1$ is treated as a free parameter due to the complexity of many-body evolution. This complexity arises from two factors: (1) during evolution, different initial states (e.g., $|\mathbb{Z}_2\rangle$ and $|\mathbf{0}\rangle$) lead to variations in the average Rydberg population, resulting in different effective decay rates; and (2) the exponentially growing Hilbert space and complex interactions in many-body state evolution cause the effective decay rate to differ from the more easily measured decay rate in single-atom evolution.

Finite-temperature effects. The thermal motion of atoms leads to two effects: fluctuations in atomic positions and Doppler shifts. Position fluctuations affect the Rydberg-Rydberg interactions, which scale as $1/R^6$, where R is the inter-atomic distance. We estimated the standard deviation of position fluctuations to be about $0.3\,\mu\text{m}$, directly impacting the strength of Rydberg-Rydberg interactions. Doppler shifts, on the other hand, introduce frequency detuning in the Rydberg excitation. We characterized these thermal effects by measuring the average temperature of the atoms at the beginning of evolution using the release and recapture method, finding it to be approximately $10\,\mu\text{K}$. From this, we calculated the standard deviation of the atomic velocity distribution as $\sigma_v = \sqrt{k_B T/M}$, where k_B is the Boltzmann constant, T is the temperature, and M is the atomic mass. For our counter-propagating two-photon excitation scheme with a 480-nm σ^+-polarized and a 780-nm σ^+-polarized light, this corresponds to a Doppler broadening with a standard deviation of $\delta\Delta_1 = k\sigma_v \approx 2\pi \times 25\,\text{kHz}$, where $k \approx 1.25\,\mu\text{m}^{-1}$ is the effective two-photon wave vector.

Laser noise. We characterized both intensity and phase noise of our laser sources. The Rabi frequency fluctuation $\delta\Omega/\Omega$ is related to the laser intensity fluctuation $\delta I/I$ by $\delta\Omega/\Omega \approx \delta I/(2I)$. High-bandwidth measurements of 780-nm and 480-nm Raman laser power variations using fast photodiodes revealed an RMS amplitude noise of $\delta\Omega/\Omega \approx 0.01$. For laser frequency noise, we analysed the in-loop PDH error signal at Fourier frequencies above the cavity linewidth

($\gamma_{\mathrm{cav}} \approx 2\pi \times 110\,\mathrm{kHz}$ at 960 nm and $2\pi \times 60\,\mathrm{kHz}$ at 780 nm). We estimated the laser frequency noise by integrating the noise spectral density $S_\nu(f)$:

$$\delta\Delta_2 = \sqrt{\int_{\gamma_{\mathrm{cav}}}^{f_h} S_\nu(f)df}, \tag{S18}$$

where $f_h \approx 1/\delta t$. This yields an RMS frequency noise of $\delta\Delta_2 \approx 2\pi \times 5\,\mathrm{kHz}$ for the combined 780-nm and 480-nm laser contributions. Since this frequency noise contributes to the uncertainty in the relative phase between the qubit and the driving field, $\delta\phi$, in the same way as the Doppler effect, we consider only $\delta\phi$ instead of the combination of $\delta\Delta_1$ and $\delta\Delta_2$ in the following analysis.

To further calibrate $\delta\phi$ and γ, we measured the IZ-OTOC (main text), which follows the same sequence as our ZZ-OTOC experiments but without the local perturbation. Numerical simulations treating $\delta\phi$ as a free parameter yield excellent agreement with corrected experimental data for $\delta\phi = 0.08\pi$ and $\gamma = 0.035\,\mathrm{\mu s}^{-1}$ (Fig. 17a). The difference between the numerical results of the noisy Rydberg Hamiltonian (blue curve) and the ideal case (red curve) arise from the various experimental noises discussed above. Meanwhile, in the ideal case, the IZ-OTOC being less than 1 is attributed to the imperfect inversion of the Hamiltonian during the evolution, as mentioned in section 2.1. For Holevo information evolution, we measured Rydberg Hamiltonian evolution dynamics of the initial $|\mathbb{Z}_2\rangle$ state, finding good agreement when using the same parameters (Fig. 17b).

The experimental results shown in Fig. 17a and Fig. 18 have been corrected for detection errors and partially corrected for evolution errors. Specifically, while detection errors were fully accounted for, only the evolution errors related to Rydberg state decay to the ground state (γt) were addressed. First, detection error correction was applied. Then, we subtracted the accumulating population of Rydberg states decaying to the ground state (γt) from the experimentally measured ground state population to compensate for the incoherent errors introduced by the intermediate state during the experiment.

For ZZ-OTOC measurements, an extra error source is the σ^z local perturbation infidelity. In order to characterize the uncertainty in local perturbation, we conducted a comparative analysis of the results obtained from Ramsey experiments with and without 795-nm addressing employed for local σ_i^z. The findings indicate that uncertainty is approximately 0.09π. This uncertainty contributes to the overall evolution noise and affects the fidelity of our local perturbation.

Using the noise parameters mentioned above, we simulated the dynamics of the ZZ-OTOC with initial $|\mathbb{Z}_2\rangle$ state under the noisy Rydberg Hamiltonian [equation (S17)] based on the Monte Carlo method. We compared the simulation results with the experimental results for the central 9 qubits (the qubit index definition is shown in Fig. 18 inset). For the OTOC dynamics of the initial state $|\mathbf{0}\rangle$, we employed the same error model. Figure 19b presents the comparison between the simulation results and the experimental data for the central 7 qubits. The excellent agreement between these simulations and our experimental data (Fig. 18 for $|\mathbb{Z}_2\rangle$ state and Fig. 19b for $|\mathbf{0}\rangle$ state) provides solid validation for our error model, which accounts for both detection and evolution errors, and enhances our understanding of the complex many-body dynamics.

4.4. Error mitigation for ZZ-OTOC

We employ an error mitigation scheme inspired by Swingle and Halpern[65] and Mi *et al.*[66] to address imperfections in OTOC measurements. Theoretical analysis indicates that under experimental conditions with imperfections, the errors in the measured ZZ-OTOC $F^m(W,V)$ can be effectively mitigated using the measured IZ-OTOC $F^m(I,V)$[65]:

$$F^c \approx \frac{F^m(W,V)}{F^m(I,V)}, \tag{S19}$$

where F^c represents the corrected ZZ-OTOC measurement results.

This scheme mitigates the imperfections in forward-and-backward evolution caused by coherent noise ($\delta\phi$, $\delta\Omega$, $\delta\Delta$) and partially mitigates those from next-nearest-neighbour interactions $V_{i,i+2}$. However, it cannot effectively mitigate errors outside the forward-and-backward evolution, such as imperfections in the local σ_i^z perturbation and the detection errors. Consequently, mitigated results are expected to fall between expectations of the noise-free Rydberg Hamiltonian and the ideal PXP model.

The denominator, IZ-OTOC $F^m(I,V)$, is crucial in the mitigation protocol. As demonstrated by Mi *et al.*[66], small variations in the IZ-OTOC used as the denominator can dramatically affect corrected results, particularly for near-zero IZ-OTOC values. Recognizing this sensitivity, we conducted a comprehensive analysis of factors potentially affecting OTOC measurements and quantified them (detailed in section 4.3). Notably, numerical simulations show

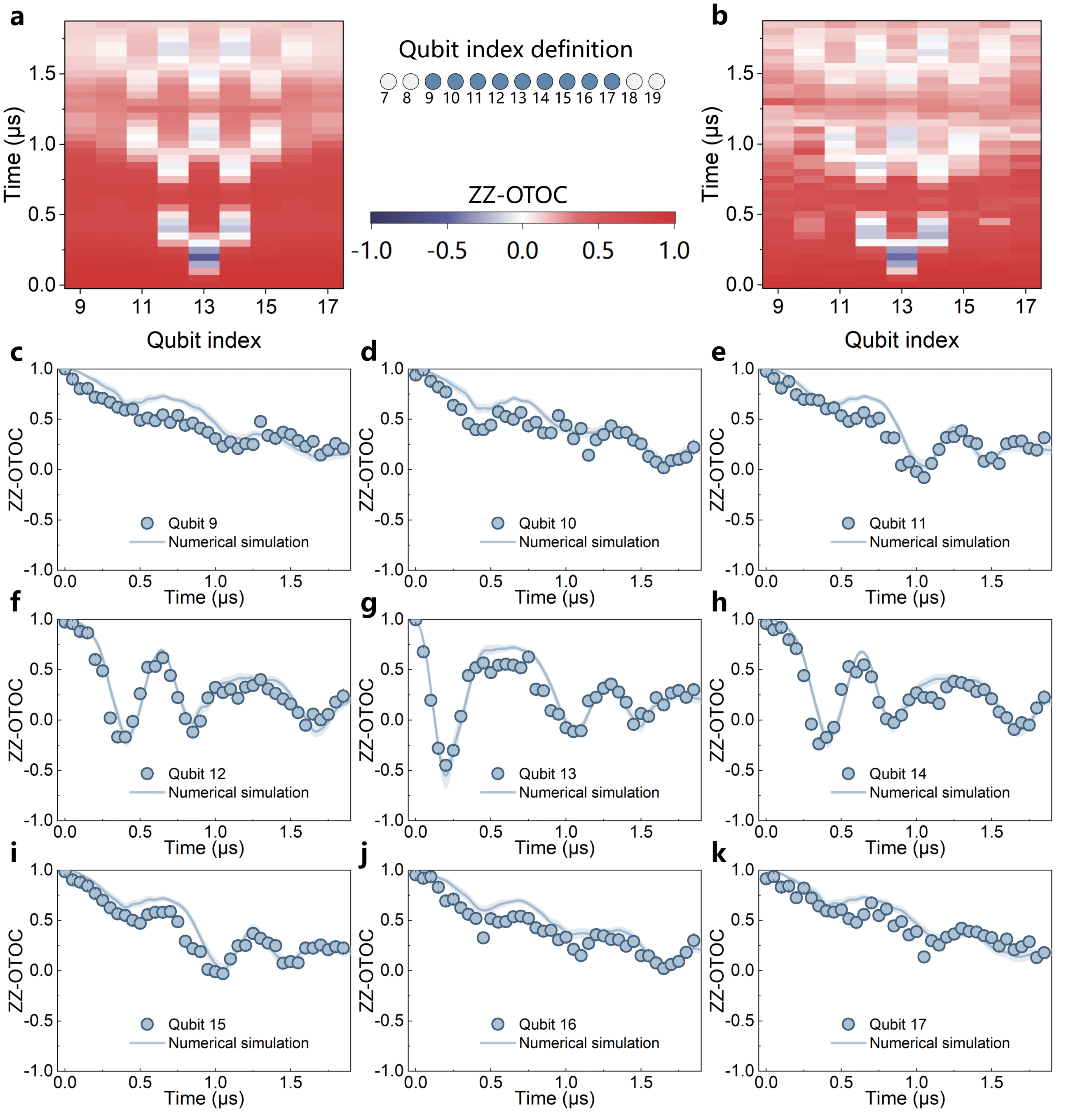

Supplementary Fig. 18. **Dynamics of ZZ-OTOC for $|\mathbb{Z}_2\rangle$ considering experimental imperfections.** **a**, Simulated spatio-temporal evolution of ZZ-OTOC for $|\mathbb{Z}_2\rangle$ state under the time-dependent noisy Hamiltonian (S17). **b**, Experimental ZZ-OTOC data for $|\mathbb{Z}_2\rangle$ state, corrected for detection errors and incoherent errors arise from the depolarization effect. Inset, The qubit index definition of the exhibited qubits (highlighted) in 13-qubit chain (top). **c**–**k**, Detailed dynamics plots of the corrected experimental data (blue points) and the simulation results (solid curve). The shaded areas around the curves represent the error bar from the numerical simulation. The corresponding qubit index of plots is respectively marked; good agreement between experimental data and numerical results is found for all qubits.

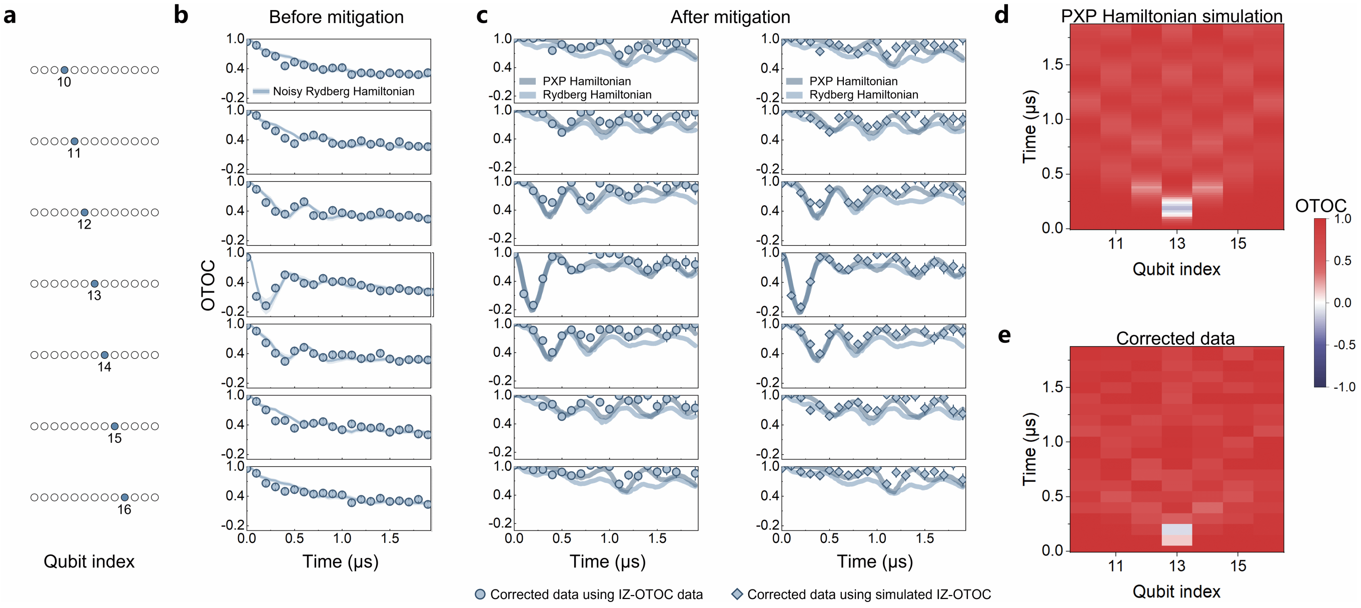

Supplementary Fig. 19. **Error mitigation for OTOC dynamics of the initial $|\mathbf{0}\rangle$ state.** **a**, Qubit index definition for each row in panels b and c (blue filled circle). The central 7 qubits from the spin chain are used for analysis. **b**, OTOC dynamics before error mitigation. Blue curves with shaded error bars represent simulations using the modeled noisy Rydberg Hamiltonian. Circles are the experimental data, demonstrating excellent agreement with the simulations. **c**, OTOC dynamics after error mitigation. Left: Experimental data corrected using measured IZ-OTOC (circles). Right: Experimental data corrected using simulated IZ-OTOC (diamonds). In both cases, light blue curves represent simulations with the ideal Rydberg Hamiltonian, while dark blue curves show the ideal PXP Hamiltonian dynamics. **d**–**e**, Spatio-temporal OTOC dynamics. **d**, Simulated OTOC dynamics for the central 7 qubits using the PXP Hamiltonian, incorporating imperfections from local perturbations. **e**, Experimental data corresponding to the left panel data in **c**. The colour bar corresponds to the values of the OTOC. The excellent agreement between corrected data and simulations demonstrates the effectiveness of the error mitigation scheme.

significant edge effects in small-sized chains ($L < 10$). Comparing the edge atom's ZZ-OTOC versus IZ-OTOC as the denominator for mitigation in a noisy environment shows that using the edge atom's ZZ-OTOC leads to over-correction and temporal misalignment at critical positions (Fig. 6d). In contrast, using IZ-OTOC produces results that align with theoretical predictions. To minimize edge effects, we rely on IZ-OTOC measurements instead of the edge atom's ZZ-OTOC for error mitigation.

The numerical simulations are conducted to evaluate the effectiveness of this error mitigation scheme in the context of our experimental imperfections. Figure. 20a shows the numerically simulated ZZ-OTOC dynamics using the noisy Rydberg Hamiltonian in equation (S17), which incorporates major experimental imperfections (as Fig. 18). The mitigated case (Fig. 20b) closely resembles the PXP Hamiltonian with local perturbation imperfections shown in Fig. 20c, indicating that the mitigation scheme successfully addresses forward-and-backward evolution noise. However, when compared to the ideal PXP Hamiltonian without imperfections (Fig. 20d), the mitigated case shows slightly less pronounced features. This subtle difference can be attributed to the local perturbation imperfections which can not be mitigated.

As the first step in error mitigation, we correct the detection errors, i.e., the imperfections in measurement operator σ_j^z. Next, we correct the evolution errors. Given the excellent agreement between experimental IZ-OTOC data and numerical simulations of IZ-OTOC (as shown in Fig. 17a), we can effectively use either the experimental IZ-OTOC data or the simulated IZ-OTOC results to mitigate the experimental ZZ-OTOC data shown in Fig. 18c–k. The mitigated results of the initial $|\mathbb{Z}_2\rangle$ state are presented in Fig. 6 (using the measured IZ-OTOC data) and Fig. 21 (using the simulated IZ-OTOC results). Figure 19c shows the mitigated results of the initial $|\mathbf{0}\rangle$ state using the measured IZ-OTOC data (left) and the simulated IZ-OTOC results (right). All the results demonstrate significant improvement in the agreement between mitigated experimental data and theoretical expectations for the ZZ-OTOC (with imperfections in local perturbation σ_i^z, light blue curve under ideal PXP Hamiltonian while the dark blue under ideal Rydberg Hamiltonian). This excellent agreement underscores the effectiveness of our error mitigation protocol in addressing the complex noise landscape of the Rydberg quantum simulator.

This approach effectively overcomes experimental imperfections, particularly those associated with forward-and-backward evolution in OTOC measurements, enabling accurate probe of quantum information dynamics in Rydberg

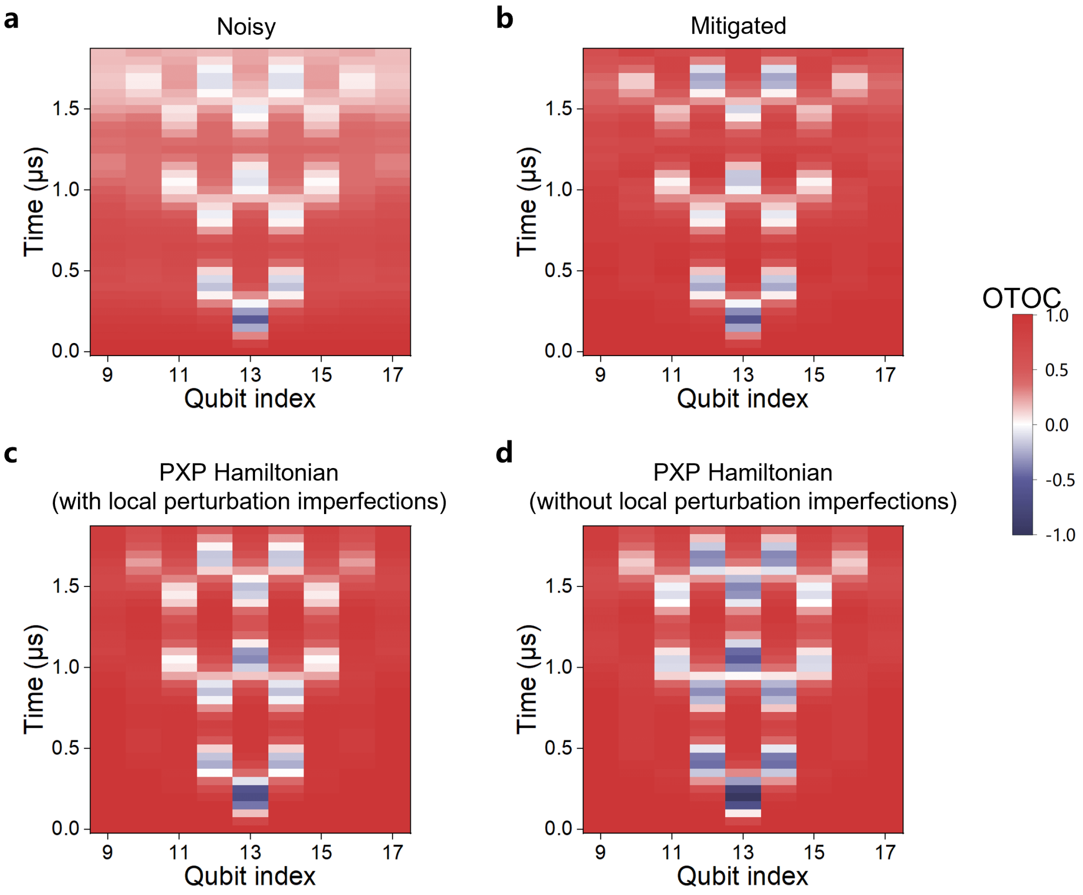

Supplementary Fig. 20. **Numerical simulation of error mitigation scheme for ZZ-OTOC. a**, Simulated ZZ-OTOC dynamics for $|\mathbb{Z}_2\rangle$ considering experimental imperfections same as Fig. 18a. **b**, Mitigated ZZ-OTOCs using simulated IZ-OTOC results, exhibiting enhanced *collapse-and-revival* contrast compared to **a**. **c**–**d**, Simulated ZZ-OTOC dynamics using the PXP Hamiltonian while accounting for local perturbation imperfections (**c**) or not (**d**). The closer resemblance of the mitigated data **b** to **c** demonstrates that our error mitigation scheme cannot mitigate local perturbation imperfections. The colour scale represents ZZ-OTOC values from -1.0 (blue) to 1.0 (red).

atom quantum simulators.

4.5. Error mitigation for Holevo information

The detection error for Holevo information can be mitigated similarly to how OTOCs are handled. The measured diagonal elements, which are linear transformations of $P(\uparrow)$, are directly corrected for detection errors, and the off-diagonal elements can be extracted from sinusoidal fittings of detection-error-corrected Ramsey oscillations. However, evolution errors are more complex and cannot be easily mitigated because they are deeply intertwined with the quantum information dynamics. Due to the difficulty in determining whether quantum information initially encoded in a qubit is lost due to evolution errors or transferred to other qubits, it is very challenging to apply traditional error mitigation techniques that focus on compensating for single-qubit decoherence. Therefore, evolution errors are not mitigated for Holevo information. Instead, they are included in the numerical simulations of Holevo information dynamics, together with state preparation errors, showing good agreement with experimental data (Fig. 22).

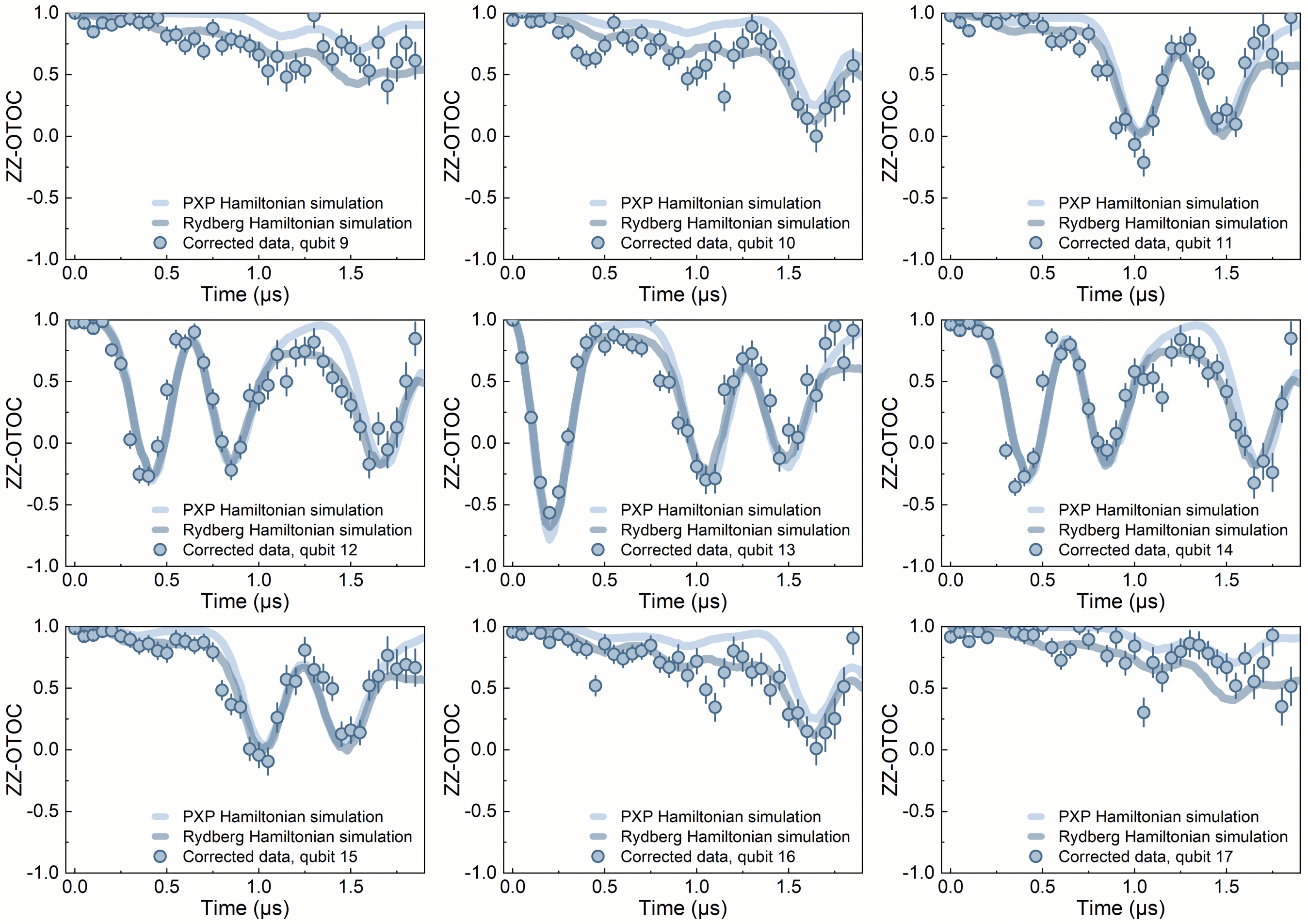

Supplementary Fig. 21. **Mitigating ZZ-OTOC for $|\mathbb{Z}_2\rangle$ state using the simulated IZ-OTOC results.** Blue points marked with qubit indices represent the mitigated experimental data using simulated IZ-OTOC results. Dark blue curves show simulated ZZ-OTOC dynamics for the initial $|\mathbb{Z}_2\rangle$ state with the ideal Rydberg Hamiltonian (equation (S1), no gap time during the OTOC evolution). Light blue curves display simulated ZZ-OTOC dynamics for the initial $|\mathbb{Z}_2\rangle$ state with the ideal PXP Hamiltonian. The mitigated experimental data show excellent agreement with simulations.

5. KINETICALLY CONSTRAINED DYNAMICS AND QUANTUM INFORMATION COLLAPSE-AND-REVIVAL

5.1. Investigation of kinetically constrained dynamics

To characterize the constrained spin dynamics in our Rydberg atom chain, we developed a method to identify and analyse the wavefront of excitations. This approach is particularly effective for the $|\mathbb{Z}_2\rangle$ state, where individual spins exhibit periodic but non-sinusoidal oscillations, often with phase differences between neighbouring atoms. Our wavefront detection method identifies the moments when adjacent atoms have equal Rydberg excitation probabilities, $P_i(\uparrow) = P_{i+1}(\uparrow)$, based on numerical simulations (Fig. 2e,g in the main text). By connecting these time points for each nearest-neighbour atom pair, we construct wavefronts that capture the propagation of excitations throughout the system. This technique allows us to study distinct behaviours for different initial configurations.

Our simulations indicate that the $|\mathbb{Z}_2\rangle$ state exhibits uniform wavefront propagation throughout the bulk of the spin chain, consistent with the synchronized evolution observed in the experiment. This synchronization arises from the interplay between the PXP constraints and the initial $|\mathbb{Z}_2\rangle$ configuration. In this regime, each spin experiences a similar effective environment due to the alternating pattern of its neighbours, leading to coherent and synchronized rotations across the bulk of the system.

However, near the edges of the chain, deviations from this synchronized behaviour begin to appear. These boundary effects manifest as distortions in the wavefront shape, reflecting the altered local environment of the outermost atoms.

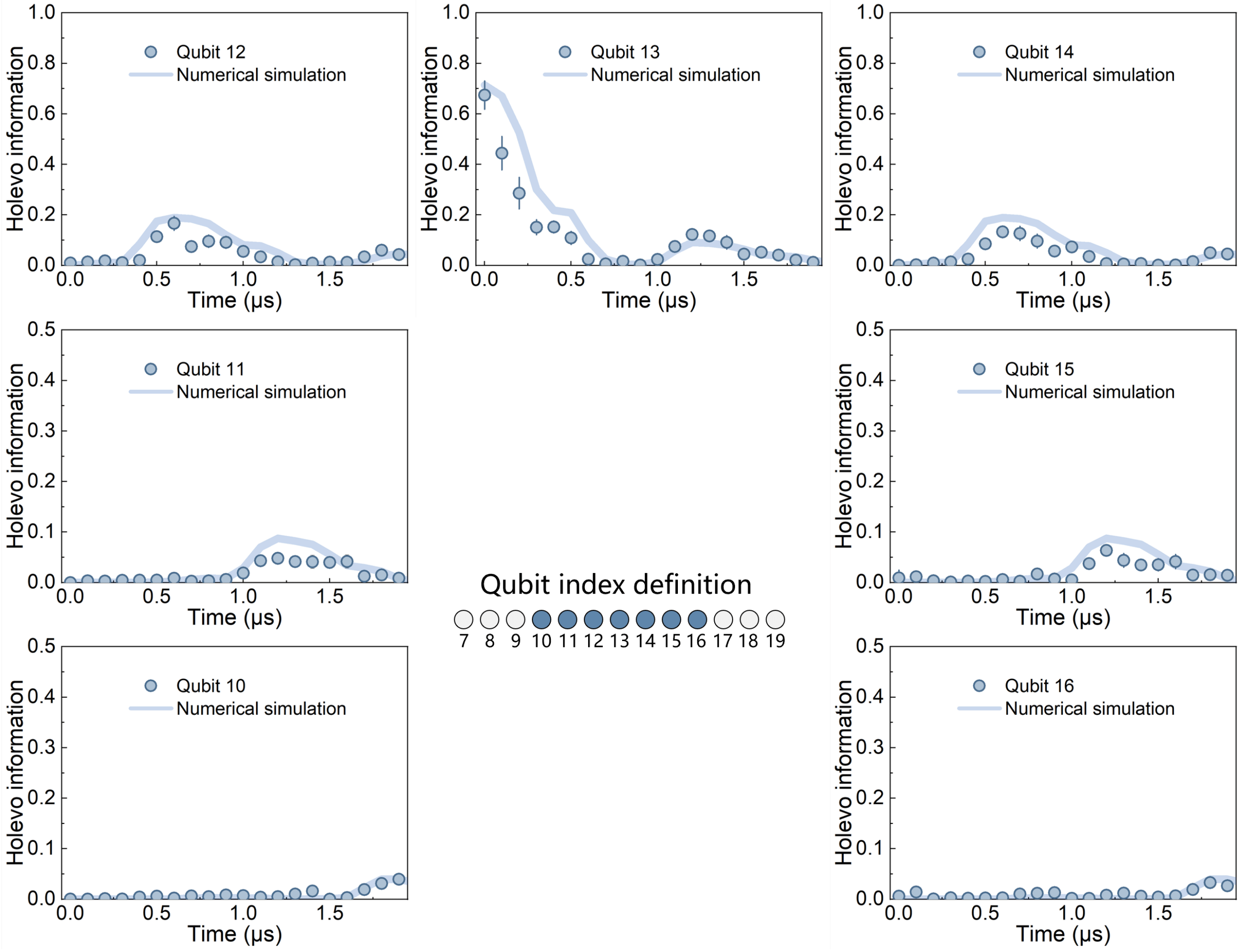

Supplementary Fig. 22. **Experimental data and numerical simulations for Holevo information dynamics.** Holevo information dynamics of the detection-error-corrected experimental data (blue points), compared to the numerical simulation results (solid curves). The inset highlights the index definitions of 7 selected qubits within the 13-qubit chain. Monte Carlo methods are employed in the simulation to account for imperfections during initial state preparation and evolution.

Near the boundaries, the lack of symmetry and the different neighbouring structure cause spins to evolve out of sync with those in the central region. This gradual desynchronization, moving from the outer edges toward the center, aligns with the boundary effects described in the main text. Figure 2e in the main text provides a spatial map of the wavefront propagation, clearly illustrating the transition from uniform propagation in the bulk to distorted behaviour at the edges.

For the $\sigma_c^x |\mathbb{Z}_2\rangle$ state with the central spin flipped, we observe rich dynamical behaviour characterized by a clear linear light cone structure, as discussed in the main text. The flipped central spin introduces retardation in adjacent spins' rotation, which propagates outwards as the system evolves. Inside the light cone, there is an interplay between periodic spin rotations and retardations due to the kinetically constrained dynamics. This results in an arc-shaped, curved wavefront that moves outward from the initial perturbation at the central spin. Figure 2g in the main text illustrates this light cone structure and the corresponding wavefront propagation. The clear visualization of the light cone and wavefront behaviour offers a valuable tool for understanding the kinetically constrained quantum many-body systems.

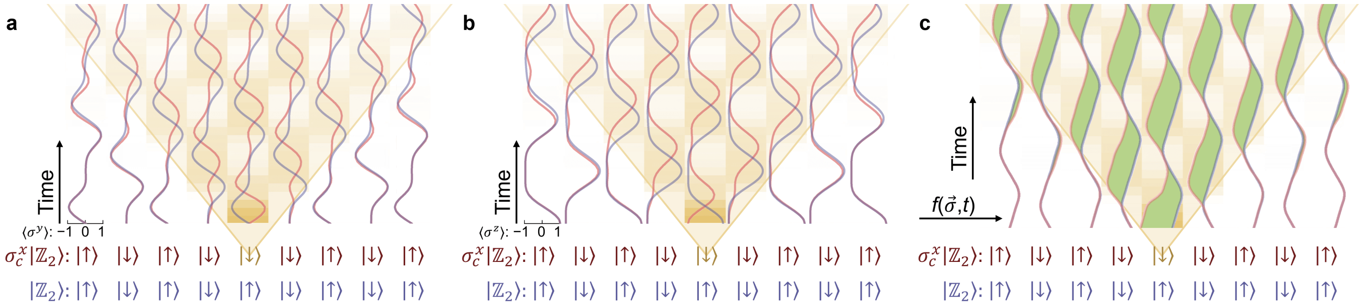

Supplementary Fig. 23. **Illustration of constrained qubit dynamics in PXP model.** **a**, Dynamics of $\langle\sigma^y\rangle$ and **b**, $\langle\sigma^z\rangle$ for each qubit. **c**, The dynamic indicator $f(\vec{\sigma},t)=\int_0^t \mathrm{d}\{\mathrm{Arg}[\vec{\sigma}(\tau)]\}\mathrm{d}\tau-\lambda\Omega t$, which represents the total rotation angle of the Bloch vector in the YZ-plane. The blue curves correspond to the initial state $|\mathbb{Z}_2\rangle$, while the red curves represent the initial state $\sigma_c^x|\mathbb{Z}_2\rangle$, where the central spin is flipped. The green-filled intervals in **c** indicate regions of retardation and distinguishability between the two initial states. Yellow lines connect the divergence points, forming light cones (yellow shaded areas) where the dynamics of $\sigma_c^x|\mathbb{Z}_2\rangle$ are periodically delayed. The checkerboard background in all panels shows the heatmap of the Holevo information dynamics (dark yellow: $\mathbb{X}_j(t)=1$; transparent: $\mathbb{X}_j(t)=0$).

5.2. Illustration of quantum information collapse-and-revival in PXP model

As demonstrated in the main text, the *collapse-and-revival* behaviour of quantum information observed here originates from constrained qubit dynamics. Specifically, the Rydberg blockade effect causes a delayed rotation of spins near the central flipped spin, which creates regions of delayed spin rotation that propagate outwards. Figure 23 highlights the relationship between constrained qubit dynamics and Holevo information, employing three dynamic indicators to characterize two key concepts: retardation and distinguishability.

The background heatmaps in Fig. 23 display the numerical simulation results of the Holevo information under the PXP Hamiltonian, which mirrors Fig. 4c in the main text. Figure 23a,b depict the oscillations in the expectation values of $\langle\sigma^y\rangle$ and $\langle\sigma^z\rangle$, respectively. These oscillations indicate that when the Holevo information approaches zero inside the light cone, the Bloch vectors $\vec{\sigma}$ for each qubit become indistinguishable, sharing the same $\langle\sigma^y\rangle$ and $\langle\sigma^z\rangle$ values.

To provide a clearer understanding of the peaks in Holevo information, we introduce a new dynamic indicator:

$$f(\vec{\sigma},t)=\int_0^t \mathrm{d}\{\mathrm{Arg}[\vec{\sigma}(\tau)]\}\mathrm{d}\tau-\lambda\Omega t, \tag{S20}$$

where the first term, $\int_0^t \mathrm{d}\{\mathrm{Arg}[\vec{\sigma}(\tau)]\}\mathrm{d}\tau$, represents the total rotation angle of the Bloch vector $\vec{\sigma}$ in the YZ-plane. This provides a more fundamental perspective on the spin dynamics than the individual expectation values of $\langle\sigma^y\rangle$ and $\langle\sigma^z\rangle$.

To restrict the range of the rotation angle which accumulates almost monotonically over time, we subtract the term $\lambda\Omega t$ in the expression for $f(\vec{\sigma},t)$, where Ω is the Rabi frequency in the PXP Hamiltonian. The factor $\lambda=1.32$ is extracted from the slope of a linear fit with all the simulation data for the total rotation angle of each qubit with two initial states $|\mathbb{Z}_2\rangle$ and $\sigma_c^x|\mathbb{Z}_2\rangle$. As shown in Fig. 23c, the retardation can be extracted directly from the difference between the blue and red curves (highlighted by the green-filled intervals), which is the source of distinguishability in the $\langle\sigma^y\rangle$ and $\langle\sigma^z\rangle$ measurements. Within the light cone, the time delay remains nearly constant, but the rotation angle retardation exhibits a periodic *collapse-and-revival* pattern.

It is clear that both the retardation and Holevo information follow similar *collapse-and-revival* dynamics. This analysis of constrained qubit dynamics provides insights into the mechanisms behind quantum information propagation and the *collapse-and-revival* behaviour in dynamically constrained systems. The dynamic indicator, $f(\vec{\sigma},t)$, offers a way to visualize and quantify the relationship between spin dynamics and information flow. These results contribute to a better understanding of quantum information behaviour in constrained systems.

5.3. Distinguishing scar state oscillations and quantum information collapse-and-revival

In our experiment, we observe a clear light-cone structure in the dynamics of both OTOCs and Holevo information, with periodic *collapse-and-revival* behaviour within the light cone. Here, we show that these *collapse-and-revival* dynamics in quantum information are relevant, but not equivalent to the previously discovered oscillations of quantum

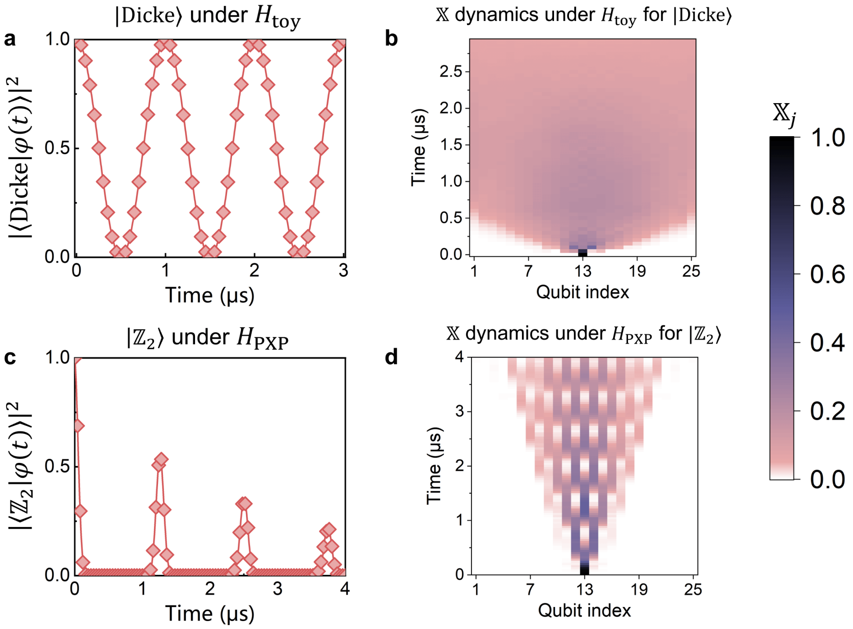

Supplementary Fig. 24. **Comparison of quantum information dynamics in scar states between the toy model and the PXP model.** **a**, Perfect scarred state wavefunction oscillations under ideal toy model Hamiltonian H_{toy} evolution. The simulated wavefunction overlap between the evolved state $|\varphi(t)\rangle$ and the initial state $|\text{Dicke}\rangle$ is shown, as a function of evolution time t. The state $|\varphi(t)\rangle$ is obtained after evolving the initial $|\text{Dicke}\rangle$ state under the ideal toy model Hamiltonian H_{toy} for time t. **b**, Numerically simulated spatio-temporal dynamics of the Holevo information for $|\text{Dicke}\rangle$ and $\sigma_c^x\,|\text{Dicke}\rangle$ initial states, under ideal toy model Hamiltonian H_{toy} evolution. **c**, Damped scarred state wavefunction oscillations under ideal PXP model Hamiltonian H_{PXP} evolution. The simulated wavefunction overlap between the evolved state $|\varphi(t)\rangle$ and the initial state $|\mathbb{Z}_2\rangle$ is shown, as a function of evolution time t. The state $|\varphi(t)\rangle$ is obtained after evolving the initial $|\mathbb{Z}_2\rangle$ state under the ideal PXP model Hamiltonian H_{PXP} for time t. **d**, Numerically simulated spatio-temporal dynamics of the Holevo information for $|\mathbb{Z}_2\rangle$ and $\sigma_c^x\,|\mathbb{Z}_2\rangle$ initial states, under ideal PXP model Hamiltonian H_{PXP} evolution.

scar states wavefunction under the PXP Hamiltonian evolution. The relevance between these two phenomena primarily stems from the fact that the physical mechanism driving both the oscillation of quantum many-body scar wavefunctions and the quantum information *collapse-and-revival* observed in our experiment originates from the kinetic constraints in the PXP model. Their distinction arises because the dynamics of OTOCs and Holevo information are not confined to the scarred subspace but also incorporate contributions from thermal eigenstates. For instance, in the measurement of Holevo information dynamics, the σ_c^x operation, which flips the central spin in the chain, introduces a mixture of scarred subspace and thermal eigenstate bath contributions. Such a mixture precludes attributing the periodic behaviour of Holevo information solely to the eigenstate decomposition of the initial state. Therefore, the observed information backflow within the light cone cannot be simply attributed to the oscillation of quantum scar states wavefunction.

Building on the above discussion, it becomes clear that the quantum information *collapse-and-revival* observed in our experiment is not equivalent to the oscillations of quantum scar states wavefunctions under the Hamiltonian evolution. This raises an intriguing question: more generally, does the presence of quantum many-body scar state oscillations always imply the existence of quantum information *collapse-and-revival*? Furthermore, could there be physical systems where quantum many-body scar state oscillations occur without any accompanying quantum information *collapse-and-revival*? To explore these questions, we turn to a toy model proposed by Choi *et al.*[49], described by the Hamiltonian:

$$H_{\text{toy}} = \frac{\Omega}{2}\sum_i \sigma_i^x + \sum_i V_{i-1,i+2}P_{i,i+1} \tag{S21}$$

Here $P_{i,j} = (1 - \vec{\sigma}_i \cdot \vec{\sigma}_j)/4$ is the projection operator onto the singlet state of spins at sites i and j, and $V_{i,j} = \sum_{\mu\nu} J_{ij}^{\mu\nu}\sigma_i^\mu\sigma_j^\nu$ represents an arbitrary long-range interaction between spins at sites i and j. Dicke states,

expressed as $|s = L/2, S^x = m_x\rangle$, are the scarred eigenstates of H_{toy}, as the interaction terms annihilate these states ($P_{i,j}\,|s = L/2, S^x = m_x\rangle = 0$).

We perform numerical simulations for this toy model with $L = 25$ spins with periodic boundary conditions, using the parameters $\Omega = 2\pi \times 1$ MHz and $V_{i,j} = J(\sigma_i^x \sigma_j^y + \sigma_i^y \sigma_j^x)$ with $J = 2\pi \times 2$ MHz. When initialized in the scarred Dicke state $|\text{Dicke}\rangle = |s = L/2, S^z = -L/2\rangle = |\downarrow\downarrow \cdots \downarrow\rangle$, the system exhibits perfect scarred state wavefunction oscillations (Fig. 24a). Next, we explore quantum information scrambling and transport within the toy model using the scar state. Similar to our study of Holevo information for the $|\mathbb{Z}_2\rangle$ scar state in the PXP model, we applied a central spin flip to the scarred Dicke state in the toy model, denoted as $\sigma_c^x\,|\text{Dicke}\rangle$. We then simulated the evolution of both $\sigma_c^x\,|\text{Dicke}\rangle$ and $|\text{Dicke}\rangle$ under the toy model Hamiltonian. From these simulations, we obtained the spatio-temporal evolution of Holevo information, allowing us to investigate how quantum information propagates and scrambles in this system. Figure 24b shows the simulated Holevo information dynamics, with clear evidence of a rapid, global scrambling. This global scrambling behaviour differs significantly from the kinetically constrained PXP model, where the spatial-temporal *collapse-and-revival* of quantum information is very pronounced (Fig. 24d). In the scar state $|\text{Dicke}\rangle$ of the H_{toy}, initially encoded quantum information is quickly lost to the environment without revival.

These results show that, under the toy model Hamiltonian H_{toy}, while the scarred state $|\text{Dicke}\rangle$ exhibits perfect wavefunction oscillations, no *collapse-and-revival* of quantum information occurs. This suggests that the oscillatory behaviour of quantum many-body scar states does not necessarily coincide with periodic quantum information backflow. The quantum information spatial-temporal *collapse-and-revival* dynamics observed in this work is likely a very unique feature resulting from the kinetic constraints imposed by the Rydberg blockade effect.

SUPPLEMENTARY REFERENCES

[1] Piotrowicz, M. J. *et al.* Two-dimensional lattice of blue-detuned atom traps using a projected Gaussian beam array. *Phys. Rev. A* **88**, 013420 (2013).

[2] Endres, M. *et al.* Atom-by-atom assembly of defect-free one-dimensional cold atom arrays. *Science* **354**, 1024–1027 (2016).

[3] Barredo, D., de Léséleuc, S., Lienhard, V., Lahaye, T. & Browaeys, A. An atom-by-atom assembler of defect-free arbitrary two-dimensional atomic arrays. *Science* **354**, 1021–1023 (2016).

[4] Lee, W., Kim, H. & Ahn, J. Defect-free atomic array formation using the Hungarian matching algorithm. *Phys. Rev. A* **95**, 053424 (2017).

[5] Barredo, D., Lienhard, V., de Léséleuc, S., Lahaye, T. & Browaeys, A. Synthetic three-dimensional atomic structures assembled atom by atom. *Nature* **561**, 79–82 (2018).

[6] Kumar, A., Wu, T.-Y., Giraldo, F. & Weiss, D. S. Sorting ultracold atoms in a three-dimensional optical lattice in a realization of Maxwell's demon. *Nature* **561**, 83–87 (2018).

[7] Sheng, C. *et al.* Defect-free arbitrary-geometry assembly of mixed-species atom arrays. *Phys. Rev. Lett.* **128**, 083202 (2022).

[8] Barnes, K. *et al.* Assembly and coherent control of a register of nuclear spin qubits. *Nat. Commun.* **13**, 2779 (2022).

[9] Okuno, D. *et al.* High-resolution spectroscopy and single-photon Rydberg excitation of reconfigurable ytterbium atom tweezer arrays utilizing a metastable state. *J. Phys. Soc. Jpn.* **91**, 084301 (2022).

[10] Liu, Y. *et al.* Realization of strong coupling between deterministic single-atom arrays and a high-finesse miniature optical cavity. *Phys. Rev. Lett.* **130**, 173601 (2023).

[11] Pause, L. *et al.* Supercharged two-dimensional tweezer array with more than 1000 atomic qubits. *Optica* **11**, 222–226 (2024).

[12] Isenhower, L. *et al.* Demonstration of a neutral atom controlled-NOT quantum gate. *Phys. Rev. Lett.* **104**, 010503 (2010).

[13] Jau, Y.-Y., Hankin, A. M., Keating, T., Deutsch, I. H. & Biedermann, G. W. Entangling atomic spins with a Rydberg-dressed spin-flip blockade. *Nat. Phys.* **12**, 71–74 (2016).

[14] Sheng, C. *et al.* High-fidelity single-qubit gates on neutral atoms in a two-dimensional magic-intensity optical dipole trap array. *Phys. Rev. Lett.* **121**, 240501 (2018).

[15] Omran, A. *et al.* Generation and manipulation of Schrödinger cat states in Rydberg atom arrays. *Science* **365**, 570–574 (2019).

[16] Guo, R. *et al.* Balanced coherence times of atomic qubits of different species in a dual 3× 3 magic-intensity optical dipole trap array. *Phys. Rev. Lett.* **124**, 153201 (2020).

[17] Bluvstein, D. *et al.* A quantum processor based on coherent transport of entangled atom arrays. *Nature* **604**, 451–456 (2022).

[18] Graham, T. M. *et al.* Multi-qubit entanglement and algorithms on a neutral-atom quantum computer. *Nature* **604**, 457–462 (2022).

[19] Fu, Z. *et al.* High-fidelity entanglement of neutral atoms via a Rydberg-mediated single-modulated-pulse controlled-phase gate. *Phys. Rev. A* **105**, 042430 (2022).

[20] Schine, N., Young, A. W., Eckner, W. J., Martin, M. J. & Kaufman, A. M. Long-lived Bell states in an array of optical clock qubits. *Nat. Phys.* **18**, 1067–1073 (2022).

[21] Singh, K. *et al.* Mid-circuit correction of correlated phase errors using an array of spectator qubits. *Science* **380**, 1265–1269 (2023).

[22] Evered, S. J. *et al.* High-fidelity parallel entangling gates on a neutral-atom quantum computer. *Nature* **622**, 268–272 (2023).
[23] Scholl, P. *et al.* Erasure conversion in a high-fidelity Rydberg quantum simulator. *Nature* **622**, 273–278 (2023).
[24] Ma, S. *et al.* High-fidelity gates and mid-circuit erasure conversion in an atomic qubit. *Nature* **622**, 279–284 (2023).
[25] Zhao, L., Lee, M. D. K., Aliyu, M. M. & Loh, H. Floquet-tailored Rydberg interactions. *Nat. Commun.* **14**, 7128 (2023).
[26] Bluvstein, D. *et al.* Logical quantum processor based on reconfigurable atom arrays. *Nature* **626**, 58–65 (2023).
[27] Gregory, P. D. *et al.* Second-scale rotational coherence and dipolar interactions in a gas of ultracold polar molecules. *Nat. Phys.* **20**, 415–421 (2024).
[28] Shaw, A. L. *et al.* Benchmarking highly entangled states on a 60-atom analogue quantum simulator. *Nature* **628**, 71–77 (2024).
[29] Ebadi, S. *et al.* Quantum optimization of maximum independent set using Rydberg atom arrays. *Science* **376**, 1209–1215 (2022).
[30] Kim, M., Kim, K., Hwang, J., Moon, E.-G. & Ahn, J. Rydberg quantum wires for maximum independent set problems. *Nat. Phys.* **18**, 755–759 (2022).
[31] Bernien, H. *et al.* Probing many-body dynamics on a 51-atom quantum simulator. *Nature* **551**, 579–584 (2017).
[32] Keesling, A. *et al.* Quantum Kibble–Zurek mechanism and critical dynamics on a programmable Rydberg simulator. *Nature* **568**, 207–211 (2019).
[33] Ebadi, S. *et al.* Quantum phases of matter on a 256-atom programmable quantum simulator. *Nature* **595**, 227–232 (2021).
[34] Scholl, P. *et al.* Quantum simulation of 2d antiferromagnets with hundreds of Rydberg atoms. *Nature* **595**, 233–238 (2021).
[35] Semeghini, G. *et al.* Probing topological spin liquids on a programmable quantum simulator. *Science* **374**, 1242–1247 (2021).
[36] Fang, F. *et al.* Probing critical phenomena in open quantum systems using atom arrays. *Science* **390**, 601–605 (2025).
[37] de Léséleuc, S. *et al.* Observation of a symmetry-protected topological phase of interacting bosons with Rydberg atoms. *Science* **365**, 775–780 (2019).
[38] Scholl, P. *et al.* Microwave engineering of programmable XXZ Hamiltonians in arrays of Rydberg atoms. *PRX Quantum* **3**, 020303 (2022).
[39] Chen, C. *et al.* Continuous symmetry breaking in a two-dimensional Rydberg array. *Nature* **616**, 691–695 (2023).
[40] Kim, K., Yang, F., Mølmer, K. & Ahn, J. Realization of an extremely anisotropic Heisenberg magnet in Rydberg atom arrays. *Phys. Rev. X* **14**, 011025 (2024).
[41] Bornet, G. *et al.* Scalable spin squeezing in a dipolar Rydberg atom array. *Nature* **621**, 728–733 (2023).
[42] Eckner, W. J. *et al.* Realizing spin squeezing with Rydberg interactions in an optical clock. *Nature* **621**, 734–739 (2023).
[43] Deutsch, J. M. Quantum statistical mechanics in a closed system. *Phys. Rev. A* **43**, 2046–2049 (1991).
[44] Srednicki, M. Chaos and quantum thermalization. *Phys. Rev. E* **50**, 888–901 (1994).
[45] Rigol, M., Dunjko, V. & Olshanii, M. Thermalization and its mechanism for generic isolated quantum systems. *Nature* **452**, 854–858 (2008).
[46] Deutsch, J. M. Eigenstate thermalization hypothesis. *Rep. Prog. Phys.* **81**, 082001 (2018).
[47] Lewis-Swan, R. J., Safavi-Naini, A., Kaufman, A. M. & Rey, A. M. Dynamics of quantum information. *Nat. Rev. Phys.* **1**, 627–634 (2019).
[48] Turner, C. J., Michailidis, A. A., Abanin, D. A., Serbyn, M. & Papić, Z. Weak ergodicity breaking from quantum many-body scars. *Nat. Phys.* **14**, 745–749 (2018).
[49] Choi, S. *et al.* Emergent SU(2) dynamics and perfect quantum many-body scars. *Phys. Rev. Lett.* **122**, 220603 (2019).
[50] Ho, W. W., Choi, S., Pichler, H. & Lukin, M. D. Periodic orbits, entanglement, and quantum many-body scars in constrained models: Matrix product state approach. *Phys. Rev. Lett.* **122**, 040603 (2019).
[51] Bluvstein, D. *et al.* Controlling quantum many-body dynamics in driven Rydberg atom arrays. *Science* **371**, 1355–1359 (2021).
[52] Turner, C. J., Desaules, J.-Y., Bull, K. & Papić, Z. Correspondence principle for many-body scars in ultracold Rydberg atoms. *Phys. Rev. X* **11**, 021021 (2021).
[53] Serbyn, M., Abanin, D. A. & Papić, Z. Quantum many-body scars and weak breaking of ergodicity. *Nat. Phys.* **17**, 675–685 (2021).
[54] Zhang, P. *et al.* Many-body Hilbert space scarring on a superconducting processor. *Nat. Phys.* **19**, 120–125 (2022).
[55] Su, G.-X. *et al.* Observation of many-body scarring in a Bose-Hubbard quantum simulator. *Phys. Rev. Res.* **5**, 023010 (2023).
[56] Maskara, N. *et al.* Discrete time-crystalline order enabled by quantum many-body scars: Entanglement steering via periodic driving. *Phys. Rev. Lett.* **127**, 090602 (2021).
[57] Dong, H. *et al.* Disorder-tunable entanglement at infinite temperature. *Sci. Adv.* **9**, eadj3822 (2023).
[58] Liu, L.-Y.-N. *et al.* Novel ground states and emergent quantum many-body scars in a two-species rydberg atom array. *Preprint at https://arxiv.org/abs/2408.15965* (2024).
[59] Desaules, J.-Y. *et al.* Ergodicity breaking under confinement in cold-atom quantum simulators. *Quantum* **8**, 1274 (2024).
[60] Kerschbaumer, A., Ljubotina, M., Serbyn, M. & Desaules, J.-Y. Quantum many-body scars beyond the pxp model in rydberg simulators. *Phys. Rev. Lett.* **134**, 160401 (2025).
[61] Yuan, D., Zhang, S.-Y., Wang, Y., Duan, L.-M. & Deng, D.-L. Quantum information scrambling in quantum many-body scarred systems. *Phys. Rev. Res.* **4**, 023095 (2022).
[62] Lesanovsky, I. & Katsura, H. Interacting Fibonacci anyons in a Rydberg gas. *Phys. Rev. A* **86**, 041601 (2012).
[63] Cheng, Y. & Zhai, H. Emergent U(1) lattice gauge theory in Rydberg atom arrays. *Nat. Rev. Phys.* **6**, 566–576 (2024).

[64] Hauschild, J. & Pollmann, F. Efficient numerical simulations with Tensor Networks: Tensor Network Python (TeNPy). *SciPost Phys. Lect. Notes* 5 (2018).
[65] Swingle, B. & Halpern, N. Y. Resilience of scrambling measurements. *Phys. Rev. A* **97**, 062113 (2018).
[66] Mi, X. *et al.* Information scrambling in quantum circuits. *Science* **374**, 1479–1483 (2021).
[67] Lieb, E. H. & Robinson, D. W. The finite group velocity of quantum spin systems. *Commun. Math. Phys.* **28**, 251–257 (1972).
[68] Hastings, M. B. & Koma, T. Spectral gap and exponential decay of correlations. *Commun. Math. Phys.* **265**, 781–804 (2006).
[69] Sekino, Y. & Susskind, L. Fast scramblers. *J. High Energy Phys.* **2008**, 065–065 (2008).
[70] Hauke, P. & Tagliacozzo, L. Spread of correlations in long-range interacting quantum systems. *Phys. Rev. Lett.* **111**, 207202 (2013).
[71] Eisert, J., van den Worm, M., Manmana, S. R. & Kastner, M. Breakdown of quasilocality in long-range quantum lattice models. *Phys. Rev. Lett.* **111**, 260401 (2013).
[72] Foss-Feig, M., Gong, Z.-X., Clark, C. W. & Gorshkov, A. V. Nearly linear light cones in long-range interacting quantum systems. *Phys. Rev. Lett.* **114**, 157201 (2015).
[73] Maldacena, J., Shenker, S. H. & Stanford, D. A bound on chaos. *J. High Energy Phys.* **2016**, 1–17 (2016).
[74] von Keyserlingk, C. W., Rakovszky, T., Pollmann, F. & Sondhi, S. L. Operator hydrodynamics, OTOCs, and entanglement growth in systems without conservation laws. *Phys. Rev. X* **8**, 021013 (2018).
[75] Nahum, A., Vijay, S. & Haah, J. Operator spreading in random unitary circuits. *Phys. Rev. X* **8**, 021014 (2018).
[76] Nachtergaele, B., Sims, R. & Young, A. Quasi-locality bounds for quantum lattice systems. I. Lieb-Robinson bounds, quasi-local maps, and spectral flow automorphisms. *J. Math. Phys.* **60** (2019).
[77] Else, D. V., Machado, F., Nayak, C. & Yao, N. Y. Improved Lieb-Robinson bound for many-body Hamiltonians with power-law interactions. *Phys. Rev. A* **101**, 022333 (2020).
[78] Gong, Z. & Hamazaki, R. Bounds in nonequilibrium quantum dynamics. *Int. J. Mod. Phys. B* **36**, 2230007 (2022).
[79] Chen, C.-F. A., Lucas, A. & Yin, C. Speed limits and locality in many-body quantum dynamics. *Rep. Prog. Phys.* **86**, 116001 (2023).
[80] Cheneau, M. *et al.* Light-cone-like spreading of correlations in a quantum many-body system. *Nature* **481**, 484–487 (2012).
[81] Langen, T., Geiger, R., Kuhnert, M., Rauer, B. & Schmiedmayer, J. Local emergence of thermal correlations in an isolated quantum many-body system. *Nat. Phys.* **9**, 640–643 (2013).
[82] Richerme, P. *et al.* Non-local propagation of correlations in quantum systems with long-range interactions. *Nature* **511**, 198–201 (2014).
[83] Jurcevic, P. *et al.* Quasiparticle engineering and entanglement propagation in a quantum many-body system. *Nature* **511**, 202–205 (2014).
[84] Hosur, P., Qi, X.-L., Roberts, D. A. & Yoshida, B. Chaos in quantum channels. *J. High Energy Phys.* **2016**, 1–49 (2016).
[85] Kukuljan, I., Grozdanov, S. & Prosen, T. Weak quantum chaos. *Phys. Rev. B* **96**, 060301 (2017).
[86] Kaufman, A. M. *et al.* Quantum thermalization through entanglement in an isolated many-body system. *Science* **353**, 794–800 (2016).
[87] Brydges, T. *et al.* Probing Rényi entanglement entropy via randomized measurements. *Science* **364**, 260–263 (2019).
[88] Zhu, Q. *et al.* Observation of thermalization and information scrambling in a superconducting quantum processor. *Phys. Rev. Lett.* **128**, 160502 (2022).
[89] Nandkishore, R. & Huse, D. A. Many-body localization and thermalization in quantum statistical mechanics. *Annu. Rev. Condens. Matter Phys.* **6**, 15–38 (2015).
[90] Schreiber, M. *et al.* Observation of many-body localization of interacting fermions in a quasirandom optical lattice. *Science* **349**, 842–845 (2015).
[91] Choi, J.-y. *et al.* Exploring the many-body localization transition in two dimensions. *Science* **352**, 1547–1552 (2016).
[92] Abanin, D. A., Altman, E., Bloch, I. & Serbyn, M. Colloquium: Many-body localization, thermalization, and entanglement. *Rev. Mod. Phys.* **91**, 021001 (2019).
[93] Lukin, A. *et al.* Probing entanglement in a many-body–localized system. *Science* **364**, 256–260 (2019).
[94] Rispoli, M. *et al.* Quantum critical behaviour at the many-body localization transition. *Nature* **573**, 385–389 (2019).
[95] Huang, Y., Zhang, Y. & Chen, X. Out-of-time-ordered correlators in many-body localized systems. *Ann. Phys. (Berlin)* **529**, 1600318 (2017).
[96] Chen, X., Zhou, T., Huse, D. A. & Fradkin, E. Out-of-time-order correlations in many-body localized and thermal phases. *Ann. Phys. (Berlin)* **529**, 1600332 (2017).
[97] Deng, D.-L., Li, X., Pixley, J. H., Wu, Y.-L. & Das Sarma, S. Logarithmic entanglement lightcone in many-body localized systems. *Phys. Rev. B* **95**, 024202 (2017).
[98] He, R.-Q. & Lu, Z.-Y. Characterizing many-body localization by out-of-time-ordered correlation. *Phys. Rev. B* **95**, 054201 (2017).
[99] Swingle, B. & Chowdhury, D. Slow scrambling in disordered quantum systems. *Phys. Rev. B* **95**, 060201 (2017).
[100] Fan, R., Zhang, P., Shen, H. & Zhai, H. Out-of-time-order correlation for many-body localization. *Sci. Bull.* **62**, 707–711 (2017).
[101] Bañuls, M. C., Yao, N., Choi, S., Lukin, M. D. & Cirac, J. I. Dynamics of quantum information in many-body localized systems. *Phys. Rev. B* **96**, 174201 (2017).
[102] Hayden, P. & Preskill, J. Black holes as mirrors: quantum information in random subsystems. *J. High Energy Phys.* **2007**, 120–120 (2007).

[103] Almheiri, A., Marolf, D., Polchinski, J. & Sully, J. Black holes: complementarity or firewalls? *J. High Energy Phys.* **2013**, 1–20 (2013).
[104] Shenker, S. H. & Stanford, D. Black holes and the butterfly effect. *J. High Energy Phys.* **2014**, 1–25 (2014).
[105] Shenker, S. H. & Stanford, D. Stringy effects in scrambling. *J. High Energy Phys.* **2015**, 1–34 (2015).
[106] Qi, X.-L. Does gravity come from quantum information? *Nat. Phys.* **14**, 984–987 (2018).
[107] Landsman, K. A. *et al.* Verified quantum information scrambling. *Nature* **567**, 61–65 (2019).
[108] Li, Z. *et al.* Improving metrology with quantum scrambling. *Science* **380**, 1381–1384 (2023).
[109] Larkin, A. I. & Ovchinnikov, Y. N. Quasiclassical method in the theory of superconductivity. *Sov. Phys. JETP* **28**, 1200–1205 (1969).
[110] Swingle, B. Unscrambling the physics of out-of-time-order correlators. *Nat. Phys.* **14**, 988–990 (2018).
[111] Xu, S. & Swingle, B. Accessing scrambling using matrix product operators. *Nat. Phys.* **16**, 199–204 (2020).
[112] Xu, S. & Swingle, B. Scrambling dynamics and out-of-time-ordered correlators in quantum many-body systems. *PRX quantum* **5**, 010201 (2024).
[113] Chen, Y. Universal logarithmic scrambling in many body localization. *Preprint at https://arxiv.org/abs/1608.02765* (2016).
[114] Hashimoto, K., Murata, K. & Yoshii, R. Out-of-time-order correlators in quantum mechanics. *J. High Energy Phys.* **2017**, 1–31 (2017).
[115] Blok, M. S. *et al.* Quantum information scrambling on a superconducting qutrit processor. *Phys. Rev. X* **11**, 021010 (2021).
[116] Braumüller, J. *et al.* Probing quantum information propagation with out-of-time-ordered correlators. *Nat. Phys.* **18**, 172–178 (2021).
[117] Zhao, S. *et al.* Probing operator spreading via Floquet engineering in a superconducting circuit. *Phys. Rev. Lett.* **129**, 160602 (2022).
[118] Wang, J.-H. *et al.* Information scrambling dynamics in a fully controllable quantum simulator. *Phys. Rev. Res.* **4**, 043141 (2022).
[119] Gärttner, M. *et al.* Measuring out-of-time-order correlations and multiple quantum spectra in a trapped-ion quantum magnet. *Nat. Phys.* **13**, 781–786 (2017).
[120] Joshi, M. K. *et al.* Quantum information scrambling in a trapped-ion quantum simulator with tunable range interactions. *Phys. Rev. Lett.* **124**, 240505 (2020).
[121] Green, A. M. *et al.* Experimental measurement of out-of-time-ordered correlators at finite temperature. *Phys. Rev. Lett.* **128**, 140601 (2022).
[122] Li, J. *et al.* Measuring out-of-time-order correlators on a nuclear magnetic resonance quantum simulator. *Phys. Rev. X* **7**, 031011 (2017).
[123] Wei, K. X., Ramanathan, C. & Cappellaro, P. Exploring localization in nuclear spin chains. *Phys. Rev. Lett.* **120**, 070501 (2018).
[124] Wei, K. X. *et al.* Emergent prethermalization signatures in out-of-time ordered correlations. *Phys. Rev. Lett.* **123**, 090605 (2019).
[125] Sánchez, C. M. *et al.* Perturbation independent decay of the Loschmidt echo in a many-body system. *Phys. Rev. lett.* **124**, 030601 (2020).
[126] Niknam, M., Santos, L. F. & Cory, D. G. Sensitivity of quantum information to environment perturbations measured with a nonlocal out-of-time-order correlation function. *Phys. Rev. Res.* **2**, 013200 (2020).
[127] Nie, X. *et al.* Experimental observation of equilibrium and dynamical quantum phase transitions via out-of-time-ordered correlators. *Phys. Rev. Lett.* **124**, 250601 (2020).
[128] Chen, B. *et al.* Detecting the out-of-time-order correlations of dynamical quantum phase transitions in a solid-state quantum simulator. *Appl. Phys. Lett.* **116** (2020).
[129] Pegahan, S., Arakelyan, I. & Thomas, J. E. Energy-resolved information scrambling in energy-space lattices. *Phys. Rev. Lett.* **126**, 070601 (2021).
[130] Li, Q., Cui, J. & Li, W. Detecting confined and deconfined spinons in dynamical quantum simulations. *Physical Review Research* **4**, 013193 (2022).
[131] Feldmeier, J., Maskara, N., Köylüoğlu, N. U. & Lukin, M. D. Quantum simulation of dynamical gauge theories in periodically driven Rydberg atom arrays. *Preprint at https://arxiv.org/abs/2408.02733* (2024).
[132] Holevo, A. S. Bounds for the quantity of information transmitted by a quantum communication channel. *Probl. Inf. Transm.* **9**, 3–11 (1973).
[133] Holevo, A. S. Information capacity of a quantum observable. *Probl. Inf. Transm.* **48**, 1–10 (2012).
[134] Holevo, A. S. & Giovannetti, V. Quantum channels and their entropic characteristics. *Rep. Prog. Phys.* **75**, 046001 (2012).
[135] Zhuang, J.-Z., Wu, Y.-K. & Duan, L.-M. Phase-transition-like behavior in information retrieval of a quantum scrambled random circuit system. *Phys. Rev. B* **106**, 144308 (2022).
[136] Zhuang, J.-Z., Wu, Y.-K. & Duan, L.-M. Dynamical phase transitions of information flow in random quantum circuits. *Phys. Rev. Res.* **5**, L042043 (2023).
[137] Frérot, I., Naldesi, P. & Roscilde, T. Multispeed prethermalization in quantum spin models with power-law decaying interactions. *Phys. Rev. Lett.* **120**, 050401 (2018).
[138] Chen, C.-F. & Lucas, A. Finite speed of quantum scrambling with long range interactions. *Phys. Rev. Lett.* **123**, 250605 (2019).
[139] Kuwahara, T. & Saito, K. Strictly linear light cones in long-range interacting systems of arbitrary dimensions. *Phys. Rev. X* **10**, 031010 (2020).
[140] Fanchini, F. F. *et al.* Non-Markovianity through accessible information. *Phys. Rev. Lett.* **112**, 210402 (2014).

[141] Megier, N., Smirne, A. & Vacchini, B. Entropic bounds on information backflow. *Phys. Rev. Lett.* **127**, 030401 (2021).
[142] Smirne, A., Megier, N. & Vacchini, B. Holevo skew divergence for the characterization of information backflow. *Phys. Rev. A* **106**, 012205 (2022).
[143] Rivas, Á., Huelga, S. F. & Plenio, M. B. Quantum non-Markovianity: characterization, quantification and detection. *Rep. Prog. Phys.* **77**, 094001 (2014).
[144] Breuer, H.-P., Laine, E.-M., Piilo, J. & Vacchini, B. Colloquium: Non-Markovian dynamics in open quantum systems. *Rev. Mod. Phys.* **88**, 021002 (2016).
[145] de Vega, I. & Alonso, D. Dynamics of non-Markovian open quantum systems. *Rev. Mod. Phys.* **89**, 015001 (2017).
[146] Li, L., Hall, M. J. & Wiseman, H. M. Concepts of quantum non-Markovianity: A hierarchy. *Phys. Rep.* **759**, 1–51 (2018).
[147] Breuer, H.-P., Laine, E.-M. & Piilo, J. Measure for the degree of non-Markovian behavior of quantum processes in open systems. *Phys. Rev. Lett.* **103**, 210401 (2009).
[148] Fan, J. & Pang, S. Collapse and revival structure of information backflow for a central spin coupled to a finite spin bath. *Phys. Rev. A* **107**, 022209 (2023).
[149] Levine, H. *et al.* High-fidelity control and entanglement of Rydberg-atom qubits. *Phys. Rev. Lett.* **121**, 123603 (2018).
[150] De Léséleuc, S., Barredo, D., Lienhard, V., Browaeys, A. & Lahaye, T. Analysis of imperfections in the coherent optical excitation of single atoms to Rydberg states. *Phys. Rev. A* **97**, 053803 (2018).
[151] Jiang, X., Scott, J., Friesen, M. & Saffman, M. Sensitivity of quantum gate fidelity to laser phase and intensity noise. *Phys. Rev. A* **107**, 042611 (2023).
