# Supplementary material for: Observation of quantum information collapse-and-revival in a strongly-interacting Rydberg atom array
Source: Nat Commun. 2026 May 29;17:6998. doi: 10.1038/s41467-026-73520-3 (PMC13392073; doi:10.1038/s41467-026-73520-3)
Supplement: Supplementary file 2 — Transparent Peer Review File [file 41467_2026_73520_MOESM2_ESM.pdf]

nature portfolio

Peer Review File

Observation of quantum information collapse-and-revival in a strongly-interacting Rydberg atom array

Corresponding Author: Professor Lin Li

This manuscript has been previously reviewed at another journal. This document only contains information relating to versions considered at Nature Communications.

This file contains all reviewer reports in order by version, followed by all author rebuttals in order by version.

Version 0:

Reviewer comments:

Reviewer #1

(Remarks to the Author)
I reviewed this paper previously for Nature Physics, where my recommendation was a transfer to Nat Comm. I appreciate the replies of the authors to my comments. I do believe that this paper is suitable for Nature Comm. But I would ask the authors to address the following points:

- I think they should make more explicit the connection to scar dynamics. For instance, they describe in line 117 the "synchronized rotation", without linking this to scars. But this is exactly the hallmark of scars.

- To me this paper tries too hard to claim novelty (and differentiate itself from scars). I think this paper has value without the repeated claims of "novel spatio-temporal collapse-and-revival behaviour of quantum information" and similar claims. I find all these claims overstated, and the paper would profit a lot if these claims were significantly moderated.

Open Access This Peer Review File is licensed under a Creative Commons Attribution 4.0 International License, which permits use, sharing, adaptation, distribution and reproduction in any medium or format, as long as you give appropriate credit to the original author(s) and the source, provide a link to the Creative Commons license, and indicate if changes were made.
In cases where reviewers are anonymous, credit should be given to 'Anonymous Referee' and the source.
The images or other third party material in this Peer Review File are included in the article’s Creative Commons license, unless indicated otherwise in a credit line to the material. If material is not included in the article’s Creative Commons license and your intended use is not permitted by statutory regulation or exceeds the permitted use, you will need to obtain permission directly from the copyright holder.
To view a copy of this license, visit https://creativecommons.org/licenses/by/4.0/

We sincerely thank the Referee for the helpful suggestions. Below, we provide a point-by-point response to the Referee's comments. The Referee's comments are reproduced in black, and our responses are shown in blue.

Response to Referee # 1

I reviewed this paper previously for Nature Physics, where my recommendation was a transfer to Nat Comm. I appreciate the replies of the authors to my comments. I do believe that this paper is suitable for Nature Comm. But I would ask the authors to address the following points:

Response:

We thank Referee 1 for the positive overall assessment of our work and for recommending the transfer to Nature Communications. In response to the referee's suggestions, we have revised the manuscript accordingly.

- I think they should make more explicit the connection to scar dynamics. For instance, they describe in line 117 the "synchronized rotation", without linking this to scars. But this is exactly the hallmark of scars.

Response:

In response to the referee's suggestion, we revised the manuscript to make the connection to scar dynamics more explicit. In particular, the sentence in line 117 has been changed to: "This synchronized evolution, **which is a hallmark of scar dynamics,** persists until boundary effects gradually alter the spin rotations from the outer edge inwards." We also removed the phrase "beyond the scope of many-body scars" from line 49.

- To me this paper tries too hard to claim novelty (and differentiate itself from scars). I think this paper has value without the repeated claims of "novel spatio-temporal collapse-and-revival behaviour of quantum information" and similar claims. I find all these claims overstated, and the paper would profit a lot if these claims were significantly moderated.

Response:

We appreciate this constructive suggestion and agree that our experimental results stand on their own. Accordingly, we have significantly moderated the novelty-related wording throughout the manuscript. Specifically, we removed "novel" from the highlighted phrase "spatio-temporal collapse-and-revival behaviour". We also removed several other instances of "novel", "unusual", and "unique".
